# A proactive/reactive mass screening approach with uncertain symptomatic cases

**DOI:** 10.1371/journal.pcbi.1012308

**Published:** 2024-08-14

**Authors:** Jiayi Lin, Hrayer Aprahamian, George Golovko

**Affiliations:** 1 Department of Industrial and Systems Engineering, Texas A&M University College Station, Texas, United States of America; 2 Department of Pharmacology and Toxicology, The University of Texas Medical Branch Galveston, Texas, United States of America; Stockholms Universitet, SWEDEN

## Abstract

We study the problem of mass screening of heterogeneous populations under limited testing budget. Mass screening is an essential tool that arises in various settings, e.g., the COVID-19 pandemic. The objective of mass screening is to classify the entire population as positive or negative for a disease as efficiently and accurately as possible. Under limited budget, testing facilities need to allocate a portion of the budget to target sub-populations (i.e., proactive screening) while reserving the remaining budget to screen for symptomatic cases (i.e., reactive screening). This paper addresses this decision problem by taking advantage of accessible population-level risk information to identify the optimal set of sub-populations for proactive/reactive screening. The framework also incorporates two widely used testing schemes: Individual and Dorfman group testing. By leveraging the special structure of the resulting bilinear optimization problem, we identify key structural properties, which in turn enable us to develop efficient solution schemes. Furthermore, we extend the model to accommodate customized testing schemes across different sub-populations and introduce a highly efficient heuristic solution algorithm for the generalized model. We conduct a comprehensive case study on COVID-19 in the US, utilizing geographically-based data. Numerical results demonstrate a significant improvement of up to 52% in total misclassifications compared to conventional screening strategies. In addition, our case study offers valuable managerial insights regarding the allocation of proactive/reactive measures and budget across diverse geographic regions.

## Author summary

The problem of mass screening of heterogeneous populations is challenging, especially when we do not have enough tests to check everyone all at once. Therefore, under limited testing budget, testing facilities need to allocate a portion of the testing budget to target sub-populations (i.e., proactive screening) while reserving the remaining budget to screen for symptomatic cases (i.e., reactive screening). Our study provides a strategy to make those decision efficiently by taking advantage of risk information and utilizing optimization models. We conduct a comprehensive case study on COVID-19 in the US and numerical results demonstrate a significant improvement compared to conventional screening strategies. The results of this paper will benefit health administrators and policy-makers by providing managerial insights regarding optimal allocation of testing kits and testing designs across diverse geographic regions.

## 1 Introduction

### 1.1 Motivation and background

Mass screening involves testing a large population to detect a specific disease, aiming to accurately classify the entire population as either positive or negative for the disease. It has become a widely used approach across various healthcare settings. Examples of mass screening include the detection of cancers [1–3], sexually transmitted diseases (STDs) [4–6], and ensuring blood transfusion safety [7–9]. In recent years, mass screening has gained significant attention and has been increasingly implemented in response to the COVID-19 pandemic. A number of studies have consistently shown the efficacy of mass screening for COVID-19 in various real-world scenarios [10–13]. For example, New Zealand has garnered widespread recognition for its highly effective strategy against COVID-19 during the initial phases of the pandemic, resulting in an impressively low number of confirmed cases and fatalities. The country swiftly executed mass screenings for a population of only 5 million, reaching more than 150, 000 individuals in a relatively short period of time, with subsequent contact tracing implemented for each case [11]. The example clearly demonstrates that effective mass screening programs can identify infected individuals and then provide valuable information for implementing other preventive measures, such as quarantining and contact tracing. As such, mass screening plays a fundamental role in breaking the chain of transmission and controlling the spread of the disease, particularly within the context of highly infectious diseases. Thus, it is of utmost importance for decision makers to develop an effective screening strategy that provides accurate identification of infected cases.

The most straightforward screening strategy is to test every individual in the population. However, this approach is often hampered by inadequate testing resources that cannot meet the high demand for testing. Limited testing resources can be attributed to various factors, with the most common being the limited availability of diagnostic testing assays. For instance, in many tropical regions where Malaria is prevalent, diagnostic testing methods like microscopy or rapid diagnostic tests (RDTs) may not be readily accessible in remote areas [14]. This limitation is particularly evident during the early stages of a new disease outbreak. For example, during the early stages of the COVID-19 outbreak, the United States faced a significant shortage of testing assays, with a weekly testing capacity of only 0.36% of the entire population [15]. Additionally, resources can also be constrained due to financial limitations, inadequate infrastructure, and other factors. Therefore, how to utilize limited testing budget to maximize the effectiveness of mass screening becomes particularly crucial and relevant. This research problem becomes trivial when considering the inherent homogeneity within the entire population. In other words, if all individuals were identical, it would be equivalent to conducting random testing on subjects. However, in practice, populations are highly heterogeneous. That is, different sub-populations display potential variations in terms of risk scores, health conditions, symptomatic rates and other factors, thereby amplifying the complexity of the problem.

A commonly adopted strategy is to screen individuals who exhibit symptoms, referred to as *reactive screening*. This approach allows for the confirmation of their infectious status, which is especially important at the individual level, as it ensures that infected individuals receive timely and appropriate medical treatment. However, at the population level, reactive screening may not always suffice to effectively control disease transmission, especially in the case of highly infectious diseases. This limitation arises due to several factors. First, disease transmission can occur from presymptomatic cases (i.e., infected individuals that are infectious before developing symptoms). For example, studies have revealed that approximately 35% of COVID-19 transmissions were attributed to presymptomatic cases [16]. Therefore, the early identification of these individuals, coupled with the prompt implementation of essential precautions, can play a crucial role in effectively mitigating the spread of the disease. Similarly, asymptomatic cases (i,e., infected subjects that never exhibit any symptoms) can also contribute to disease transmission, and an accurate identification of these cases is vital in order to effectively prevent disease outbreaks. In the case of COVID-19, research suggests that approximately 30% of all infected cases are asymptomatic [17]. Given these drawbacks of a reactive screening strategy, *proactive screening* has emerged as an attractive alternative strategy that overcomes some of these shortcomings. This approach involves screening individuals before they develop significant clinical symptoms or become major contributors to widespread transmission. By implementing proactive screening, it becomes possible to identify and isolate potentially infectious individuals at an early stage, effectively curbing the spread of the disease. Therefore, it is essential to prioritize individuals who are most in need of screening. For instance, these individuals can include those in high-exposure rate occupations (e.g., healthcare workers) or those who are more vulnerable to the disease (e.g., individuals with pre-existing health conditions).

While proactive screening is critical, the significance of reactive screening is equally essential and cannot be simply overlooked. This is especially important in settings where other diseases share similar symptoms with the targeted disease. For example, the flu can manifest a range of mild to severe symptoms, such as coughing, fever, and fatigue, which are often indistinguishable from those of COVID-19. However, COVID-19 is a more contagious and can lead to long-term health complications [18, 19]. Therefore, it is crucial to establish a comprehensive framework that integrates both proactive and reactive screening to ensure the accurate identification and effective containment of diseases. Relying solely on one screening measure can result in undesirable outcomes. Thus, it is imperative to determine an appropriate allocation of the available budget between proactive and reactive screening in order to maximize their combined effectiveness. This problem becomes particularly intriguing when dealing with heterogeneous populations. For instance, optimal screening strategies may implement proactive screening for sub-populations with higher risks and reactive screening for those with lower risks. Furthermore, even when considering the same prevalence rate, different sub-populations can exhibit varying rates of symptomatic cases due to variations in health conditions resulting from factors such as age or pre-existing health conditions. In such cases, it may be optimal to employ proactive screening for sub-populations that are less likely to experience severe symptoms, while utilizing reactive screening for those sub-populations that are more vulnerable. By tailoring the screening approach to the specific characteristics and needs of different sub-populations, we can optimize the allocation of resources and enhance the overall effectiveness of the screening strategy.

Regardless of the *testing manners* (either proactive or reactive screening), a shared important decision problem is testing schemes. The most commonly adopted scheme is individual testing, which involves testing each subject individually. Although individual testing is simple and easy to implement, it may only be feasible for screening a small portion of the population when the budget is limited, which significantly restricts the screening efforts. To overcome this limitation, an alternative testing scheme is *Dorfman group testing* [20], which involves testing multiple subjects simultaneously with a single test. Dorfman testing has two stages: In the first, specimens from multiple individuals are mixed together and tested in a single group. If the test result is negative, every subject in the group is classified as negative and the procedure stops. If the test result is positive, then each subject of the group is tested again individually in the second stage to identify the positive members of the group. Such a procedure has the potential to significantly expand testing capacity and it has been widely utilized in a variety of settings (e.g., blood screening [21, 22], HIV screening [23, 24], COVID-19 screening [15, 25]). Group testing is a strategic and effective measure that can lead to considerable economies in terms of reagents, labor, and time, ultimately enhancing the speed and efficiency of laboratory outputs: First, a PCR test, which is currently the benchmark for detecting SARS-CoV-2 (the virus responsible for COVID-19), typically requires around two hours to process [26, 27]. Consequently, the strategy of group testing, supplemented by individual tests when necessary, is viable in terms of time efficiency. Additionally, the capacity of testing machines plays a crucial role, often capping the number of samples that can be processed per cycle, with a common threshold being 96 samples per run [26]. Group testing enhances the total number of samples examined in each cycle, thereby boosting overall throughput and reducing the average time needed to obtain results. This increase in processing capacity through pooling can facilitate broader COVID-19 screening initiatives. Given the usefulness of group testing, group testing for COVID-19 has received emergency use authorization from the U.S. Food and Drug Administration in July 2020 [28]. Subsequently, numerous communities and testing facilities across the U.S. have embraced this method, significantly enhancing the nation’s testing capabilities [29–34]. For example, the Nebraska Public Health Lab leveraged group testing to quadruple its COVID-19 screening capacity. [29]. In Massachusetts, schools implemented this strategy, enabling broader surveillance of COVID-19 while conserving resources [32]. Similarly, Duke University in North Carolina also adopted group testing, facilitating frequent testing for 10,265 students, who underwent a total of 68,913 tests, despite limited resources. [31]. Countries across Asia, Europe, South America and Africa have also swiftly adopted this approach within various community settings (e.g., universities, hospitals, and care homes) [35–41]. In addition, extensive research and studies have consistently demonstrated the effectiveness and practicality of group testing for COVID-19 [42–46]. However, in certain situations, group testing, while generally cheaper, may provide poor performance compared to individual testing, especially when the prevalence rate is high. Consequently, it is advantageous to incorporate both testing schemes and customize the screening strategy based on different circumstances.

In this paper, we aim to establish an optimization-based targeted screening strategy under limited testing budget. The proposed framework also addresses the challenge of high heterogeneity within the population, which consists of various sub-populations with distinct risk scores, symptomatic rates, and testing needs. Moreover, it incorporates both proactive and reactive screening approaches, as well as different testing schemes simultaneously. Although there are several variations of group testing schemes, including array-based [47, 48], adaptive [49, 50], and multi-stage [51, 52], these methods are often complicated to implement in practice. Therefore, in this paper, we focus on the two most commonly adopted testing schemes: Individual and Dorfman group testing, which provide more feasible and actionable insights. The comprehensive design of our proposed screening strategy offers a highly customized approach that optimizes the overall classification accuracy. Such a strategy not only enhances the accuracy of screening but also provides valuable managerial insights. For instance, it allows for the distribution of limited testing assays and testing budget for proactive and reactive screening, enabling effective resource management. This established framework can be widely applied in mass screening programs for various diseases, thereby improving readiness to combat new infectious disease threats.

### 1.2 Literature review

The existing literature on mass screening is diverse and extensive. The vast majority of the existing body of work, however, either imposes the unrealistic assumption such as perfect tests [53–55] or a homogeneous population [48, 51, 56]. Although some studies integrate heterogeneity into their frameworks, the majority of the research places emphasis on reactive screening. In this context, the subjects for testing have already been selected, and the focus is on exploring optimal screening strategies for this predefined group of subjects [51, 55, 57–60]. For instance, [58] investigate the optimal screening strategies for specimens collected at healthcare clinics. While the studies in question offer valuable insights, they overlook the benefits of proactive screening and fail to integrate the challenge of identifying which sub-populations to target into the decision-making framework.

Considering the pitfalls of reactive screening, proactive screening has gained increasing attention [61–67]. One stream of research utilizes epidemiological models to simulate the spread of diseases under different targeted screening strategies [62, 64, 68–70]. For instance, the prevailing approach in the field revolves around the utilization of epidemiological models such as the Susceptible, Infected, and Recovered (SIR) compartmental model [62, 64, 68–71]. While these studies are capable of evaluating the effectiveness of various targeted screening policies, they often rely on a trial-and-error approach to identify optimal policies. This computational process can be prohibitively time-consuming or even infeasible in certain cases.

In an effort to overcome this limitation, another line of work focuses on optimization-based approaches to identify optimal targeted screening policies [72–76]. For instance, [72] utilize population-level attributes such as age, race, and nationality to model the issue as a multi-armed bandit problem. Their adoption of a reinforcement learning framework illuminates the structure of nearly optimal strategies. Although promising, their work has some key limitations, such as the assumption of a homogeneous population and perfect tests. Furthermore, as observed in the aforementioned studies and other similar works within an optimization framework [73], their methodology does not incorporate the design of group testing (e.g., determining the optimal group size) into the decision-making process. This limitation significantly hinders screening efforts under scarce testing capacity. While another study, aligning more closely with our framework, adopts a more tailored screening strategy [74], it exclusively studies proactive screening methods. This focus markedly simplifies the modeling complexity, as they formulate the problem as a linear program.

To the best of our knowledge, there has not been much effort put towards incorporating both screening manners (proactive and reactive screening). Therefore, we perceive our work as a valuable contribution that aims to bridge this gap and overcome certain limitations and unrealistic assumptions. These include assuming a homogeneous population, relying on perfect tests, and considering only a single testing scheme (primarily individual testing) [53, 64, 71]. In this paper, we aim to establish an optimization-based targeted screening strategy under limited testing budget. The proposed framework also addresses the challenge of high heterogeneity within the population, which consists of various sub-populations with distinct risk scores and symptomatic rates. It also incorporates diverse testing manners (proactive and reactive screening) and different testing schemes simultaneously, culminating in a more intricate and comprehensive optimization model (specifically, a mixed-integer nonlinear model).

### 1.3 Contributions

In summary, the contributions of this paper are multi-fold: First, we introduce an optimization-based screening strategy under limited testing budget that incorporates two testing schemes: Individual and Dorfman group testing. Our proposed approach addresses common unrealistic assumptions, such as perfect tests and homogeneous populations. More importantly, the framework accommodates both proactive and reactive screening, taking into account symptomatic information regarding other diseases that share similar symptoms. This enables the identification of sub-populations for proactive screening while also determining the appropriate allocation for reactive screening. To the best of our knowledge, this is the first work that integrates all these factors and dimensions into a comprehensive framework. Second, our analysis of the resulting problem, which is a mixed-integer nonlinear program, reveals structural properties that enable us to develop an efficient globally optimal solution scheme. Specifically, we first identify crucial properties that allow us to determine the optimal values for integer decision variables in advance, significantly reducing the complexity. Subsequently, we capitalize on the special knapsack-style structure of the resulting bilinear program and devise an efficient solution scheme. Furthermore, we extend the model by incorporating customized testing schemes across various risk categories. We also introduce a highly efficient solution algorithm for the generalized model. Numerical experiments provide compelling evidence of the effectiveness of the solution scheme, showcasing its efficiency over commercial solvers by significantly reducing computational time to seconds and consistently delivering near-optimal solutions. Third, we conduct a case study on COVID-19 in the US using real geographically-based data. Our numerical results show our solution significantly outperforms conventional screening policies, reducing misclassifications by 21% and 27% across two distinct datasets, highlighting its robust performance. This highlights the importance of data-driven screening policies. Moreover, our findings offer valuable managerial insights regarding optimal testing schemes, the distribution of reactive/proactive screening, and budget allocation across various geographic areas. Lastly, we evaluate the performance of generalized model that integrates tailored testing strategies across diverse risk categories and discuss the trade-offs between performance and practicality.

The remainder of this paper is organized as follows: Section 2 introduces the notation and provides a clear definition of the decision problem (summarized in Table 1) and optimization model. Following that, Section 3 discusses the methodological results and presents an efficient solution scheme. Additionally, Section 3 provides a more general model along with its solution strategy. Then, Section 4 presents findings from a COVID-19 case study in the US. Lastly, Section 5 concludes the paper and highlights potential future research directions. To enhance readability, all proofs and some discussions are relegated to the S1 File.

**Table 1:** Notation.

Parameters		
$L_{m}$	≜	The set of individuals belonging to category m∈M.
L	≜	A population consisting of *M* categories $L_{m}$.
*a*_*m*_	≜	The number of active positive cases in the category m∈M.
*p*_*m*_	≜	The total number of subjects in the category m∈M.
*r*_*m*_	≜	The risk of subjects in the category m∈M.
*f*_*m*_	≜	The probability of having concurrent diseases in the category m∈M.
*s*_*m*_	≜	The symptomatic rate of the primary disease in the category m∈M.
*α*_*m*_	≜	The symptomatic rate of other diseases that share similar symptoms in the category m∈M.
*P*_*T*_	≜	The total population size across all categories.
*Se*	≜	Sensitivity of test.
*Sp*	≜	Specificity of test.
*L*_*m*_	≜	The probability of a subject experiencing symptoms in category m∈M.
*J*_*m*_	≜	The probability of a subject in category *m* is infected, given the subject is showing symptoms.
*H*_*m*_	≜	The probability of a subject in category *m* is infected, given the subject is not showing symptoms.
*B*	≜	The given testing budget.
λ_*m*_	≜	The weight placed on false negatives in category *m*.
*N* ^*p*^	≜	Upper bound on the group size of proactive screening.
*N* ^*r*^	≜	Upper bound on the group size of reactive screening.
Random Variables		
*FN*	≜	Number of false negatives.
*FP*	≜	Number of false positives.
*TC*	≜	Total testing costs.
Decision Variables (**RP-MS**)		
$x_{m}^{p}$	≜	The proportion of subjects in category m∈M that are proactively tested.
$x_{m}^{r}$	≜	The remaining proportion of subjects without undergoing proactive screening who exhibit symptoms and are reactively tested in category m∈M.
*n* ^*p*^	≜	The group size of proactive and proactive screening.
*n* ^*r*^	≜	The group size of proactive and reactive screening.
$z_{m}^{s}$	≜	The proportion of untested symptomatic subjects classified as positives in category *m*.
$z_{m}^{\bar{s}}$	≜	The proportion of untested non-symptomatic subjects classified as positives in category *m*.
Decision Variables (**CRP-MS**)		
$u_{m}^{n^{p}}$	≜	The proportion of subjects in category *m* that are proactively tested with group size *n*^*p*^.
$v_{m}^{n^{r}}$	≜	The proportion of subjects in category *m* that are reactively tested with group size *n*^*p*^.

## 2 Notation, decision problem, and optimization model

Consider a population that comprises *M* categories, each representing a sub-population with a potentially different risk, i.e., probability of a subject from a category having the disease. The categories collectively represent the entire population. Let $L=\{L_{1},\cdots ,L_{M}\}$ denote the entire population where $L_{m}$ represents the set of subjects in category m∈M={1,…,M}. The decision on how to partition the population into different categories is crucial as it ultimately impacts the overall effectiveness of the testing strategy. The aim is to define the categories in a way that maximizes heterogeneity between them while minimizing heterogeneity within each category. While considering each individual as a category would be most ideal, it is often impractical due to difficulties in assessing individual risk, particularly in large populations. Additionally, factors such as research objectives, available data, and population characteristics can influence the definition of these categories. The most prevalent approach involves utilizing clustering methods where the categories are distinctly separate from one another [77–79]. In fact, [80] has proposed an efficient partition scheme to maximize the heterogeneity between sub-populations. Given the presence of these methods in the literature, one viable approach could be to partition the population beforehand and subsequently apply our model to devise the optimal screening strategy. However, this sequential method of analysis might result in sub-optimal solutions.

The actual risk of disease is determined by a wide range of factors, including exposure risk (such as close contact, travel history, and occupational exposure), demographic information (e.g., age, race, and sex), behavioral factors (e.g., wearing masks, practicing good hand hygiene, and maintaining physical distancing), as well as community transmission levels. Incorporating numerous factors poses a challenge, often compounded by limited data availability. As a result, accurately integrating all these variables to estimate the actual risk of the disease is exceedingly difficult, potentially even impossible. In this paper, we make the assumption that the categories are pre-defined and we use several metrics to characterize each category m∈M. First, let *a*_*m*_ and $p_{m}=\left|L_{m}\right|$ denote the number of active cases of the disease and the population size in category *m*, respectively. By considering both the number of active cases and the population size, we can assess the corresponding risk *r*_*m*_ as *r*_*m*_ = *a*_*m*_/*p*_*m*_ ∈ [0, 1]. The number of active cases, in fact, is influenced by various factors mentioned above (e.g., exposure risk and behavioral factors). Thus, including active cases in our risk estimates implicitly accounts for these factors. In addition, this data is usually publicly available and easy to access. Furthermore, this formula with the number of active cases essentially quantifies the prevalence rate, a key metric for characterizing the community’s risk level. The prevalence rate is a pivotal metric widely utilized by public health authorities, including the Centers for Disease Control and Prevention (CDC). It provides a quantifiable measure of community transmission, reflecting the proportion of the population affected by active cases at a given moment. Therefore, this rate is indicative of the immediate extent of disease spread within the community, serving as a critical indicator of the current public health situation. In addition, we designate the disease that needs to be detected as the primary disease, while diseases that share similar symptoms are referred to as concurrent diseases. These concurrent diseases exhibit similar symptoms to the primary disease, making it difficult to distinguish them based on symptoms alone. Let *f*_*m*_ ∈ [0, 1] denote the risk of concurrent diseases, representing the probability of a subject from category *m* having concurrent diseases. Furthermore, we define various symptomatic rates as follows: The symptomatic rate of the primary disease is denoted as *s*_*m*_, whereas the symptomatic rate of other diseases that share similar symptoms is denoted as *α*_*m*_. When a subject is exposed to an infectious disease, it is possible for the subject to still be susceptible to other diseases [81]. Therefore, we assume that the infection by different diseases (both primary and concurrent) are independent. Lastly, let $P_{T}=\sum_{m\in M}p_{m}$ represent the total population size across all categories.

A testing assay is often available to screen the population for the disease. However, the test is imperfect with *sensitivity* (i.e., true positive probability), denoted by *Se* ∈ [0, 1], and *specificity* (i.e., true negative probability), denoted by *Sp* ∈ [0, 1]. Without loss of generality, we assume that the test’s true negative probability is higher than its false negative probability, i.e., *Sp*/(1 − *Se*) ≥ 1. If this assumption is not satisfied, we can improve the test accuracy by interpreting the results in the opposite way, satisfying the aforementioned condition. Regarding testing schemes, we focus on two specific testing schemes: Individual and Dorfman group testing. In addition, testing can be implemented in either a proactive or reactive manner. Proactive screening refers to testing individuals before any clinical signs or recognizable symptoms. On the other hand, reactive screening is conducted for symptomatic cases, with the goal of identifying the true infectivity status and taking appropriate medical measures accordingly. The decision problem, then, is to identify categories (or portions of categories) of the heterogeneous population that should be screened either in a proactive and/or reactive manner. In addition, the testing scheme (i.e., individual or Dorfman testing) as well as the testing design (i.e., the group size) need to be determined for tested categories. This decision must be made while also considering the limited budget availability and ensuring that the testing strategy’s overall accuracy is optimized.

In this paper, we evaluate the accuracy of the testing strategy by measuring the total number of misclassifications. In particular, we use two metrics to characterize these misclassifications: (i) the expected number of false negatives, denoted by E[FN] and (ii) the expected number of false positives, denoted by E[FP]. The expectation is taken over the true infectivity status of subjects which is unknown. The relative significance of these two metrics can vary, sometimes significantly, based on the specific context or disease involved. In fact, even within the same context and disease, the importance of these metrics can shift over time. For instance, in the scenario of a highly infectious disease outbreak, like COVID-19, the focus on the terms of the objective function may change during different phases of the outbreak. In the initial stages, prioritizing false negatives could be crucial to prevent further spread and better manage containment, as undetected cases can lead to widespread transmission. As the situation progresses towards a post-outbreak phase with potential herd immunity, the focus may shift to minimizing false positives to alleviate unnecessary quarantines, contact tracing, and their associated economic and social disruptions. As another example of situations in which false positives are prioritized, consider a situation where a false positive lead to costly additional procedures, such as extensive testing (e.g., full-body CT or MRI scans), particularly at the onset of a pandemic when effective treatment or vaccination protection is unavailable. Given the aforementioned arguments, it is crucial to adopt a tunable objective function (i.e., a weighted sum of the two classification errors), providing decision-makers the flexibility to tailor the optimization model to their specific circumstances. This robust framework promotes adaptability, efficiently addressing diverse urgencies and requirements to meet specific objectives in a variety of situations. We believe that this enhances the model’s utility and adaptability, enabling its effective application across a range of diseases and contexts. In addition, categories are heterogeneous and will have different needs and consideration towards negatives/positives. For example, consider the scenario of mass screening before a scheduled event: A false positive result might prevent individuals from attending, leading to varied consequences based on individual utility and their roles. Therefore, the weight parameter should reflect the non-uniform impact of false positives/negatives among different population groups. Employing a fixed λ to weigh false negatives uniformly for the entire population overlooks significant aspects of heterogeneity within these groups. As a result, we advocate for a model that acknowledges and incorporates the unequal effects of false negatives/positives by introducing a weight parameter λ_*m*_ for each category m∈M, enhancing both the realism and efficacy of the approach. By incorporating these weight parameters, we can construct a more customized model that is capable of handling various situations and needs of each category.

Due to limited budget, it may not be feasible to screen the entire population, resulting in some individuals remaining untested. Misclassification can also occur when untested subjects are falsely classified based on the designated classification policy. For these untested subjects, we integrate the classification policy’s decision into the decision-making process that specifies whether untested subjects should be classified as positive or negative. Moreover, as symptomatic cases may have a higher chance of being true positives, it becomes imperative to incorporate two distinct classification policies based on the symptomatic status of untested individuals. The flexibility in the classification policy is critical because it can significantly impact the overall accuracy of the testing strategy.

We now formulate the decision problem in a more rigorous manner. Let $x_{m}^{p}\in \left[0,1\right]$ represent the proportion of subjects in category m∈M that are proactively tested. On the other hand, let $x_{m}^{r}$ represent the remaining proportion of subjects without undergoing proactive screening who exhibit symptoms and are reactively tested in category m∈M. An assumption we enforced is that each individual can only undergo testing once, either through proactive or reactive screening. This implies that if an individual is selected for proactive screening, they are exempted from undergoing reactive screening, even if they exhibit symptoms later on. This assumption is reasonable since, with a short screening cycle, if a subject shows symptoms shortly after testing, their condition can be inferred from the earlier proactive screening results. Thus, it is more valuable to refrain from re-screening individuals who have already been proactively tested, as it conserves scarce screening resources for other subjects who have not yet been tested. In terms of testing schemes, the group size of proactive and reactive screening is denoted by *n*^*p*^ and *n*^*r*^ respectively. Individual testing can be seen as a special case of group testing when *n*^*p*^ and *n*^*r*^ are equal to 1. Thus, utilizing *n*^*p*^ and *n*^*r*^ can fully characterize the testing schemes. Note that we employ a testing scheme for proactive/reactive screening across different categories, respectively. This setting offers more practical insights since implementing a combination of two testing schemes coupled with two testing manners (proactive or reactive screening) across different categories may pose challenges in practice. However, the more generalized model, which incorporates different testing schemes across categories, will be discussed and studied in Section 3.1. The group size cannot be any arbitrary positive integer due to various practical constraints. One such limitation is the dilution effect of grouping, where the specimen of an infected individual is diluted when grouped with negative samples, thereby affecting the accuracy of the testing assay [82]. However, studies have shown that the dilution effect becomes negligible when the group size is not too large [83, 84]. Another constraint is the capacity of the testing equipment, which can limit the size of the groups that can be processed at one time [26]. Therefore, we impose an upper bound *N*^*p*^ and *N*^*r*^ for *n*^*p*^ and *n*^*r*^, respectively (i.e., *n*^*p*^ ∈ {1, …, *N*^*p*^} and *n*^*r*^ ∈ {1, …, *N*^*r*^}). Lastly, for untested subjects, let the binary decision variable $z_{m}^{s}\;\left(z_{m}^{\bar{s}}\right)\in \left[0,1\right]$ represent the proportion of untested symptomatic (non-symptomatic) subjects that will be classified as positives. To facilitate the presentation, we use boldface to represent the column vector forms of all variables, i.e., $x^{p}={\left[x_{m}^{p}\right]}_{m\in M}$, $x^{r}={\left[x_{m}^{r}\right]}_{m\in M}$, $z^{s}={\left[z_{m}^{s}\right]}_{m\in M}$ and $z^{\bar{s}}={\left[z_{m}^{\bar{s}}\right]}_{m\in M}$. A screening strategy is thus fully characterized by the 3-tuple (***x***, ***n***, ***z***) where ***x*** = [***x***^*p*^, ***x***^*r*^], ***n*** = [*n*^*p*^, *n*^*r*^], and $z=[z^{s},z^{\bar{s}}]$.

Recall that our objective is to identify a screening strategy that maximizes testing accuracy under a budget constraint. In particular, we consider a formulation in which a weighted sum of the expected number of false negatives and false positives is minimized. This is formally presented in the following optimization problem:

$$\begin{array}{cc} \underset{x,n,z}{\text{minimize}}\; & \sum_{m=1}^{M}[\lambda_{m}E_{m}\left[FN\left(x,n,z\right)\right]+\left(1-\lambda_{m}\right)E_{m}\left[FP\left(x,n,z\right)\right]]\frac{p_{m}}{P_{T}}\\ \text{subject}\;\text{to}\; & E[TC(x,n,z)]\leq B\\ & x_{m}^{p},x_{m}^{r}\in \left[0,1\right],\;\forall \;m\in M\\ & z_{m}^{s},z_{m}^{\bar{s}}\in \left[0,1\right]\;,\forall \;m\in M\\ & n^{p}\in \left\{1,\ldots ,N^{p}\right\},n^{r}\in \left\{1,\ldots ,N^{r}\right\}, \end{array}$$

where $E_{m}\left[FN\left(\cdot \right)\right]$, and $E_{m}\left[FP\left(\cdot \right)\right]$, $E_{m}\left[TC\left(\cdot \right)\right]$ respectively represent the expected number of false negatives, false positives, and testing cost per subject in category *m*. The weight λ_*m*_ ∈ [0, 1] is a user-defined weight parameter that emphasizes the importance of false negatives relative to false positives in category *m*. The objective is a weighted sum of two classification metrics that arise from both tested and untested subjects across all categories in proportion to their population sizes. The first constraint set guarantees that the total expected cost is within the given available testing budget, denoted by *B* ∈ [0, 1], which represents the ratio of the number of available tests to the total population size. For example, *B* = 0.2 indicates that the current budget allows 20% of the population to be individually tested.

Before introducing the expressions for the performance measures, it is necessary to define *L*_*m*_, *J*_*m*_, and *H*_*m*_, which will be utilized in the subsequent performance measures (the detailed derivation of these expressions can be found in Section B in S1 File):

$$\begin{array}{cc} L_{m} & =a_{m}f_{m}+\left(1-a_{m}f_{m}\right)s_{m}r_{m},\;J_{m}=\frac{L_{m}-\alpha_{m}f_{m}\left(1-r_{m}\right)}{L_{m}}\;\text{and}\\ \;H_{m} & =\frac{\left(1-s_{m}\right)\left(1+\alpha_{m}f_{m}\right)r_{m}}{1-L_{m}}. \end{array}$$

Since the derivation process of the performance measures is quite involved, we summarize the final expression of the performance measures in the following proposition. Essentially, *L*_*m*_ represents the probability of a subject experiencing symptoms in category *m*, and *J*_*m*_ (*H*_*m*_) denote the probability of a subject in category *m* is infected, given the subject is showing (not showing) symptoms.

**Proposition 1**. *For a given screening strategy* (***x***, ***n***, ***z***) *and category* m∈M, *the following identities hold*:

*(i) The expected number of false negatives arising from category m*, $E_{m}\left[FN\left(x,n,z\right)\right]$ *is given by*:
  $$\begin{array}{cc} E_{m}\left[FN\left(x,n,z\right)\right] & =f_{FN}\left(n^{p}\right)r_{m}x_{m}^{p}\\ & \;+[(f_{FN}\left(n^{r}\right)J_{m}x_{m}^{r}+\left(1-z_{m}^{s}\right)\left(1-J_{m}\right)\left(1-x_{m}^{r}\right))l_{m}+\left(1-z_{m}^{\bar{s}}\right)H_{m}]\left(1-x_{m}^{p}\right), \end{array}$$
  *where*
  $$\begin{array}{c} f_{FP}^{m}\left(n\right)=\{\begin{array}{ll} 1-Sp, & if\;n=1\\ Se(1-Sp)-(1-Sp)(Se+Sp-1){\left(1-r_{m}\right)}^{n-1}, & if\;n\geq 2. \end{array} \end{array}$$
*(ii) The expected number of false positives arising from category m*, $E_{m}\left[FP\left(x,n,z\right)\right]$ *is given by*:
  $$\begin{array}{cc} E_{m}\left[FP\left(x,n,z\right)\right] & =f_{FP}^{m}\left(n^{p}\right)\left(1-r_{m}\right)x_{m}^{p}\\ & \;+[z_{m}^{\bar{s}}\left(1-H_{m}\right)\left(1-l_{m}\right)+(f_{FP}\left(n^{r}\right)\left(1-J_{m}\right)x_{m}^{r}+z_{m}^{s}\left(1-J_{m}\right)\left(1-x_{m}^{r}\right))l_{m}]\left(1-x_{m}^{p}\right), \end{array}$$
  *where the expression of* $f_{FP}^{m}$ *is given by*
  $$\begin{array}{c} f_{FP}^{m}\left(n\right)=\{\begin{array}{ll} 1-Sp, & if\;n=1\\ Se(1-Sp)-(1-Sp)(Se+Sp-1){\left(1-r_{m}\right)}^{n-1}, & if\;n\geq 2. \end{array} \end{array}$$
*(iii) The expected number of testing cost arising from category m*, $E_{m}\left[TC\left(x,n,z\right)\right]$ *is given by*:
  $$\begin{array}{c} E_{m}\left[TC\left(x,n,z\right)\right]=f_{TC}^{m}\left(n^{p}\right)x_{m}^{p}+g_{TC}^{m}\left(n^{r}\right)l_{m}x_{m}^{r}\left(1-x_{m}^{p}\right), \end{array}$$
  *where*
  $$\begin{array}{c} f_{TC}^{m}\left(n\right)=\{\begin{array}{ll} 1, & if\;n=1\\ \frac{1}{n}+Se-(Se+Sp-1){\left(1-r_{m}\right)}^{n}, & if\;n\geq 2, \end{array} \end{array}$$
  *and*
  $$\begin{array}{c} g_{TC}^{m}\left(n\right)=\{\begin{array}{ll} 1, & if\;n=1\\ J_{m}\left(\frac{1}{n}+Se\right)+(1-J_{m})\left(1+\left(\frac{1}{n}-Sp\right){\left(1-r_{m}\right)}^{n-1}\right), & if\;n\geq 2. \end{array} \end{array}$$

By substituting the expressions of these performance measures into the optimization model, we present the **Reactive Proactive Mass Screening (RP-MS)** problem:

$$\begin{array}{ll} \underset{x,n,z}{\text{minimize}} & A{\left(n^{p}\right)}^{⊤}x^{p}+{\left(I-x^{p}\right)}^{⊤}Q(n^{r})x^{r}+{\left(I-x^{p}\right)}^{⊤}V(z^{s})(I-x^{r})+{\left(I-x^{p}\right)}^{⊤}C(z^{\bar{s}})\\ \text{subject}\;\text{to} & A^{c}{\left(n^{p}\right)}^{⊤}x^{p}+{\left(I-x^{p}\right)}^{⊤}Q^{c}(n^{r})x^{r}\leq B\\ & \gamma (1-x_{m}^{p})\geq x_{m}^{r},\;\forall \;m\in M\\ & x_{m}^{p},x_{m}^{r},z_{m}^{s},z_{m}^{\bar{s}}\in [0,1],\;\forall \;m\in M\\ & n^{p}\in \{1,\ldots ,N^{p}\},n^{r}\in \{1,\ldots ,N^{r}\}, \end{array}$$ (RP-MS)

where ***I*** = [1, 1, …, 1]^⊤^ is an all-one vector with *M* entries, ***A***(*n*^*p*^) = [*A*_1_(*n*^*p*^), …, *A*_*M*_(*n*^*p*^)]^⊤^, $C\left(z^{\bar{s}}\right)={\left[C_{1}\left(z_{1}^{\bar{s}}\right),\ldots ,C_{M}\left(z_{M}^{\bar{s}}\right)\right]}^{⊤}$ and $A^{c}\left(n^{p}\right)={\left[A_{1}^{c}\left(n^{p}\right),\ldots ,A_{M}^{c}\left(n^{p}\right)\right]}^{⊤}$. In addition, we define diagonal matrices ***Q***(*n*^*r*^) = *diag* (*D*_1_(*n*^*r*^), …, *D*_*M*_(*n*^*r*^)), $Q^{c}\left(n^{r}\right)=diag\left(D_{1}^{c}\left(n^{r}\right),\ldots ,D_{M}^{c}\left(n^{r}\right)\right)$ and $V\left(z^{s}\right)=diag\;(V_{1}\left(z_{1}^{s}\right),\ldots ,V_{M}\left(z_{M}^{s}\right))$. To facilitate the presentation, a summary of the expressions for *A*_*m*_, *C*_*m*_, $A_{m}^{c}$, *D*_*m*_, $D_{m}^{c}$, and *V*_*m*_ can be found Section B.3.3 in S1 File. In **RP-MS**, the first constraint ensures that the total testing cost remains within the given budget. The second constraint, with *γ* denoting a sufficiently large number, ensures that when $x_{m}^{p}=1$, $x_{m}^{r}$ converges to zero, and when $x_{m}^{p}\geq 0$, this constraint becomes redundant. The purpose of this constraint is to prevent infinite alternative optimal solutions: For example, when $x_{m}^{p}=1$, indicating that category *m* is fully tested through proactive screening, there are no remaining subjects for reactive screening. In such cases, the value of $x_{m}^{r}$ can vary between 0 and 1 while still maintaining the same objective value. It is observed that the objective function is composed of four components: Missclassifications resulting from: (i) proactive screening (i.e., ***A***(*n*^*p*^)^⊤^***x***^*p*^); (ii) reactive screening (i.e., (***I*** − ***x***^*p*^)^⊤^***Q***(*n*^*r*^)***x***^*r*^); (iii) untested symptomatic cases (i.e., ${\left(I-x^{p}\right)}^{⊤}V\left(z^{s}\right)\left(I-x^{r}\right)$), which is governed by ***z***^*s*^; and (iv) untested non-symptomatic cases (i.e., ${\left(I-x^{p}\right)}^{⊤}C\left(z^{\bar{s}}\right)$), which is governed by $z^{\bar{s}}$. In the next section, we will analyze the specific structure of **RP-MS** to establish key properties which will then be utilized to construct an efficient globally convergent solution scheme.

## 3 Structural properties and solution scheme

In its current form, Problem **RP-MS** is a mixed-integer nonlinear programming problem, which is challenging to solve to global optimality [85]. The most straightforward approach is to utilize off-the-shelf solvers. However, due to the non-convex nature of the problem and the presence of discrete variables, commercial solvers may only converge to sub-optimal solutions that are far from global optimality, or in some cases, they may even fail to converge. Additionally, these solvers can experience issues such as stagnation, which are particularly common when degeneracy is present, as observed in **RP-MS**. Therefore, in what follows, we attempt to analyze Problem **RP-MS** and identify key structural properties which we use to construct an efficient globally optimal solution scheme for Problem **RP-MS**.

To initiate the analysis, we observe that variable ***z***, which represents decisions related to the classification policies, introduces higher-order nonlinear terms in the objective function. Consequently, the inclusion of ***z*** significantly increases the complexity. Hence, our first step is to demonstrate that the optimal classification policies for both untested symptomatic cases (i.e., $z_{m}^{s}$) and non-symptomatic cases (i.e., $z_{m}^{\bar{s}}$) can be predetermined for all categories m∈M. We summarize this result in the following lemma.

**Lemma 1**. *Let* $z_{m}^{*}=\left[z_{m}^{s^{*}},z_{m}^{{\bar{s}}^{*}}\right]$ *denote the optimal classification policies for category* m∈M. *Then, for a given* λ_*m*_, *the optimal classification policy is given by*:

$$z_{m}^{s^{*}}=\{\begin{array}{cc} 1, & if\;J_{m}\geq 1-\lambda_{m}\\ 0, & otherwise \end{array}and\;z_{m}^{{\bar{s}}^{*}}=\{\begin{array}{cc} 1, & if\;H_{m}\geq 1-\lambda_{m}\\ 0, & otherwise \end{array}.$$

Lemma 1 demonstrates that it is possible to determine the optimal classification policies beforehand for any category m∈M given λ_*m*_. Upon examining this lemma, we observe that the optimal classification policies for both symptomatic and non-symptomatic cases are always integer values, specifically 0 or 1. In other words, it is never optimal to have a combination of two classification policies for one category. This behavior is intuitive because we assume that subjects within a given category are identical, making it illogical to have different classification policies for the same subject. Next, we notice that the choice of a specific classification policy is determined by a single parameter, such as *J*_*m*_ and *H*_*m*_. This is expected since, for example, the classification policy for untested symptomatic individuals ($z_{m}^{s*}$) is dictated by *J*_*m*_, representing the risk associated with untested symptomatic subjects in category *m*. When λ_*m*_ = 0.5, which represents a scenario where both false negative and false positive errors are equally important in category *m*, the condition for classifying untested symptomatic cases as positive is *J*_*m*_ ≥ 0.5. This criterion is logical because if the risk associated with symptomatic cases exceeds 0.5, it becomes optimal to classify these untested symptomatic cases as positive. On the other hand, when λ_*m*_ = 1 (λ_*m*_ = 0) representing a scenario where the emphasis is only placed on false negatives (positives), the condition *J*_*m*_ ≥ 0 (*J*_*m*_ ≥ 1) indicates that it is always optimal to classify these untested cases as positive (negative) as no false negative (positive) errors would occur. A similar analysis can be applied to non-symptomatic cases by noting that *H*_*m*_ represents the risk of non-symptomatic cases.

The decision variable ***n*** = [*n*^*p*^, *n*^*r*^], representing the testing scheme for proactive and reactive screening, also amplifies the complexity of the problem. In particular, the introduction of these integer decision variables not only results in higher-order non-linear terms but also introduces discreteness into the problem, significantly increasing its complexity. However, since the upper bounds for group sizes *n*^*p*^ and *n*^*r*^ are typically small (e.g., in the context of Polymerase Chain Reaction (PCR) testing, the dilution effect becomes negligible when the group size is less than 10 [83, 84]), it becomes feasible to efficiently enumerate all possible combinations of *n*^*p*^ and *n*^*r*^ to determine the optimal group size. Therefore, we propose to first fix the group size. In conjunction with Lemma 1, this allows us to transform the problem into a continuous optimization problem. This transformation may enable us to leverage the extensive theory of continuous programming. Let **RP-MS**(***n***, z*) denote Problem **RP-MS** for a given set of group size ***n*** = [*n*^*p*^, *n*^*r*^] and the optimal classification policies z*. Solving a series of **RP-MS**(***n***, z*) problems is equivalent to finding the optimal solution for the original **RP-MS**. Therefore, in what follows, our objective is to develop an efficient solution scheme for **RP-MS**(***n***, z*).

Even for a fixed group size ***n*** and under the optimal classification policies z*, **RP-MS**(***n***, z*) is a quadratically constrained quadratic program (QCQP) which still poses significant challenges. Observe that **RP-MS**(***n***, z*) has two quadratic terms (***I*** − ***x***^*p*^)^⊤^***Q***(*n*^*p*^)***x***^*r*^ and ${\left(I-x^{p}\right)}^{⊤}V\left(z^{s}\right)\left(I-x^{r}\right)$ in the objective function. Similarly, the budget constraint also involves a quadratic term (***I*** − ***x***^*p*^)^⊤^***Q***^*c*^(*n*^*r*^)***x***^*r*^. Consequently, Problem **RP-MS**(*n*, z*) can be classified as a bilinear program due to the presence of bilinear functions in the objective function and the budget constraint. A bilinear function takes two variables and produces a result that is a linear combination of those variables. In other words, it’s a function that is linear in each of its variables separately but exhibits a multiplicative interaction between the variables. Therefore, Problem **RP-MS**(*n*, z*) belongs to a subclass of QCQP, which is known to be an NP-Hard problem [86]. The existing literature offers a plethora of approaches. For instance, there are methodologies specifically tailored for bilinear programs, such as cutting planes [87, 88] and McCormick relaxation [89, 90]. Additionally, there are well-established methodologies for solving QCQP using two main relaxations of QCQPs: Semidefinite programming (SDP) and the Reformulation-Linearization Technique (RLT) [91, 92]. Nonetheless, these approaches suffer from several computational limitations as the number of variables grows. For example, when dealing with large-scale QCQPs, the computing time required to solve SDP relaxations grows rapidly as the size of the problem expands. This rapid growth in computing time makes it impractical to obtain optimal values of large-scale QCQPs within a reasonable amount of time [93]. Similarly, the RLT approach necessitates a significant number of additional constraints and variables when applied to a large QCQP instance, rendering it computationally prohibitive [92]. Furthermore, in practice, the budget level could vary over time. In such cases, it may be necessary to solve Problem **RP-MS**(***n***, z*) repeatedly under different budget levels. These computational limitations are further amplified when enumerating different combinations of *n*^*p*^ and *n*^*r*^ to obtain the optimal solution of the original Problem **RP-MS**. Consequently, in what follows, we leverage the structure of Problem **RP-MS**(***n***, z*) to identify key properties which we use to construct an efficient custom solution scheme.

Before we delve into the detailed analysis, let us first simplify the notation in the objective function, while keeping all the constraints unchanged, as they do not involve classification policies. Once the optimal classification policies ***z*** are determined, the performance matrix ***V***(***z***^*s*^) and the vector $C(z^{\bar{s}})$ become constants. Therefore, to streamline the analysis process, we combine these two terms into ***A***(*n*) and ***Q***(*n*). Subsequently, the objective function of Problem **RP-MS**(***n***, z*) can be expressed as:

$$\begin{array}{c} \underset{x}{\text{minimize}}\;\tilde{A}{\left(n,z^{*}\right)}^{⊤}x^{p}+{\left(I-x^{p}\right)}^{⊤}\tilde{Q}\left(n,z^{*}\right)x^{r}, \end{array}$$

where $\tilde{A}\left(n,z^{*}\right)=\left[{\tilde{A}}_{1}\left(n,z^{*}\right),\ldots ,{\tilde{A}}_{M}\left(n,z^{*}\right)\right]$ and $\tilde{Q}\left(n,z^{*}\right)=diag\left({\tilde{D}}_{1}\left(n,z^{*}\right),\ldots ,{\tilde{D}}_{1}\left(n,z^{*}\right)\right)$. The detailed expressions of ${\tilde{A}}_{m}$ and ${\tilde{D}}_{m}$ can be found in Section B.3.4 in S1 File. We first identify a theoretical upper bound on the budget that an optimal solution will never exceed. This upper bound is formally presented in the following lemma.

**Lemma 2**. *For a given* **λ** = [λ_*m*_], *the optimal solution of **RP-MS***(***n***, z*) *will never utilize a budget greater than*:

$${\bar{B}}_{\lambda }\left(n,z^{*}\right)=\sum_{m\in M(n,z^{*},\lambda )}[A_{m}^{c}\left(n\right)\cdot t_{m}\left(n,z^{*}|\lambda_{m}\right)+D_{m}^{c}\left(n\right)\cdot \left(1-t_{m}\left(n,z^{*}|\lambda_{m}\right)\right)],$$

*where*

$$M\left(n,z^{*},\lambda \right)=\{m\in M:\text{min}\;\{{\tilde{A}}_{m}\left(n,z^{*}|\lambda_{m}\right),{\tilde{D}}_{m}\left(n,z^{*}|\lambda_{m}\right)\}<0\}\subseteq M,$$

*and t*_*m*_(***n***, z*|λ_*m*_) = 1 *if* ${\tilde{A}}_{m}\left(n,z^{*}|\lambda_{m}\right)<{\tilde{D}}_{m}\left(n,z^{*}|\lambda_{m}\right)$, 0 *otherwise for all* $m\in M(n,z^{*},\lambda )$.

Lemma 2 establishes a theoretical budget upper bound for Problem **RP-MS**(***n***, z*). By considering all possible combinations of group sizes ***n*** = [*n*^*r*^, *n*^*p*^] and utilizing Lemma 2, we can derive the budget upper bound for the original Problem **RP-MS**. This upper bound can be represented as ${\bar{B}}_{\lambda }={\text{max}}_{n\in N_{1}\times N_{2}}\;{\bar{B}}_{\lambda }\left(n,z^{*}\right)$ where $N^{p}=\left\{1,\cdots ,N^{p}\right\}$ and $N^{r}=\left\{1,\cdots ,N^{r}\right\}$. As such, any additional budget on top of ${\bar{B}}_{\lambda }$ will never be utilized in the optimal solution of **RP-MS**. Therefore, in the following analysis, we assume, without loss of generality, the given budget is less than or equal to ${\bar{B}}_{\lambda }$. Furthermore, observe that the budget is determined by the cost of the test design that achieves the best performance (i.e., lowest objective function coefficient) for each category. This is expected since when the budget is sufficient, it is optimal to utilize the best performing design regardless of its cost. As such, when the given budget $B\geq {\bar{B}}_{\lambda }\left(n,z\right)$, the optimal solution of **RP-MS**(*n*, ***z***) can be fully characterized, which we summarize in Corollary 1.

**Corollary 1**. For a given **λ** = [λ_*m*_], when $B\geq {\bar{B}}_{\lambda }\left(n,z^{*}\right)$, the optimal solution of **RP-MS**(***n***, z*) is given by:

$$\begin{array}{c} x^{*}=\{\begin{array}{cc} x_{m}^{p^{*}}=1,x_{m}^{r^{*}}=0,\; & \text{if}\;t_{m}\left(n,z|\lambda_{m}\right)=1,m\in M\left(n,z^{*},\lambda \right)\\ x_{m}^{p^{*}}=0,x_{m}^{r^{*}}=1,\; & \text{if}\;t_{m}\left(n,z|\lambda_{m}\right)=0,m\in M\left(n,z^{*},\lambda \right)\\ x_{m}^{p^{*}}=0,x_{m}^{r^{*}}=0,\; & m\notin M(n,z^{*},\lambda ), \end{array} \end{array}$$

where

$$M\left(n,z^{*},\lambda \right)=\{m\in M:\text{min}\;\{{\tilde{A}}_{m}\left(n,z^{*}|\lambda_{m}\right),{\tilde{D}}_{m}\left(n,z^{*}|\lambda_{m}\right)\}<0\}\subseteq M,$$

and *t*_*m*_(***n***, z*|λ_*m*_) = 1 if ${\tilde{A}}_{m}\left(n,z^{*}|\lambda_{m}\right)<{\tilde{D}}_{m}\left(n,z^{*}|\lambda_{m}\right)$, 0 otherwise for all $m\in M(n,z^{*},\lambda )$.

Corollary 1 highlights the unique structure of the optimal solution of **RP-MS**(***n***, z*) when the budget becomes sufficient, emphasizing the significant impact of the budget level on the optimal solution of Problem **RP-MS**(*n*, ***z***). The results in Corollary 1 also provide decision-makers with valuable insights regarding the most ideal screening strategy regardless of the testing budget. Subsequently, we provide a lower bound on the budget below which the optimal solution follows a special structure. This result is presented formally in Lemma 3.

**Lemma 3**. *For a given* **λ** = [λ_*m*_], *define* $t_{\underline{m}}\left(n,z|\lambda_{\underline{m}}\right)=1$ *if* $A_{\underline{m}}\left(n|\lambda_{m}\right)<D_{\underline{m}}\left(n,z|\lambda_{m}\right)$, 0 *otherwise. The lower bound on budget for Problem **RP-MS***(***n***, z*) *is defined as*:

$${\underline{B}}_{\lambda }\left(n,z^{*}\right)=A_{\underline{m}}^{c}\left(n|\lambda_{\underline{m}}\right)\cdot t_{\underline{m}}\left(n,z^{*}|\lambda_{\underline{m}}\right)+D_{\underline{m}}^{c}\left(n|\lambda_{\underline{m}}\right)\cdot [1-t_{\underline{m}}\left(n,z^{*}|\lambda_{\underline{m}}\right)],$$

*where* $\underline{m}$ *is given by*:

$$\underline{m}=\underset{m\in M(n,z^{*},\lambda )}{\text{arg}\;\text{min}}\;\{\frac{{\tilde{A}}_{m}\left(n,z^{*}|\lambda_{m}\right)}{A_{m}^{c}\left(n|\lambda_{m}\right)}\cdot t_{\underline{m}}\left(n,z^{*}|\lambda_{m}\right)+\frac{{\tilde{D}}_{m}\left(n,z^{*}|\lambda_{m}\right)}{D_{m}^{c}\left(n|\lambda_{m}\right)}\cdot [1-t_{\underline{m}}\left(n,z^{*}|\lambda_{m}\right)]\},$$

*and*

$$M\left(n,z^{*},\lambda \right)=\{m\in M:\text{min}\;\{{\tilde{A}}_{m}\left(n,z^{*}|\lambda_{m}\right),{\tilde{D}}_{m}\left(n,z^{*}|\lambda_{m}\right)\}<0\}\subseteq M.$$

*When* $B\leq {\underline{B}}_{\lambda }\left(n,z^{*}\right)$, *the optimal solution of **RP-MS***(***n***, z*) *is given by*:

$$\begin{array}{c} x^{*}=\{\begin{array}{cc} x_{m}^{p^{*}}=\frac{B}{A_{m}^{c}\left(n|\lambda_{m}\right)}\cdot t_{\underline{m}}\left(n,z^{*}|\lambda_{m}\right),x_{m}^{r^{*}}=\frac{B}{D_{m}^{c}\left(n|\lambda_{m}\right)}\cdot [1-t_{\underline{m}}\left(n,z^{*}|\lambda_{m}\right)],\; & if\;m=\underline{m}\\ x_{m}^{p^{*}}=x_{m}^{r^{*}}=0,\; & otherwise. \end{array} \end{array}$$

Lemma 3 presents a lower bound on the budget and characterizes the optimal solution when the budget falls below the lower bound ${\underline{B}}_{\lambda }\left(n,z^{*}\right)$. Specifically, Lemma 3 indicates that the variable with the lowest objective to the cost coefficient ratio (i.e., ${\tilde{A}}_{m}/A_{m}^{c}$ or ${\tilde{D}}_{m}/D_{m}^{c}$) among all categories will be selected first when the budget is extremely limited. Having identified two budget thresholds and their corresponding optimal solutions, we will now discuss a more general scenario where the given budget falls between these two thresholds. We initiate the analysis by identifying scenarios in which certain decision variables are guaranteed to be equal to zero. This result is formally provided in the following lemma.

**Lemma 4**. *For a given* **λ** = [λ_*m*_] *and any given budget B, the following two statements must hold for the optimal solution of **RP-MS***(***n***, z*) *for category* m∈M:

*(i) If* $\frac{{\tilde{A}}_{m}\left(n,z^{*}|\lambda_{m}\right)}{A_{m}^{c}\left(n|\lambda_{m}\right)}\leq \frac{{\tilde{D}}_{m}\left(n,z^{*}|\lambda_{m}\right)}{D_{m}^{c}\left(n|\lambda_{m}\right)}$ *and* ${\tilde{A}}_{m}\left(n,z^{*}|\lambda_{m}\right)\leq {\tilde{D}}_{m}\left(n,z^{*}|\lambda_{m}\right)$ , *then* $x_{m}^{r^{*}}=0$.
*(ii) If* $\frac{{\tilde{A}}_{m}\left(n,z^{*}|\lambda_{m}\right)}{A_{m}^{c}\left(n|\lambda_{m}\right)}>\frac{{\tilde{D}}_{m}\left(n,z^{*}|\lambda_{m}\right)}{D_{m}^{c}\left(n|\lambda_{m}\right)}$ *and* ${\tilde{A}}_{m}\left(n,z^{*}|\lambda_{m}\right)>{\tilde{D}}_{m}\left(n,z^{*}|\lambda_{m}\right)$ , *then* $x_{m}^{p^{*}}=0$.

Lemma 4 establishes that, within a specific category *m*, the decision variable possessing both the lowest objective-to-cost ratio and the lowest objective coefficient will dominate other testing alternatives. This property is particularly valuable as it can be utilized to identify all decision variables that are guaranteed to be zero beforehand. Therefore, this result can serve as a preprocessing step for the solution scheme, allowing for a substantial reduction in the number of decision variables and facilitating the solving process. For instance, in our case study, the preprocessing process eliminates around 30% of the decision variables beforehand, significantly reducing computational times for the numerical experiment. Building upon Lemma 4, we derive a fundamental structural property, which is summarized in Lemma 5.

**Lemma 5**. *For any given vector* **λ** = [λ_*m*_] *and budget B, the optimal solution of **RP-MS*** $\left(n,z^{*}\right)$ *can result in at most one category m having two distinct values at optimality (i.e.*, $x_{m}^{p*}>0$ *and* $x_{m}^{r*}>0$). *In addition, this scenario can only appear within category* $m\in F_{1}\left(n^{*},z,\lambda \right)\cup F_{2}\left(n^{*},z,\lambda \right)$, *where* $F_{1}\left(n^{*},z,\lambda \right)$ *and* $F_{2}\left(n^{*},z,\lambda \right)$ *are defined as*:

$$F_{1}\left(n^{*},z,\lambda \right)=\{m\in M:\frac{{\tilde{A}}_{m}\left(n,z^{*}|\lambda_{m}\right)}{A_{m}^{c}\left(n|\lambda_{m}\right)}\leq \frac{{\tilde{D}}_{m}\left(n,z^{*}|\lambda_{m}\right)}{D_{m}^{c}\left(n|\lambda_{m}\right)},0>{\tilde{A}}_{m}\left(n|\lambda_{m}\right)>{\tilde{D}}_{m}\left(n,z^{*}|\lambda_{m}\right)\},$$

*and*

$$F_{2}\left(n^{*},z,\lambda \right)=\{m\in M:\frac{{\tilde{A}}_{m}\left(n,z^{*}|\lambda_{m}\right)}{A_{m}^{c}\left(n|\lambda_{m}\right)}\geq \frac{{\tilde{D}}_{m}\left(n,z^{*}|\lambda_{m}\right)}{D_{m}^{c}\left(n|\lambda_{m}\right)},A_{m}\left(n,z|\lambda_{m}\right)<D_{m}\left(n,z^{*}|\lambda_{m}\right)<0\}.$$

Lemma 5 demonstrates that the optimal solution of Problem **RP-MS**(***n***, ***z***) can have at most one category with two non-zero values. This behavior indicates that all categories, except for at most one category, will employ only one testing design, which enhances the practicality of the optimal solution. Based on this insight, we take advantage of the special property of Problem **RP-MS**(***n***, z*) and the results in Lemma 5 to develop an efficient solution scheme for Problem **RP-MS**(***n***, z*). This solution scheme is formally presented in the following theorem. To facilitate the presentation, we drop the term (***n***, z*) from all the coefficients.

**Theorem 1**. *For a given* **λ** = [λ_*m*_] *and budget B, the optimal solution of **RP-MS***(***n***, z*) *will sequentially select the category with the most negative ratio from the vector* ***v*** = [*v*_1_, …, *v*_*M*_]. *The vector* ***v*** *is initially defined as*:

$$v=[\text{min}\;\{\frac{{\tilde{A}}_{1}}{A_{1}^{c}},\frac{{\tilde{D}}_{1}}{D_{1}^{c}},0\},\ldots ,\text{min}\;\{\frac{{\tilde{A}}_{M}}{A_{M}^{c}},\frac{{\tilde{D}}_{M}}{D_{M}^{c}},0\}].$$

*Let* $\tilde{m}$ *denote the selected category, where* $\tilde{m}={\text{arg}\;\text{min}}_{m}\;\left\{v_{m}\right\}$. *The optimal solution and value of* $v_{\tilde{m}}$ *will be updated as follows*:

*(i) If* $v_{\tilde{m}}={\tilde{A}}_{\tilde{m}}/A_{\tilde{m}}^{c}$ , *then set* $x_{m}^{p^{*}}=\text{min}\;\left\{1,B/A_{\tilde{m}}^{c}\right\}$ *and* $B=B-A_{\tilde{m}}^{c}x_{m}^{p^{*}}$. *If* ${\tilde{A}}_{\tilde{m}}\leq {\tilde{D}}_{\tilde{m}}$, *then update* $v_{\tilde{m}}=0$, *otherwise update* $v_{\tilde{m}}=\left(A_{\tilde{m}}-D_{\tilde{m}}\right)/\left(A_{\tilde{m}}^{c}-D_{\tilde{m}}^{c}\right)$.
*(ii) If* $v_{\tilde{m}}={\tilde{D}}_{\tilde{m}}/D_{\tilde{m}}^{c}$ , *then set* $x_{m}^{r^{*}}=\text{min}\;\left\{1,B/D_{\tilde{m}}^{c}\right\}$ *and* $B=B-D_{\tilde{m}}^{c}x_{m}^{r^{*}}$. *If* ${\tilde{D}}_{\tilde{m}}\leq {\tilde{A}}_{\tilde{m}}$, *then update* $v_{\tilde{m}}=0$, *otherwise update* $v_{\tilde{m}}=\left(A_{\tilde{m}}-D_{\tilde{m}}\right)/\left(A_{\tilde{m}}^{c}-D_{\tilde{m}}^{c}\right)$.
*(iii) If* $v_{\tilde{m}}=\left({\tilde{A}}_{\tilde{m}}-{\tilde{D}}_{\tilde{m}}\right)/\left(A_{\tilde{m}}^{c}-D_{\tilde{m}}^{c}\right)$ *and* ${\tilde{A}}_{\tilde{m}}>{\tilde{D}}_{\tilde{m}}$ , *then set* $x_{m}^{r^{*}}=1$, $x_{m}^{p^{*}}=1-\text{min}\;\left\{1,B/\left(D_{\tilde{m}}^{c}-A_{\tilde{m}}^{c}\right)\right\}$, $B=B-\left(D_{\tilde{m}}^{c}-A_{\tilde{m}}^{c}\right)x_{m}^{p^{*}}$, *and update* $v_{\tilde{m}}=0$.
*(iv) If* $v_{\tilde{m}}=\left({\tilde{A}}_{\tilde{m}}-{\tilde{D}}_{\tilde{m}}\right)/\left(A_{\tilde{m}}^{c}-D_{\tilde{m}}^{c}\right)$ *and* ${\tilde{A}}_{\tilde{m}}<{\tilde{D}}_{\tilde{m}}$ , *then set* $x_{m}^{p^{*}}=\text{min}\;\left\{1,B/\left(A_{\tilde{m}}^{c}-D_{\tilde{m}}^{c}\right)\right\}$, $B=B-\left(A_{\tilde{m}}^{c}-D_{\tilde{m}}^{c}\right)x_{m}^{p^{*}}$, *and update* $v_{\tilde{m}}=0$.

*The procedure repeats until either the budget is exhausted or* $v_{\tilde{m}}\geq 0$ .

Theorem 1 presents an efficient solution scheme for **RP-MS**(***n***, z*). The solution scheme utilizes a dynamic greedy policy by taking advantage of its knapsack-style structure. The vector ***v*** represents the optimal ratio of the objective function to the cost coefficient for each category. It serves as a dynamic parameter that gets updated as the algorithm iterates. The underlying concept is to identify the most negative ratio across all categories, which indicates the best testing option for a particular category. Subsequently, the algorithm adjusts the performance ratio and current budget level accordingly. This process continues until either the objective function cannot be further improved through additional testing (i.e., $v_{\tilde{m}}\geq 0$), or the budget is fully utilized. Once the optimal solution for Problem **RP-MS**(***n***, z*) has been identified, we can proceed to enumerate all possible combinations of ***n*** in order to determine their respective optimal solutions. By doing so, we can identify the optimal group size n*. Next, we conclude this section by providing a consequent result from Theorem 1.

**Corollary 2**. For a given **λ** = [λ_*m*_] and budget *B*, the following two statements must hold for the optimal solution of **RP-MS**(***n***, z*):

(i) If $\frac{A_{m}\left(n,z^{*}|\lambda_{m}\right)}{A_{m}^{c}\left(n|\lambda_{m}\right)}\leq \frac{D_{m}\left(n,z^{*}|\lambda_{m}\right)}{D_{m}^{c}\left(n|\lambda_{m}\right)}$ and *A*_*m*_(*n*, z*|λ_*m*_) > *D*_*m*_(*n*, z*|λ_*m*_), $x_{m}^{r^{*}}$ must be binary, i.e., $x_{m}^{r^{*}}\in \left\{0,1\right\}$.
(ii) If $\frac{A_{m}\left(n,z^{*}|\lambda_{m}\right)}{A_{m}^{c}\left(n|\lambda_{m}\right)}\geq \frac{D_{m}\left(n,z^{*}|\lambda_{m}\right)}{D_{m}^{c}\left(n|\lambda_{m}\right)}$ and *A*_*m*_(*n*, z*|λ_*m*_) < *D*_*m*_(*n*, z*|λ_*m*_), $x_{m}^{p^{*}}>0$ if and only if $x_{m}^{r^{*}}=1$.

Corollary 2 reveals some interesting behaviors of the optimal solution. First, Corollary 2(i) indicates that when proactive testing for category *m* is more cost-effective, but reactive testing yields better performance at a higher expense, the remaining subjects not tested proactively will either remain untested entirely or undergo reactive testing altogether. Second, Corollary 2(ii) states that if, in contrast, reactive testing for category *m* is more cost-effective, while proactive testing has a better performing coefficient but more expensive, proactive testing will only be considered if the untested subjects are being tested. This behavior is intuitive, as the testing design with the superior cost-performance ratio is given priority initially. Subsequently, the solution scheme transitions to the testing option that offers better performance, despite being more expensive.

### 3.1 Model extension

In this section, we aim to extend **RP-MS** to a more general setting. In **RP-MS**, we implement a uniform testing scheme for reactive and proactive screening across all categories (i.e.,$n_{1}^{p}=n_{2}^{p}=\cdots =n_{M}^{p}=n^{p}$ and $n_{1}^{r}=n_{2}^{r}=\cdots =n_{M}^{r}=n^{r}$). However, given the existing heterogeneity across categories, it would be more realistic and beneficial to allow each category to have its own tailored testing scheme. Introducing more customization might introduce challenges in executing the screening strategy, but it also holds the potential for further improvements in reducing misclassifications. Therefore, in the following discussion, we attempt to generalize **RP-MS** to incorporate different $n_{m}^{p}$ and $n_{m}^{r}$ for each category m∈M.

By considering different testing schemes ($n^{p}={\left[n_{m}^{p}\right]}_{m\in M}$ and $n^{r}={\left[n_{m}^{r}\right]}_{m\in M}$) into **RP-MS**, the generalized **RP-MS** can be written as:

$$\begin{array}{cc} \underset{x,z,n^{p},n^{r}}{\text{minimize}}\; & A{\left(n^{p}\right)}^{⊤}x^{p}+{\left(I-x^{p}\right)}^{⊤}Q\left(n^{r}\right)x^{r}+{\left(I-x^{p}\right)}^{⊤}V\left(z^{s}\right)\left(I-x^{r}\right)+{\left(I-x^{p}\right)}^{⊤}C\left(z^{\bar{s}}\right)\\ \text{subject}\;\text{to}\; & A^{c}{\left(n^{p}\right)}^{⊤}x^{p}+{\left(I-x^{p}\right)}^{⊤}Q^{c}\left(n^{r}\right)x^{r}\leq B\\ & \gamma \left(1-x_{m}^{p}\right)\geq x_{m}^{r},\;\forall \;m\in M\\ & x_{m}^{p},x_{m}^{r},z_{m}^{s},z_{m}^{\bar{s}}\in \left[0,1\right],\;\forall \;m\in M\\ & n_{m}^{p}\in \left\{1,\ldots ,N^{p}\right\},n_{m}^{r}\in \left\{1,\ldots ,N^{r}\right\}. \end{array}$$

The new optimization method retains the main structure of **RP-MS**, but introduces a modification to the coefficient vectors/matrices (e.g., ***A***(⋅) and ***Q***(⋅)). Specifically, instead of being determined by a single decision variable (i.e., *n*^*p*^ or *n*^*r*^) in **RP-MS**, the coefficient vectors/matrices are now determined by a vector of decision variables (***n***^*p*^ or ***n***^*r*^). This change will have an impact on the previously established solution approach. In the previous approach, we solve the problem by first fixing group sizes *n*^*p*^ and *n*^*r*^, and then solve a series of sub-problems of **RP-MS** for different combinations of *n*^*p*^ and *n*^*r*^. This approach was feasible due to the limited number of two-dimensional combinations of *n*^*p*^ and *n*^*r*^ within the imposed upper bounds of *N*^*p*^ and *N*^*r*^. However, with the incorporation of different schemes across categories, the domain of $n^{p}=\left[n_{m}^{p}\right]$ and $n^{r}=\left[n_{m}^{r}\right]$ expands exponentially as the number of categories increases. For example, in our case study, we consider over 3,000 categories. Assuming a constant upper bound of *N*^*p*^ = *N*^*r*^ = 10, there are 10^2⋅3000^ scenarios to consider. Consequently, exhaustively exploring all possible combinations becomes impractical for large instances. In what follows, we reformulate the problem in a more tractable manner by introducing new decision variables.

Let $u_{m}^{n^{p}}$ and $v_{m}^{n^{r}}\in \left[0,1\right]$ represent the proportion of subjects in category m∈M that are proactively tested with group size *n*^*p*^ and reactively tested with group size *n*^*r*^. Let $u=[u_{m}^{n^{p}}]$ and $v=[v_{m}^{n^{r}}]$ represent the vector form of the decision variables. Using the new defined decision variables, we present the new optimization model, which we refer to as **Customized Reactive and Proactive Screening (CRP-MS)** problem:

$$\begin{array}{cc} \underset{u,v,z}{\text{minimize}}\; & \sum_{m=1}[\sum_{n^{p}=1}^{N_{m}^{p}}A_{m}\left(n^{p}\right)u_{m}^{n^{p}}+\sum_{n^{r}=1}^{N_{m}^{r}}D_{m}\left(n^{r}\right)\left(1-\sum_{n^{p}=1}^{N_{m}^{p}}u_{m}^{n^{p}}\right)v_{m}^{n^{r}}\\ & \;\;\;+V\left(z_{m}^{s}\right)\left(1-\sum_{n^{p}=1}^{N_{m}^{p}}u_{m}^{n^{p}}\right)\left(1-\sum_{n^{r}=1}^{N_{m}^{r}}v_{m}^{n^{r}}\right)+C\left(z_{m}^{\bar{s}}\right)\left(1-\sum_{n^{r}=1}^{N_{m}^{r}}v_{m}^{n^{r}}\right)]\\ \text{subject}\;\text{to}\; & \sum_{m=1}[\sum_{n^{p}=1}^{N_{m}^{p}}A_{m}^{c}\left(n^{p}\right)u_{m}^{n^{p}}+\sum_{n^{r}=1}^{N_{m}^{r}}D_{m}^{c}\left(n^{r}\right)\left(1-\sum_{n^{p}=1}^{N_{m}^{p}}u_{m}^{n^{p}}\right)v_{m}^{n^{r}}]\leq B\\ & \gamma \left(1-\sum_{n^{p}=1}^{N_{m}^{p}}u_{m}^{n^{p}}\right)\geq \sum_{n^{r}=1}^{N_{m}^{r}}v_{m}^{n^{r}},\forall \;m\in M\\ & \sum_{n^{p}=1}^{N_{m}^{p}}u_{m}^{n^{p}}\leq 1,\forall \;m\in M\\ & \sum_{n^{r}=1}^{N_{m}^{r}}v_{m}^{n^{r}}\leq 1,\forall \;m\in M\\ & u_{m}^{n^{p}},v_{m}^{n^{r}}\in \left[0,1\right],\;\forall \;n^{p}\in \left\{1,\ldots ,N_{m}^{p}\right\},n^{r}\in \left\{1,\ldots ,N_{m}^{r}\right\},m\in M\\ & z_{m}^{s},z_{m}^{\bar{s}}\in \left[0,1\right],\;\forall \;m\in M. \end{array}$$ (CRP-MS)

**CRP-MS** exhibits a similar structure to **RP-MS**, while incorporating the addition of two new constraints: The third (forth) constraint set guarantees that the total testing proportion for each category through proactive (reactive) screening is less than or equal to one. Although introducing these decision variables increases the number of variables, the advantages of this reformulation method are multi-fold: First, it eliminates the discreteness associated with group sizes. While there still remains discreteness due to the decision variable ***z***, we will demonstrate later that the optimal solution for ***z*** can be determined beforehand, ultimately transforming **CRP-MS** into a continuous problem. Second, it reduces the order of the nonlinear term from cubic to quadratic, resulting in a similar structure to **RP-MS**(***n***, z*), for which a solution scheme has been established. In what follows, we first show that the optimal classification policies z* can be identified beforehand.

Lemma 1, which identifies the optimal classification policies for **RP-MS**(***n***, z*), remains valid for **CRP-MS**. This is because the optimal classification policy is independent of the screening strategy, and modifying the decision variables related to the screening strategy does not impact the classification policy. Consequently, Lemma 1 allows us to determine the optimal classification policy z* in advance. As such, in the subsequent analysis, our objective is to examine **CRP-MS**(z*), which represents **CRP-MS** for the given optimal classification policies z*. Subsequently, the objective of **CRP-MS**(z*) can be expressed as follows:

$$\begin{array}{c} \underset{u,v}{\text{minimize}}\;\sum_{m=1}[\sum_{n^{p}=1}^{N_{m}^{p}}{\tilde{A}}_{m}\left(n^{p}\right)u_{m}^{n^{p}}+\sum_{n^{r}=1}^{N_{m}^{r}}{\tilde{D}}_{m}\left(n^{r}\right)\left(1-\sum_{n^{p}=1}^{N_{m}^{p}}u_{m}^{n^{p}}\right)v_{m}^{n^{r}}]. \end{array}$$

All the constraints remain the same since they are not influenced by the decision of classification policies. The resulting problem now becomes a QCQP problem, which shares a similar structure with **RP-MS**(***n***, z*). However, recall that there are two additional constraints introduced in **CRP-MS**(z*) compared to **RP-MS**(***n***, z*), which ensure that the total test proportion from different testing schemes for each category is less than or equal to one. The inclusion of these additional constraints may require modifications in the previously established results and render the solution scheme for **RP-MS**(***n***, z*) invalid. Therefore, in the following analysis, we will examine the structural properties of **RP-MS**(***n***, z*) and determine if the results still hold or if any modifications/assumptions need to be imposed to maintain validity. We will then explore the possibility of generalizing the solution scheme of **RP-MS**(***n***, z*) to solve **CRP-MS**(z*).

To start with, we extend Lemma 2 and Lemma 3 to provide lower and upper budget bounds for **CRP-MS**(z*) by incorporating slight modifications that take into account customized testing schemes across counties. Then, Lemma 4, which presents a preprocessing procedure used to efficiently eliminate decision variables, can be extended to accommodate **CRP-MS**(z*) as well. As a result, we introduce the modified versions of Lemma 2 and 3 in Section D in S1 File. However, the key structural properties presented in Lemma 5 and the solution scheme presented in Theorem 1 cannot be easily extended to the generalized model due to the complexity of the new formulation, particularly the inclusion of additional constraint sets aimed at ensuring the overall test proportion is less than or equal to one.

While the solution scheme for **RP-MS**(***n***, z*) cannot be directly simplified and applied to **CRP-MS**(z*), Theorem 1 serves as a solid foundation and enhances our understanding of **CRP-MS**. Building upon Theorem 1, we propose an efficient heuristic solution scheme for **CRP-MS**, formally presented in Algorithm 1 (see Section E in S1 File. The core idea of Algorithm 1 is to initially solve a relaxation of **CRP-MS**(z*) without the set of test proportion constraints. This relaxation leads **CRP-MS**(z*) to have a similar structure to **RP-MS**(***n***, z*), for which we have developed a solution scheme. Subsequently, the algorithm modifies the obtained solution that violates the removed constraints. In particular, Algorithm 1 incorporates two layers of modification to ensure the obtained solution from the relaxation adheres to the feasible domain of the original **CRP-MS**, while striving to maintain the objective value as much as possible. The first layer of modification examines all categories and ensures that the total test proportion via proactive/reactive screening does not exceed one within each category. The algorithm then proceeds to the second layer, where it determines the best performing testing design for each category. Iterations continue until the obtained solution falls within the feasible domain of **CRP-MS**. We later examine the accuracy and efficiency of the proposed heuristic solution scheme by a comparative analysis with state-of-the-art solvers, as outlined in Section 4. The numerical results obtained from this comparison demonstrate that the heuristic solution scheme exhibits a substantial reduction in computational time while consistently delivering high-quality solutions comparable to those obtained from commercial solvers. As a result, it offers a more efficient approach to solving the problem at hand.

## 4 Numerical results: COVID-19 screening in the united states

In this section, our objective is to demonstrate the usefulness of our proposed methodology via a case study on COVID-19 screening in the US. The COVID-19 pandemic has caused many deaths and prolonged global economic disruptions. However, the research has indicated that the virus is expected to persist and circulate within society in the foreseeable future due to the emergence of new variants [95]. Therefore, it remains crucial for society to proactively equip itself to effectively manage and mitigate the ongoing impact of the virus, while simultaneously enhancing preparedness for future pandemic threats. On the other hand, there exists other respiratory diseases that cause a host of similar symptoms as COVID-19. For example, the most well-known and similar infectious disease is influenza (flu) and distinguishing between COVID-19 and the flu can be challenging as both diseases share similar symptoms such as coughing and fever. Hence, in this case study we consider a setting in which subjects exhibiting symptoms are caused by COVID-19 and/or the flu. Compared to the flu, COVID-19 has a significantly higher estimated *R*_0_ value, which represents the basic reproduction number. This measure is used to estimate the average number of new infections that can be generated by a single infected individual in a susceptible population. For COVID-19, The Omicron variant of COVID-19 has an estimated *R*_0_ value that falls within the range of 5.5 to 24 [96]. In contrast, the *R*_0_ value for the flu is estimated to be between 1 and 2 [97], signifying that COVID-19 is more infectious and poses greater challenges in terms of containment when compared to the flu. Additionally, COVID-19 is more likely to cause long-term health implications than the flu [98]. Therefore, implementing effective screening strategies to identify COVID-19 cases is critical.

**Fig 1:**
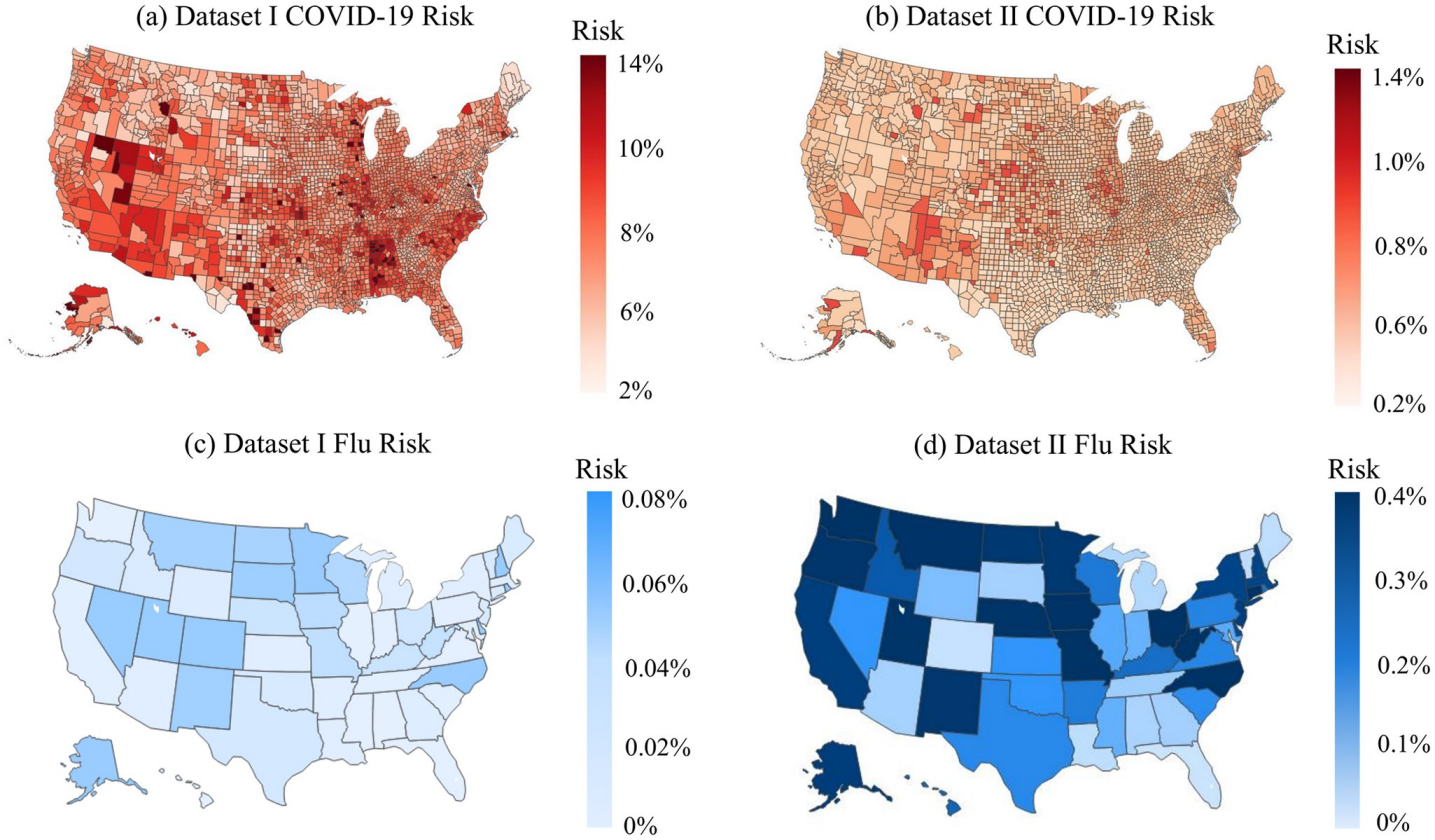
Risk of COVID-19 (county-level) and flu (state-level) in the US for dataset I (01/15/2022—01/22/2022) and II (11/28/2022–12/05/2022) [generated by Plotly in Python [94]].

Due to the evolving nature of COVID-19 (e.g., the emergence of new variants), the overall risk level fluctuates over time. For example, when a new variant emerges, there is typically a surge in COVID-19 cases, leading to heightened levels of risk. On the other hand, flu activity follows a seasonal pattern, exhibiting variations throughout different time periods. Consequently, diverse and tailored screening strategies are necessary to effectively address these distinct scenarios, and comparing screening strategies for different scenarios can provide additional valuable managerial insights. Therefore, this case study examines and explores two datasets corresponding to two distinct scenarios. The first dataset, referred to as dataset I, spans from 01/15/2022 to 01/22/2022, capturing a scenario characterized by a high risk of COVID-19 and a low risk of flu. In contrast, the second dataset, dataset II, encompasses the period from 11/28/2022 to 12/05/2022, representing a scenario where the risk of COVID-19 is low while the risk of flu is high. The impact of COVID-19 and the flu, however, is not evenly distributed across the population. For instance, Fig 1a and 1b illustrate the varying risk of both diseases across different counties during two time periods, while Fig 1c and 1d capture the risk of the flu across different states (due to data limitations, county-level data is not available for the flu). These figures reveal significant heterogeneity across different geographic regions. Analyzing geographic-based data can also offer valuable managerial insights, such as testing scheme distribution and budget allocation. To this end, we employ the geographic-based dataset for the case study, where each county represents a category, resulting in a total of 3, 143 risk categories in the US.

To assess the risk of COVID-19 and flu in each county, we utilize publicly available data from Coronavirus Resource Center at John Hopkins University of Medicine for COVID-19 [99], Centers for Disease Control and Prevention (CDC) for flu [100], census data from US Census Bureau [101]. In order to obtain the number of active positive cases in each category, we aggregate the newly reported positive cases over a period of seven days. This seven-day period is selected because the CDC quarantine policy for the latest Omicron variant (as of 02/03/2023) of COVID-19 recommends a seven-day quarantine period [102]. On the other hand, the flu is typically contagious from six to eight days, leading to an average contagious period of seven days [103]. Therefore, we consider infected cases for both COVID-19 and flu to remain infectious for 7 days. However, due to under-reporting issues, this procedure of aggregation is still limited in accurately estimating the active cases, as demonstrated by a number of studies. [104–106]. For example, [104] have shown that the vast majority of COVID-19 cases (approximately 75%) are unreported. To overcome this limitation for COVID-19, we multiplied the total number of confirmed cases in each category by a factor of four. Similarly, several research studies have utilized a multiplier approach to account for under-reporting of flu cases [107–109]. For instance, [108] has demonstrated that, among 1.8 million to 5.7 million cases, only 40 thousands of cases are reported at the early influenza in the US, resulting in an average under-reporting rate of 98.9%. Consequently, to account for the significant under-reporting of flu cases, we applied a multiplier of 94 to the number of confirmed cases of flu. Due to its seasonal nature and long history of circulation in society, it is expected that the flu has a higher underreporting rate compared to COVID-19. After calibration, the estimated overall average rates of COVID-19 (flu) for datasets I and II are 6.0% (0.03%) and 0.5%(0.17%) respectively. To provide a clearer distinction between these two datasets, we present the risk heatmaps of COVID-19 and flu for dataset I and dataset II in Fig 1. From the visualization, it is evident that dataset I exhibits a considerably higher COVID-19 risk while dataset II presents a higher flu risk.

Another set of important parameters includes the symptomatic rate of COVID-19 (i.e., the probability of individuals infected with COVID-19 displaying symptoms) and flu (i.e., the probability of individuals infected with flu exhibiting symptoms). According to the current studies, the overall symptomatic rate of COVID-19 is approximately 55% to 60% [110]. As such, we take the average and set the overall symptomatic rate of COVID-19 to be 57.5%. The simplest and most straightforward approach is the one-size-fits-all method, which applies the overall symptomatic rate to all counties uniformly. However, studies have found the evidence of increased symptomatic rate with disease(s)/condition(s) compared to cases with no underlying medical conditions [111, 112]. It is intuitive that individuals with poor health conditions have higher likelihood of developing symptoms, as they tend to be more susceptible to illness. To account for variations in health conditions and obtain a more precise estimate of the symptomatic rate for each county, we leverage a geographically-based dataset provided by the Centers for Disease Control and Prevention (CDC) [113]. This dataset encompasses the county-level prevalence of specific underlying conditions associated with COVID-19 illness, such as chronic diseases, diabetes, and heart diseases. By incorporating this valuable information alongside an overall symptomatic rate of 64.9%, we can evaluate the symptomatic rate specific to each county. The detailed calibration process is outlined in Section F in S1 File. With respect to the symptomatic rate of flu, the estimated prevalence of asymptomatic cases for any type of flu (such as influenza A, influenza B, etc.) is approximately 20% [114]. Individuals with underlying medical conditions like heart disease and chronic illnesses may indeed face a heightened rate of hospitalization and mortality. However, there is limited evidence indicating a substantially higher symptomatic rate for these individuals. This is especially true considering that the overall symptomatic rate of the flu is already significantly high across the general population. Therefore, we utilize 80% symptomatic rate for flu across all categories in this case study.

With respect to testing assays, there are two primary diagnostic tests for COVID-19 commonly used in practice: (i) the Reverse Transcription Quantitative Polymerase Chain Reaction (RT-qPCR), and (ii) the Rapid Antigen Test (RAT). The RT-qPCR test has been the gold standard for diagnosing COVID-19 due to its high accuracy. However, its use is limited by its high cost and the time required to complete the test. In light of these limitations, the Rapid Antigen Test (RAT) has become increasingly popular in practice due to its fast turnaround time, affordability, and acceptable accuracy. According to the broad scope of studies in the literature, the test sensitivity of RT-qPCR and RAT ranges from 0.9 to 0.99 [115–117]. As such, in this case study, we calculate the average and set the sensitivity to 0.95. On the other hand, the specificity of RT-qPCR and RAT is more consistent and ranges from 0.95 to 1, and thus is set to 0.97 [116, 118]. Lastly, to avoid the potential negative impact of the dilution effect (see Section 2), we set both upper bounds of the group size *N*^*p*^ and *N*^*r*^ to 10. This is because studies have shown that the dilution effect becomes negligible when group sizes are less than 10 [83, 84].

To define the user-defined parameters **λ** = [λ_*m*_], which represent the relative importance of false negatives versus false positives, we set its value to the ratio of the cost of a false negative to the total cost of misclassification. This can be calculated using the following formula:

$$\begin{array}{c} \lambda =\frac{w_{FN}}{w_{FN}+w_{FP}}, \end{array}$$

where *w*_*FN*_ and *w*_*FP*_ represent the cost of false negatives and false positives, respectively. However, there is no universally agreed method for evaluating the cost of false negatives and false positives, as the evaluation process can be influenced by various factors such as data availability and problem settings. With the main objective of demonstrating our model, we propose using the average cost of hospitalizations as an estimate for the cost of false negatives in our case study. This is because false negatives can increase disease transmission by leaving infected individuals unaware of their infectious status, which can result in a higher hospitalization rate. In addition, hospitalization rates are important indicators widely used by public health administrators to gain insight into disease progression and burden. The cost of hospitalization for COVID-19 treatment can vary widely, ranging from 31, 339$ to 111, 213$ [119], due to various factors. Firstly, the severity of illness plays a crucial role; for example, severe COVID-19 cases often require complex and costly treatments. Hospital resources and capacity also affect costs, especially in areas with high demand due to COVID-19 outbreaks, where strained resources can lead to higher charges. Hospitals with advanced facilities or specialized services may also charge more. Additionally, the length of a patient’s stay, influenced by disease severity, treatment response, and the need for intensive care, can increase costs. Lastly, location and the healthcare system’s structure mean costs can differ greatly between states. Based on this range, the average cost of hospitalization for COVID-19 treatment is set to be 71, 276$. However, not all false negatives will result in hospitalization as only individuals displaying symptoms are likely to be hospitalized. To account for this, we will utilize the average state-level hospitalization rates from CDC [120] during the periods of 01/15/2022 to 01/22/2022 and 11/28/2022 to 12/05/2022. Through these rates, we estimate the proportion of false negatives that are likely to result in hospitalization for each state. As a result, counties from different states will have different λ values due to varying hospitalization rates while counties within the same state will share a common λ. For example, the estimated hospitalization rates in Texas are 0.79% and 0.11% for the two time periods respectively (recall that dataset II has a lower COVID-19 prevalence rate). Using these rates, we can estimate the cost of a false negative to be 565.77$ and 78.41$ in Texas for datasets I and II, respectively.

On the other other hand, false positives may require additional confirmatory tests. The costs of COVID-19 testing assays vary depending on the type of testing conducted (e.g., PCR and RAT). Each type of test incurs different costs, which can vary based on the supplies required, demand, and accuracy levels. Consequently, we provide a price range for the various testing assays offered by the testing facilities and laboratories. The reported ranges span from $10 to $90 per test [121, 122]. Therefore, we set an average cost of 50$ per assay. Using the estimated costs of false negatives and false positives, we can calculate different λ values for each state. For instance, in the state of Texas, λ was found to be 565.77$/(565.77$ + 50$) ≈ 0.92 and 78.41$/(78.41$ + 50$) ≈ 0.61 for the two time periods, respectively. As observed, a lower hospitalization rate (dataset II) leads to a decreased λ value, indicating that false negatives become less significant. This can occur when the disease has a reduced likelihood of causing severe symptoms, which may be attributed to factors such as the emergence of new variants or the attainment of herd immunity. Therefore, the cost of false negatives can be adjusted according to the current hospitalization rate to reflect the changing severity of the disease. After calculation, the average values of **λ** across all states are 0.91 and 0.69 for datasets I and II respectively.

### 4.1 Performance comparisons

In our first experiment, we compare the performance of the proposed screening strategy with that of conventional screening strategies. According to the guidelines provided by the World Health Organization (WHO) [123], in the early stages of the COVID-19 pandemic, testing was largely focused on individuals displaying symptoms of the disease [124]. As testing resources became more available, the strategy shifted towards testing individuals with higher risks. Therefore, we consider a conventional screening strategy, which involves two stages: (i) when the budget is low (e.g., the early stage of the pandemic), the strategy relies on reactive screening and saves tests for individuals showing symptoms, which fails to target sub-populations (i.e., symptomatic subjects are randomly tested); (ii) once all symptomatic cases have been tested and there is excess budget, the strategy then prioritizes testing individuals in higher-risk categories. For testing schemes, the conventional strategy only involves individual testing and does not incorporate group testing, which significantly limits the effectiveness of the screening efforts. We believe that the conventional strategy we have considered closely resembles the real-world testing strategy, even though it may perfectly align with what was implemented.

**Fig 2:**
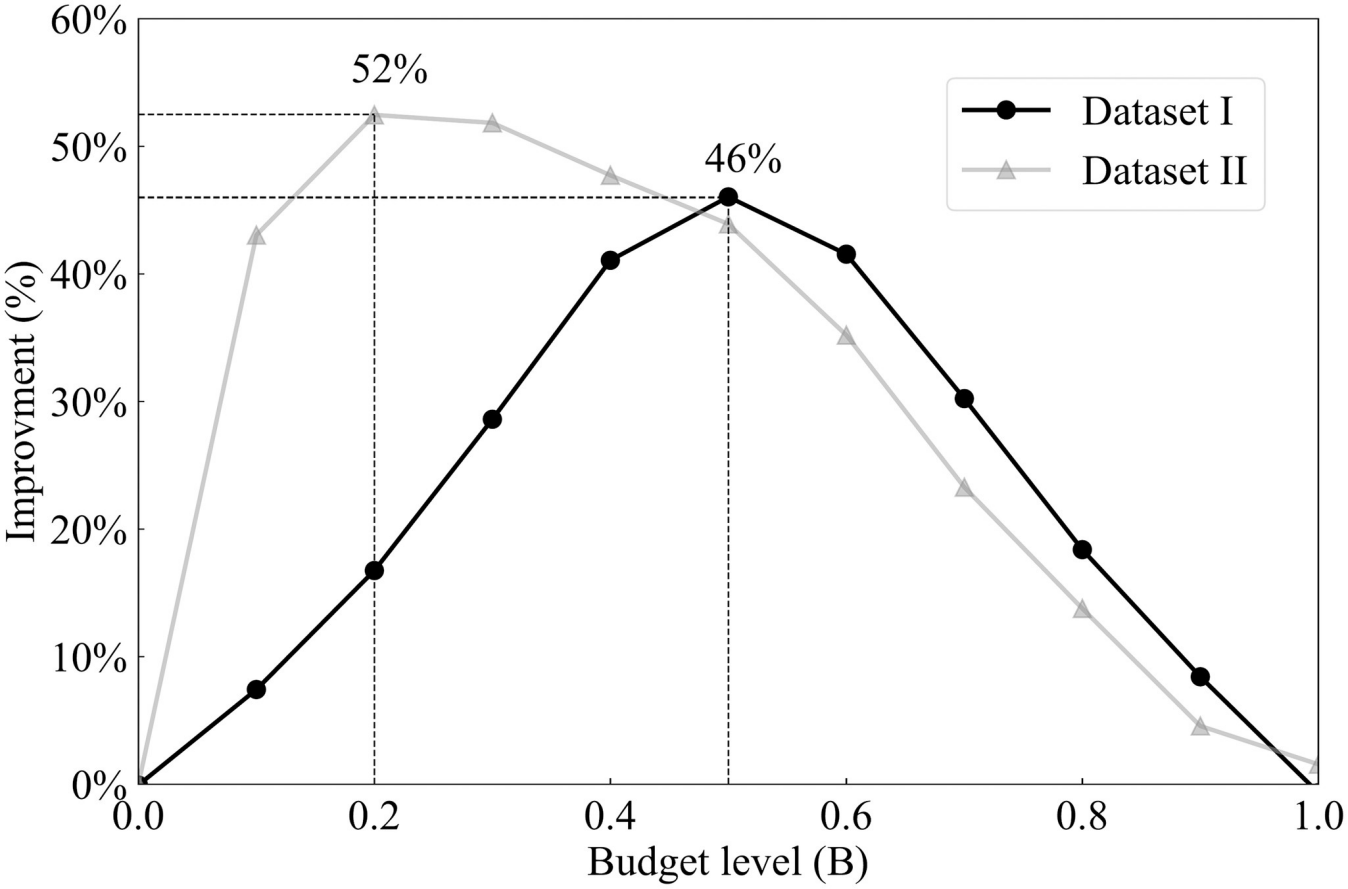
Percent improvement of optimal screening policy (RP-MS) over conventional policy for dataset I (black line) and dataset II (grey line) as a function of budget level, *B*.

Fig 2 plots the percentage improvement of our proposed optimal strategy over the conventional strategy across a wide range of budget levels for datasets I and II. In particular, the black and grey line respectively represent the improvement for datasets I and II. The figure reveals several interesting behaviors: First, it is evident that our proposed screening strategy consistently outperforms the conventional policy across the entire budget spectrum, as indicated by the value of improvement above the x-axis. More interestingly, a bell-shaped pattern can be observed and the peak improvement or bell-shaped trend occurs due to the following: Initially, when *B* = 0, there is no distinction between the two methods. As the budget *B* increases, our method starts to outperform the conventional approach. This improvement continues until the budget reaches a point where it is sufficient for our strategy to potentially test all subjects. Beyond this, any further increase in the budget doesn’t enhance our method’s performance, which then plateaus, whereas the conventional policy keeps improving with more budget. This leads to a peak in the rate of improvement, after which it declines. In addition, we observed improvements of up to 46% (when B = 0.5) and 52% (when B = 0.2) for datasets I and II, respectively. On average, there is an improvement of 21% and 27% for datasets I and II, respectively. Lastly, it is important to understand why the most significant improvements at different budget levels occurred for two distinct datasets. These datasets represent varying scenarios: Dataset I with high COVID-19 but low flu prevalence, and Dataset II with low COVID-19 but high flu. Therefore, the results indicate that a higher prevalence of COVID-19 in Dataset I requires a larger budget to effectively test all potentially infected individuals, which is an intuitive outcome. Conversely, Dataset II, with fewer COVID-19 cases, might necessitate a smaller budget to accomplish comprehensive testing.

The model assumes average values for various parameters, including the sensitivity (*Se*) and specificity (*Sp*) of the test, as well as the costs associated with false negatives (e.g., the cost of hospitalization) and false positives (e.g., the cost of an additional confirmation test). However, the costs of false negatives and false positives ultimately influence the values of the weight parameter λ across different categories. Therefore, to assess the model’s robustness, we conduct a sensitivity analysis for these three parameters: *Se*, *Sp* and **λ** = [λ_*m*_]. In particular, according to the broad scope of studies in the literature, the test sensitivity of RT-qPCR and RAT ranges from 0.9 to 0.99 [115–117]. On the other hand, the specificity of RT-qPCR and RAT is more consistent and ranges from 0.95 to 1 [116, 118]. Consequently, for our analysis, we consider *Se* ∈ {0.9, 0.95, 0.99} and *Sp* ∈ {0.95, 0.97, 1}. In relation to **λ**, the weight parameter λ_*m*_ for each category *m* can be calculated by:

$$\begin{array}{cc} \lambda_{m}= & \frac{w_{FN}}{w_{FN}+w_{FP}}=\frac{C_{H}\cdot h_{m}}{C_{H}\cdot h_{m}+C_{t}}, \end{array}$$

where *w*_*FN*_ and *w*_*FP*_ represent the cost of false negatives and false positives, respectively. Here, *C*_*H*_ is the cost of hospitalization, *h*_*m*_ represents the hospitalization rate for category *m*, and *C*_*t*_ denotes the cost additional confirmatory tests. We have specified the range for *C*_*H*_ as [31, 339$, 111, 213$], for *C*_*t*_ as [10$, 90$], and *h*_*m*_ is held constant at a certain time. It can be easily shown that λ_*m*_ is increasing in *C*_*H*_ and decreasing in *C*_*t*_. Therefore, the maximum and minimum of λ_*m*_, which are denoted as ${\bar{\lambda }}_{m}$ and ${\underline{\lambda }}_{m}$ respectively, can be expressed as:

$$\begin{array}{c} {\bar{\lambda }}_{m}=\frac{111,213\cdot h_{m}}{111,213\cdot h_{m}+10}\;\text{and}\;{\underline{\lambda }}_{m}=\frac{31,339\cdot h_{m}}{31,339\cdot h_{m}+90}. \end{array}$$

We denote the vector form of λs across different categories as $\bar{\lambda }=\left[{\bar{\lambda }}_{m}\right]$, $\tilde{\lambda }=\left[{\tilde{\lambda }}_{m}\right]$ and $\underline{\lambda }=\left[{\underline{\lambda }}_{m}\right]$, where ${\tilde{\lambda }}_{m}$ represents the λ_*m*_ calculated by the average costs. In the following sensitivity analysis, we consider $\lambda \in \{\bar{\lambda },\tilde{\lambda },\underline{\lambda }\}$.

For the sensitivity analysis, we compare the performance of proposed screening strategy with that of conventional screening strategies under different parameter sets. As demonstrated in Table 2, the performance of the optimal screening policy remains consistent across various parameters. Notably, all improvements are positive, indicating that our solution consistently yields enhancements across different scenarios. Furthermore, it is observed that, generally, as the test efficacy improves (i.e., *Se*, *Sp* → 1), the magnitude of improvement becomes more pronounced. This outcome is intuitive, as a more accurate test assay not only increases the accuracy of the group testing incorporated within our framework but also enhances the robustness of our highly customized testing framework.

**Table 2:** Percent improvement of optimal screening policy (RPMS) over conventional policy across the entire budget spectrum for dataset I and dataset II under *Se* ∈ {0.9, 0.95, 0.99}, *Sp* ∈ {0.95, 0.97, 1} and $\lambda \in \{\bar{\lambda },\tilde{\lambda },\underline{\lambda }\}$.

Dataset I							
*Se*	*Sp*	$\bar{\lambda }$		$\tilde{\lambda }$		$\underline{\lambda }$	
		Average	Maximum	Average	Maximum	Average	Maximum
0.9	0.95	4.95%	12.82%	8.41%	18.42%	22.20%	30.23%
0.9	0.97	4.91%	13.25%	7.14%	17.58%	12.84%	26.93%
0.9	1	4.96%	13.83%	5.88%	17.94%	8.17%	18.81%
0.95	0.95	22.31%	43.32%	29.30%	48.52%	34.86%	47.32%
0.95	0.97	23.55%	44.56%	25.81%	48.03%	25.96%	46.71%
0.95	1	25.16%	43.58%	21.68%	46.05%	22.11%	47.03%
0.99	0.95	45.70%	62.00%	49.09%	68.95%	47.80%	65.59%
0.99	0.97	45.22%	62.71%	40.19%	64.81%	46.95%	65.34%
0.99	1	43.48%	62.93%	49.32%	63.77%	44.03%	69.92%
Dataset II							
*Se*	*Sp*	$\bar{\lambda }$		$\tilde{\lambda }$		$\underline{\lambda }$	
		Average	Maximum	Average	Maximum	Average	Maximum
0.9	0.95	19.38%	32.67%	28.70%	39.19%	24.31%	37.82%
0.9	0.97	20.27%	33.34%	34.26%	47.36%	22.75%	38.41%
0.9	1	23.70%	40.09%	33.58%	48.12%	21.36%	39.66%
0.95	0.95	24.56%	35.98%	31.36%	43.41%	38.85%	44.32%
0.95	0.97	22.68%	43.41%	27.36%	51.89%	30.97%	47.53%
0.95	1	26.21%	44.87%	38.63%	56.43%	39.96%	49.21%
0.99	0.95	34.52%	49.97%	42.31%	56.10%	43.33%	60.10%
0.99	0.97	36.14%	59.26%	44.18%	62.29%	49.52%	61.78%
0.99	1	36.94%	58.39%	45.53%	60.56%	50.95%	63.39%

**Fig 3:**
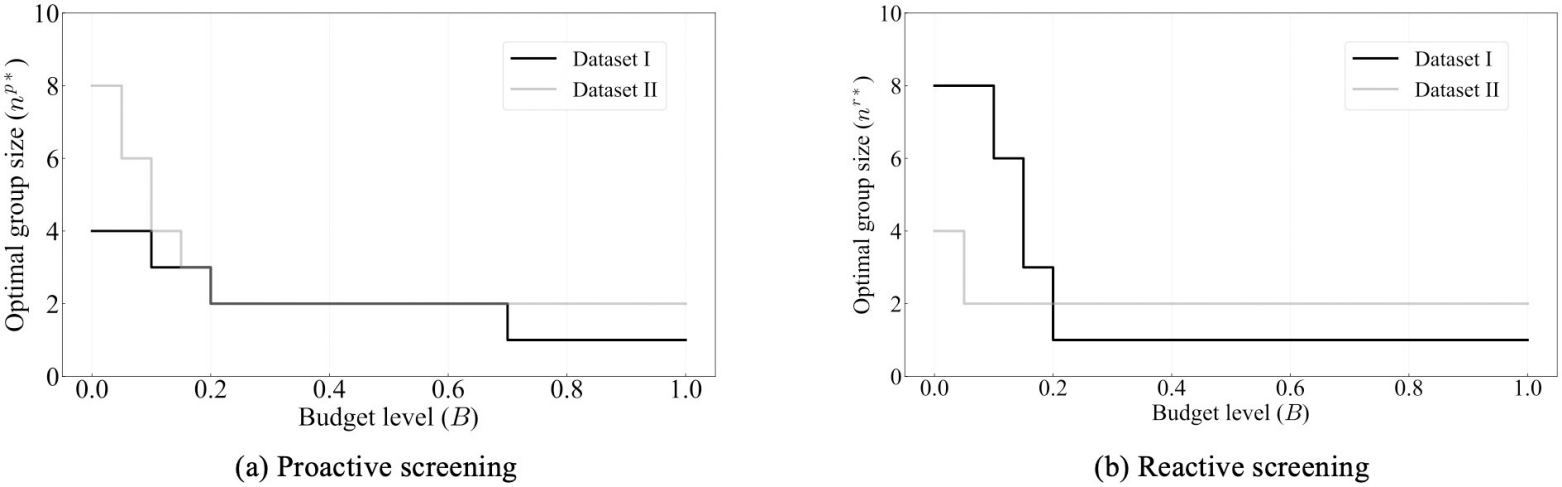
Optimal group size of RP-MS for proactive screening (a) and reactive screening (b) as a function of the budget level *B* for dataset I (black lines) and dataset II (grey lines).

After highlighting the benefits of our screening approach, we will now explore the structure of the optimal solution for **RP-MS**. The first important component of screening design is the testing schemes. Recall that individual testing can be seen as a special case of group testing with a group size of one in **RP-MS**. Fig 3 illustrates the optimal group sizes for proactive and reactive screening at different budget levels. The black and grey lines in the figure correspond to datasets I and II, respectively. The plot provides valuable managerial insights on selecting the appropriate group size at different budget levels and reveals several valuable insights. First, as shown in the plot, group testing is the preferred option for most budget levels as *n*^p*^, *n*^r*^ ≥ 2 for the majority of the budget spectrum. Second, we observe that the optimal group size generally decreases as the budget increases for both datasets. This trend is intuitive, as a larger group size is used to expand testing capacity when the budget is low. However, as the budget increases, a smaller group size is favored to improve screening accuracy. For dataset I, individual testing will eventually become more favorable for both proactive and reactive screening, while for dataset II, individual testing will not be utilized even when the budget becomes affordable. Since the λ value for dataset II is low, indicating that false negatives are less concerning, individual testing provides a more favorable false negative rate, it does not become the preferred option. Third, we observe that datasets I and II adopt opposite testing combinations. In dataset I, proactive screening employs smaller group sizes, while reactive screening utilizes larger groups. Conversely, in dataset II, reactive screening employs smaller group sizes, while proactive screening utilizes larger groups. This behavior can be explained by noting that these two datasets describe inverse scenarios for COVID-19 and flu.

### 4.2 Composition of the optimal solution structure across budget levels

**Fig 4:**
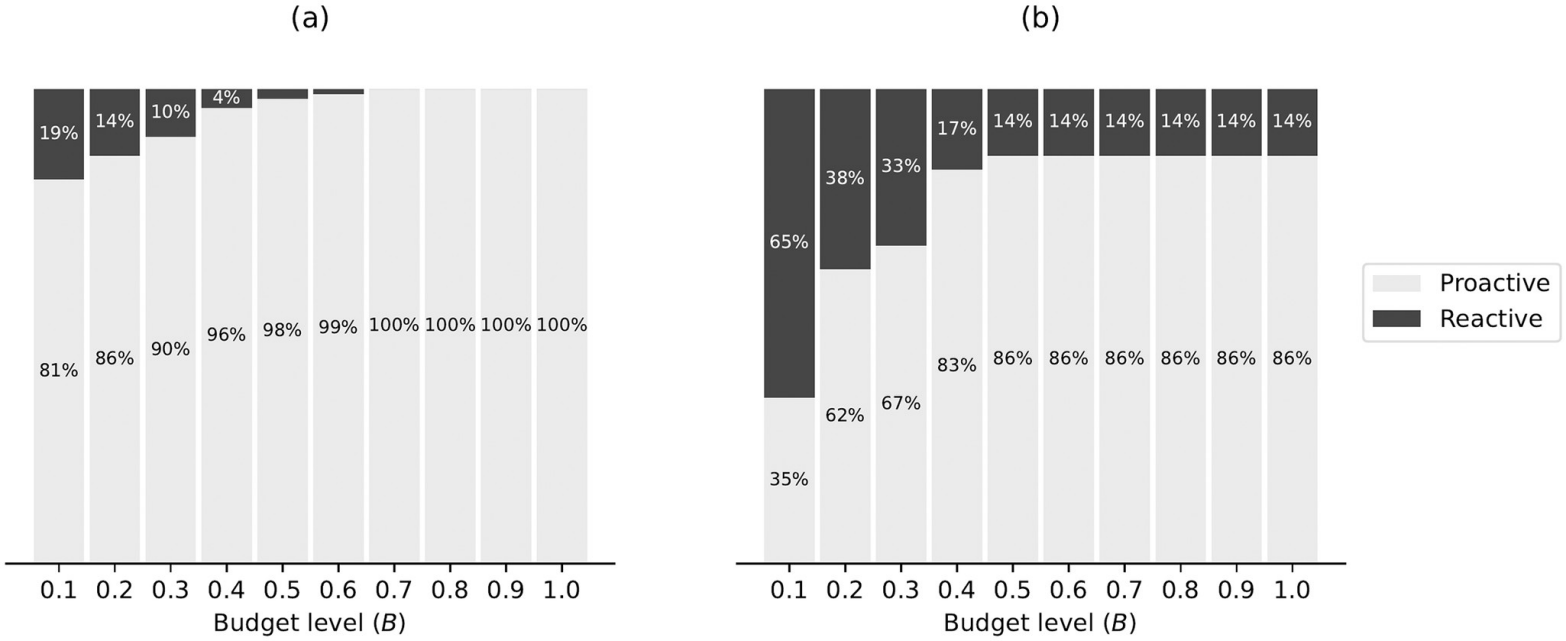
The proportion of reactive and proactive screening in the optimal RP-MS solution for dataset I (a) and dataset II (b), as a function of the budget level *B*.

The choice of reactive/proactive screening is an important aspect of the screening design. The proportion of counties that are tested reactively/proactively with varying budget levels is illustrated in Fig 4. In particular, the grey and black bars respectively represent the proportion of proactive and reactive screening with two datasets. The figure shows that the proportion of proactive screening increases as the budget level increases for both datasets. Therefore, the optimal solution suggested by **RP-MS** is to prioritize proactive screening as the budget increases instead of reserving testing budget for symptomatic cases. Furthermore, by comparing the proportions of the two datasets, we can observe that when the budget is low, dataset II has a significantly higher proportion of reactive testing than dataset I. This is not surprising, given that dataset II has a higher flu prevalence rate and the flu has a higher symptomatic rate, resulting in a higher overall number of symptomatic cases. Under this scenario, reactive screening is preferred when the budget is low, and the strategy should swiftly shift to proactive screening as the budget increases. On the other hand, for dataset I, the majority of counties are tested proactively. This observation aligns with the significantly high prevalence rate of COVID-19 within the dataset, since proactive screening is more effective in controlling the disease. As the budget becomes sufficient, proactive screening will be utilized for all counties in dataset I. However, in the case of dataset II, reactive screening will still remain the preferred strategy for some counties with higher symptomatic rates, even as the budget increases.

### 4.3 Optimal testing design and budget allocation across geographic regions

**Fig 5:**
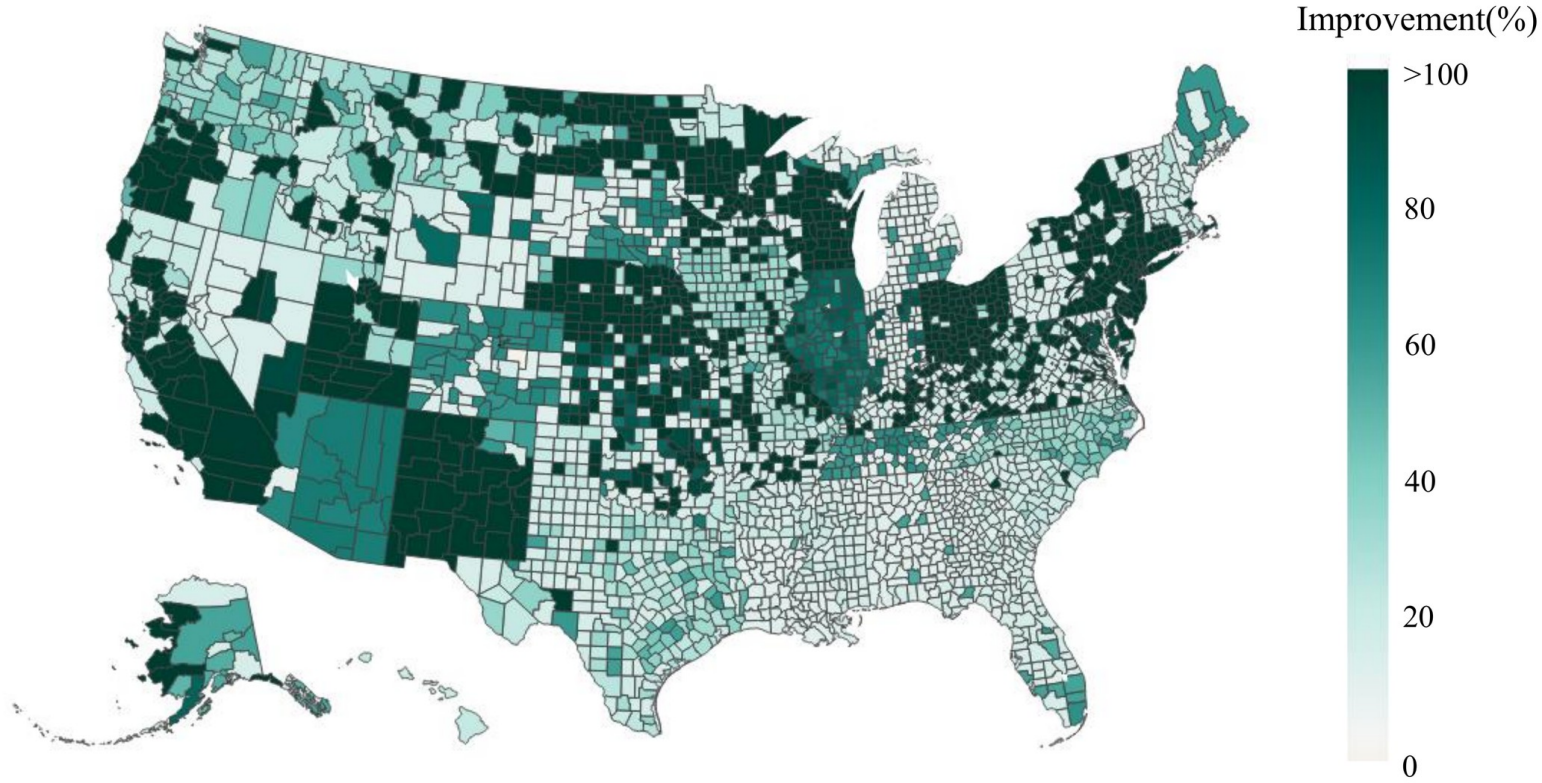
Percent improvement of optimal screening policy (RP-MS) over conventional policy across 3,143 counties in the US when *B* = 0.1 for dataset II [generated by Plotly in Python [94]].

Considering that the dataset is geographic-based, we can leverage this characteristic to explore the structure of the optimal solution in correlation with different geographic regions. In what follows, we conduct an analysis under a specific budget level of *B* = 0.1 which represents a scenario where the budget is limited. Regarding the dataset selection, Fig 4 illustrates that dataset II displays a more noticeable distinction in the proportion of reactive and proactive behaviors. Therefore, we select dataset II for this analysis. However, we emphasize that this analysis is for demonstration purposes only, and similar analyses could be conducted on different datasets or with different budget levels. Fig 5 illustrates the improvement of **RP-MS** over conventional screening strategies across 3,143 counties, with darker green colors indicating higher levels of improvement. Note that Fig 5 serves for illustrative purposes and similar analyses can be undertaken across different budget levels and datasets. Fig 5 unveils several enlightening findings. Firstly, it shows positive improvements across all counties, demonstrating that our solution enhances overall performance without negatively impacting specific counties compared to the conventional strategy. Secondly, comparing Fig 5 with Fig 1b reveals a trend: The optimal strategy yields more significant improvements in counties with higher risk levels. This suggests that our framework recommends prioritizing counties with greater risk levels, which is a logical approach for mitigating disease transmission by focusing on high-risk areas. This underscores the critical need to account for the heterogeneity of the population in devising intervention strategies.

**Fig 6:**
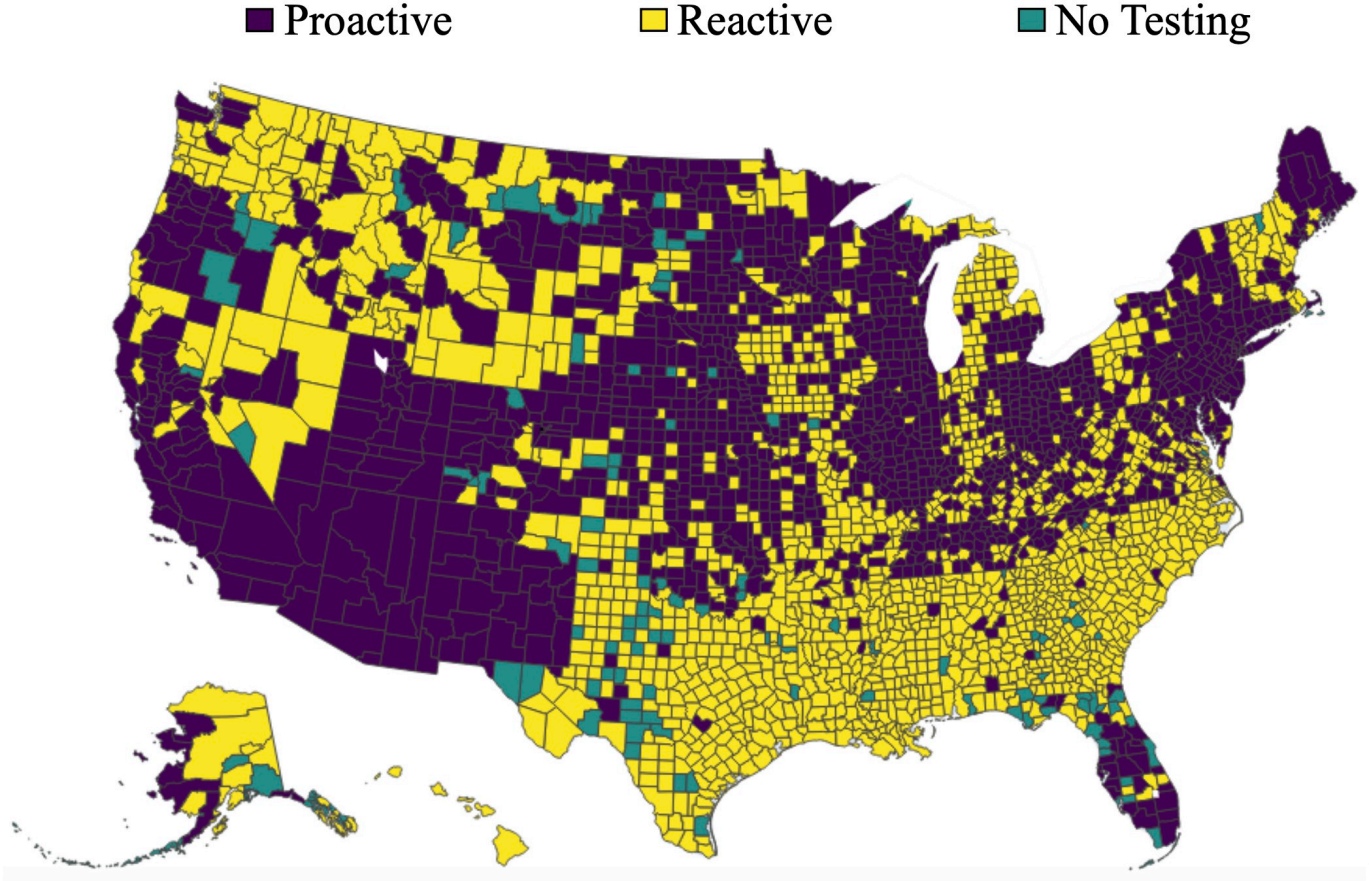
Distribution of the optimal reactive/proactive screening across counties in the U.S. under the budget level *B* = 0.1 for dataset II by RP-MS [generated by Plotly in Python [94]].

The geographic-based data can capture managerial insights regarding the distribution of screening strategies. Fig 6 presents a heatmap of the U.S. depicting the distribution of reactive and proactive screening choices across different counties. Counties subject to proactive (reactive) testing are denoted by the color purple (yellow), while those subjects are not tested are denoted by the color green. The figure unveils several intriguing patterns and offers valuable managerial insights. First, the figure shows that a majority of counties are tested even when budgets are limited, as shown by the majority of counties highlighted in yellow or purple. This can be attributed to the implementation of group testing, where an optimal group size of *n*^p*^ = 8 and *n*^r*^ = 4 are utilized (see Fig 3), significantly expanding screening efforts. Second, the counties that have not been tested, highlighted in green, have a remarkably low prevalence rate of both COVID-19 and flu in Fig 1b and 1d. This observation suggests that the counties at higher risk are prioritized for testing, which is intuitive. Third, we observe a mix of proactive and reactive screening strategies for tested counties (highlighted in purple and yellow). As observed in Fig 1b, counties with higher prevalence rate, like those in New Mexico and Arizona, are more likely to benefit from proactive testing. This approach remains favored in these areas, irrespective of their flu rates. For example, despite its high COVID-19 prevalence and lower flu rates, Arizona, alongside California and New Mexico, which also report high COVID-19 but low flu rates, consistently sees recommendations for proactive screening. This aligns with expectations, as proactive strategies are typically more effective in high-risk areas, helping to curb the disease transmission. Conversely, regions like Washington and Montana, where flu prevalence is high but COVID-19 prevalence is low, are more inclined towards reactive testing strategies. This is logical since areas with significant flu activity often experience higher symptomatic presentations, necessitating reactive screening to differentiate between flu and COVID-19 symptoms. In conclusion, this numerical analysis offers valuable insights into the strategic allocation of screening strategy, emphasizing the benefits of tailored proactive and reactive approaches based on local prevalence rates. Furthermore, we should anticipate a greater emphasis on proactive screening as the overall prevalence increases. While our findings provide a intuitive insights for decision-makers, the precise design of mass screening programs should be generated by using optimization models.

**Fig 7:**
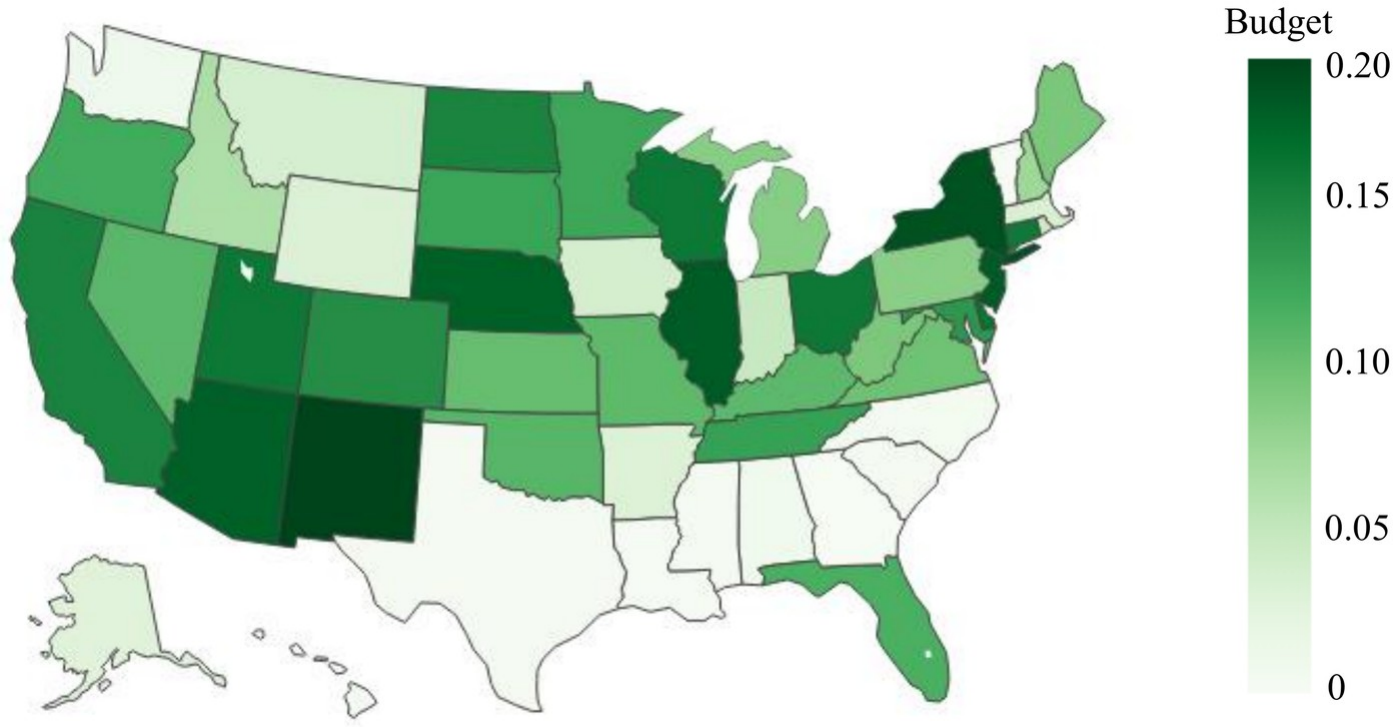
Optimal state-level budget allocation per capita across the US when *B* = 0.1 for dataset II by RP-MS [generated by Plotly in Python [94]].

The geographic-based data can also provide crucial managerial insights regarding budget allocation. In Fig 7, we visualize the state-level allocation of budgets per capita, as the more detailed county-level allocation may not be the most practical option for decision-makers. In particular, the depth of color represents different levels of budget allocation, with darker colors indicating higher values. Comparing it to Fig 6, we observe that states adopting proactive screening (e.g., southwestern areas) receive higher budgets compared to those adopting reactive screening (e.g., South and North Carolina). This is because proactive screening generally incurs higher costs than reactive screening, as the latter targets symptomatic cases, which are typically fewer in number compared to non-symptomatic cases. Therefore, counties that implement proactive screening, especially those with higher COVID-19 risks (as shown in Fig 4), require additional testing budget.

### 4.4 Analysis of generalized model CRP-MS

After conducting a comprehensive analysis and examining the performance and solution structure of **RP-MS**, we now turn our attention to investigating the generalized model **CRP-MS**. In comparison to **RP-MS**, **CRP-MS** offers increased customizability, allowing for the implementation of tailored testing scheme to each category. While this high level of customization might present practical challenges, it has the potential to further decrease classification errors. Hence, it is crucial to examine the the potential improvements in performance that can be achieved through increased customization and quantify the trade-offs between practicability and performance.

Before delving into the performance analysis of **CRP-MS**, we first evaluate the efficiency and accuracy of the proposed heuristic solution scheme presented in Algorithm 1 and compare it to commercial solvers. Table 3 presents the performance comparison between our proposed heuristic solution scheme and Gurobi [125] in terms of optimal objective values and computational time. To prevent the scenario in which the solver fails to converge to an optimal solution, we have set an upper time limit of 2 hours (7200 seconds). Upon examining Table 3, it is evident that our heuristic solution consistently delivers similar performance to Gurobi for dataset I and even outperforms Gurobi for dataset II across various budget levels. The reason for the significant improvement for dataset II is primarily due to Gurobi’s inability to converge within the time limit. Additionally, we observed that the heuristic solution significantly reduces computational time compared to Gurobi. This computational advantage becomes particularly evident in dataset II. One possible explanation is that in dataset I, a substantial number of variables can be eliminated during the preprocessing procedure. On the other hand, dataset II comprises a larger number of competitive testing options that need to be considered. Consequently, more variables remain in the solver system after the preprocessing procedure, resulting in a significant increase in computational time. Another interesting behavior is observed in dataset I, where the computational time becomes faster when the budget exceeds the upper bound (e.g., *B* > 0.5 for dataset I). This can be attributed to the relaxation of the budget constraint, allowing the solver to more efficiently find solutions within the relaxed constraint and thus leading to faster computation time. In summary, our proposed solution scheme not only delivers superior solution quality compared to the commercial solver, but also demonstrates enhanced stability across various datasets, offering significant computational advantages. These computational benefits are particularly pronounced when dealing with larger problem instances that involve more categories or testing options.

**Table 3:** Performance comparison, in terms of optimal objective values and computational time, between the proposed heuristic solution and Gurobi 10.0 for CRP-MS with two datasets.

Dataset	*B*	Objective Value		Time	
		Gurobi 10.0	Heuristic	Gurobi 10.0	Heuristic
I	0.1	1.78%	1.78%	311s	1s
	0.3	0.82%	0.84%	633s	1s
	0.5	0.24%	0.27%	42s	3s
	0.7	0.22%	0.22%	30s	5s
	0.9	0.22%	0.22%	30s	7s
II	0.1	1.19%	0.85%	7200s	1s
	0.3	0.26%	0.38%	7200s	2s
	0.5	0.30%	0.28%	7200s	3s
	0.7	0.30%	0.28%	7200s	5s
	0.9	0.30%	0.28%	7200s	8s

**Fig 8:**
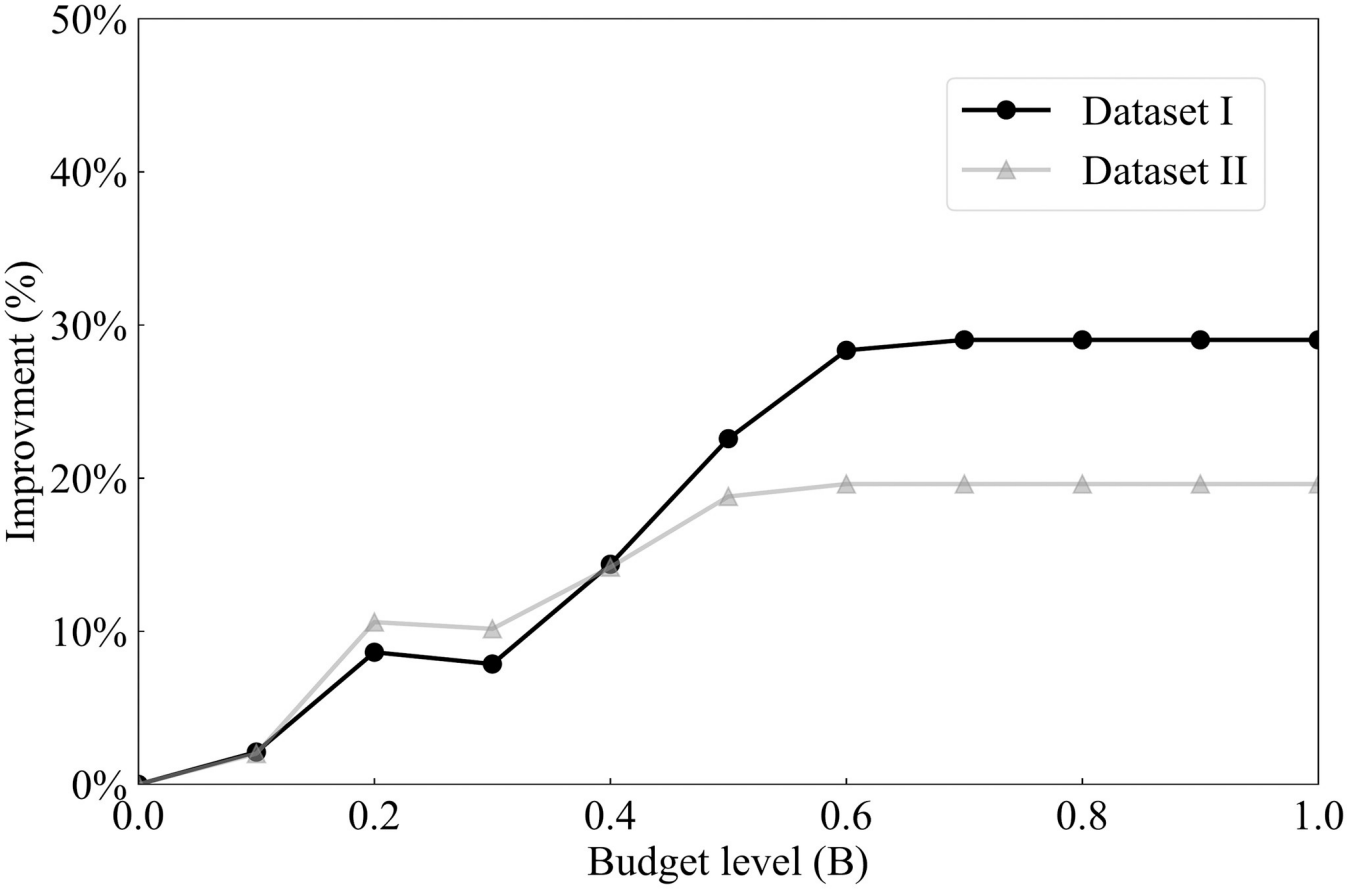
Percent improvement of CRP-MS over RP-MS for dataset I (black line) and dataset II (grey line) as a function of budget level, *B*.

Next, we compare the performance of **CRP-MS** with **RP-MS** across different budget levels. Fig 8 describes the percent improvement of **CRP-MS** over **RP-MS** with two datasets as a function of budget levels *B*. In particular, the black and grey line respectively represent the percent improvement for datasets I and II. The figure reveals several insightful results: First, **CRP-MS** consistently outperforms **RP-MS** across the entire budget spectrum. This result is expected, considering that **RP-MS** can be treated as a specific case of **CRP-MS** by imposing $n_{1}^{p}=n_{2}^{p}=\cdots =n_{M}^{p}$ and $n_{1}^{r}=n_{2}^{r}=\cdots =n_{M}^{r}$. Secondly, the improvement steadily increases with an increment in the budget and eventually reaches a steady state. This trend arises due to the limited flexibility of **RP-MS** when the budget is low, as there is little room for customization. However, as the budget increases, the advantages of personalized testing schemes become more pronounced, leveraging the available resources more effectively. This trend continues until the budget reaches its upper limit, achieving the maximum improvement and settling into a steady state. At this point (*B* ≈ 0.6), additional budget allocation does not lead to further performance enhancement as the optimal performance has already been attained. To summarize, the utilization of **CRP-MS** yields an average improvement of 18% and up to 14% over **RP-MS** across two datasets. These results offer valuable insights to decision-makers, enabling them to assess the trade-offs associated with customization and practical considerations. For instance, in scenarios with a low budget (e.g., *B* = 0.2), decision-makers have the option to prioritize performance over practicality. By incorporating more customization, they can achieve an additional 10% reduction in the misclassification rate. Overall, it is advantageous to adopt **RP-MS** due to its ease of practical implementation when the budget is limited. As the testing budget becomes more available, transitioning to **CRP-MS** yields greater benefits.

**Fig 9:**
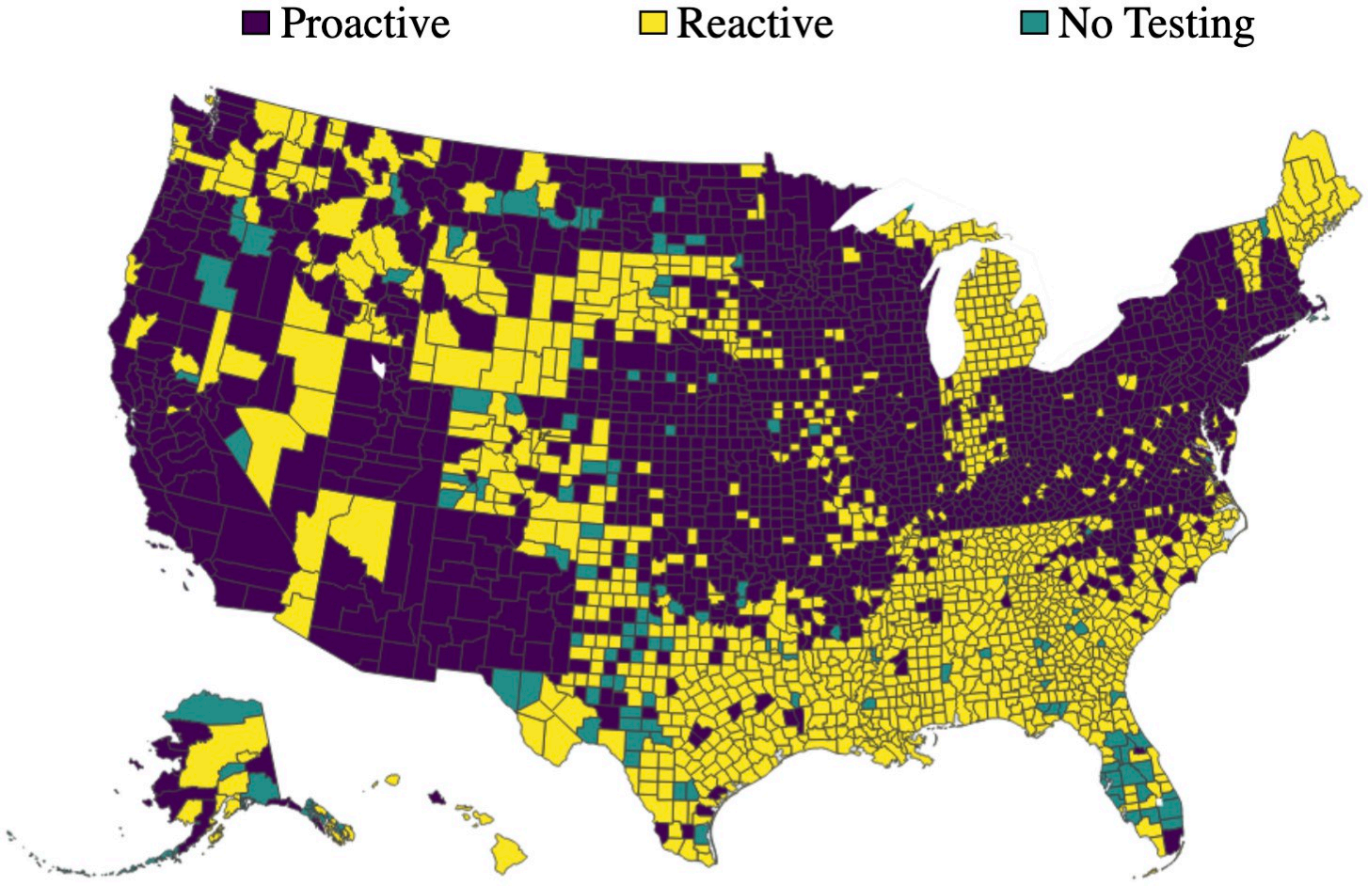
Distribution of the optimal reactive/proactive screening across counties in the U.S. under the budget level *B* = 0.1 for dataset II by CRP-MS [generated by Plotly in Python [94]].

**Fig 10:**
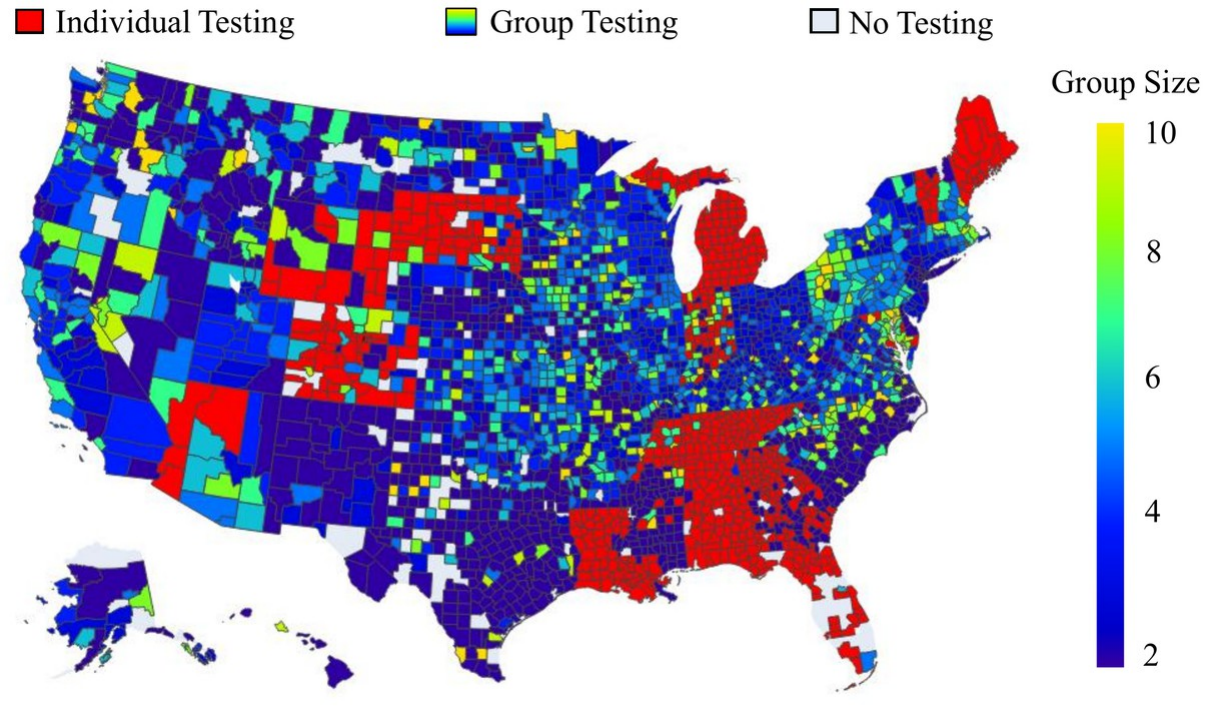
Distribution of the optimal testing schemes across counties in the U.S. under the budget level *B* = 0.1 for dataset II by CRP-MS [generated by Plotly in Python [94]].

Lastly, we investigate the optimal solution structure of **CRP-MS** across geographic regions. Note that the following analysis can also be conducted for varying budget levels and datasets. In order to make a comparison with the analysis of **RP-MS**, we conduct the analysis under the same parameter setting as discussed in Section 4.3. First, similar to the findings presented in Fig 6 for **RP-MS**, we illustrate the distribution of proactive/reactive screening for dataset II when the budget *B* = 0.1. Fig 9 shows the distribution of proactive/reactive screening of **CRP-MS**, and the overall trends remain consistent with those observed in Fig 6. To demonstrate the customization of testing schemes, Fig 10 illustrates the distribution of testing schemes across counties as determined by **CRP-MS** via a heatmap. In particular, individual testing is denoted by the color red, while counties without any testing are depicted in light gray. Counties subjected to group testing are represented using a color gradient, where the hue varies based on the optimal size of the testing group. The figure clearly demonstrates that the testing schemes vary significantly across different counties. Combining the information from Fig 10 with Fig 9, we observe that, in general, the model tends to utilize group testing in proactive screening and individual testing in reactive screening. This approach makes sense since proactive screening generally incurs higher costs than reactive screening, and adopting group testing can significantly expand screening efforts. Furthermore, we notice that among counties that are tested through group/reactive screening, counties with higher COVID-19 prevalence (e.g., New Mexico) tend to adopt smaller group sizes to ensure screening accuracy. This finding indicates that counties with a higher burden of COVID-19 prioritize accuracy over cost-effectiveness when implementing group testing strategies.

## 5 Discussion: Conclusions and future research directions

In this paper, we study the problem of designing an optimal mass screening strategy to minimize classification errors. The proposed framework incorporates proactive and reactive screening and determines the portion of the budget to target sub-populations and the budget to screen symptomatic cases under limited testing budget. In addition, the framework considers various testing schemes, population heterogeneity and imperfect tests. By analyzing the structure of the formulations, we first enhance the tractability of the resulting problem by transforming it into a more tractable bilinear optimization problem. Subsequently, we take advantage of the special knapsack style structure of the resulting problem to identify key structural properties. Utilizing one of these properties as a preprocessing step enables us to eliminate a significant number of decision variables in advance, greatly reducing computational expenses. Moreover, these identified properties serve as the foundation for constructing an efficient solution scheme that guarantees its global optimality. Furthermore, we extend the model by integrating tailored testing schemes across categories and present a highly efficient heuristic solution algorithm. The numerical experiments provide compelling evidence of the heuristic solution scheme’s effectiveness when compared to commercial solvers. We conduct a case study employing real-world COVID-19 data. Our findings demonstrate a substantial and consistent improvement over conventional screening strategies. Moreover, the numerical results obtained from our study offer decision-makers invaluable managerial insights into optimizing the distribution of proactive and reactive screening, as well as budget allocation across various regions. Lastly, we evaluate the performance of the generalized model and engage in a discussion regarding the balance between customizability and practicality.

This work can be expanded in several exciting directions. First, a possible extension is the incorporation of additional testing schemes, such as array-based and multi-stage group testing. Although implementing these extensions in practice may be more complex, they have the potential to enhance the classification accuracy of the screening strategy. By including these testing schemes, we can further refine and improve the accuracy of our screening strategy. Second, another research direction is to consider the availability of different testing assays. The resulting framework would become more customized, especially when dealing with a heterogeneous population. For instance, it may be more beneficial to proactively test individuals using an assay with higher accuracy and reserve the assay with lower accuracy. Third, it would be beneficial to integrate the decision problem of defining the sub-populations directly into the framework, rather than pre-defining the sub-populations in advance. Lastly, the proposed framework can be extended to a dynamic problem by incorporating the time component. This is challenging as it requires integrating epidemiological models such as the SIR model within an optimization framework, thereby resulting in a multi-stage optimization problem. We hope this work establishes the groundwork for proactive/reactive screening strategies and inspires further research in the aforementioned areas.

## Supporting information

S1 File **A: Derivations of Symptomatic Rate.** **B: Detailed Derivations of Performance Metrics.** **B.1: Proactive Testing.** **B.2: Reactive Testing.** **B.3: Derivation of the Optimization Model.** **C: Mathematical Proofs.** **D: Structural Properties of CRP-MS**(z*). **E: Heuristic Solution Scheme for CRP-MS**(z*). **F: Calibration of Symptomatic Rates**.
(PDF)
