# Supplementary material for: A proactive/reactive mass screening approach with uncertain symptomatic cases
Source: PLoS Comput Biol. 2024 Aug 14;20(8):e1012308. doi: 10.1371/journal.pcbi.1012308 (PMC11346970; doi:10.1371/journal.pcbi.1012308)
Supplement: S1 File — A: Derivations of Symptomatic Rate. B: Detailed Derivations of Performance Metrics. B.1: Proactive Testing. B.2: Reactive Testing. B.3: Derivation of the Optimization Model. C: Mathematical Proofs. D: Structural Properties of CRP-MS(z*). E: Heuristic Solution Scheme for CRP-MS(z*). F: Calibration of Symptomatic Rates. (PDF) [file pcbi.1012308.s001.pdf]

S1 File

A Derivations of Symptomatic Rate

In this section, our objective is to calculate the probability of a subject, denoted as w, belonging to category m, experiencing symptoms (i.e., $P(s_w = 1|w \in \mathcal{L}_m)$). These symptoms may arise as a result of the primary disease as well as other diseases that exhibit similar symptoms. Let us denote S_w as a binary random variable representing the symptomatic status of a random subject w, with $S_w = 1$ indicating a symptomatic case, $S_w = 0$ otherwise. Similarly, let $I_w(F_w)$ be a binary random variable representing the true infectivity status of the targeted disease (non-targeted disease with similar symptoms), with $I_w = 1(F_w = 1)$ indicating a positive status and $I_w = 0(F_w = 0)$ otherwise. In addition, we denote the symptomatic rate of non-targeted disease is α_m for category m. First, conditioning the true infectivity status of the targeted disease on subject w gives:

$$
\begin{aligned}
P\big(s_w = 1|w \in \mathcal{L}_m\big) =& P\big(s_w = 1|I_w = 1, w \in \mathcal{L}_m\big)P\big(I_w = 1|w \in \mathcal{L}_m\big) && (1)\\
&+ P\big(s_w = 1|I_w = 0, w \in \mathcal{L}_m\big)P\big(I_w = 0|w \in \mathcal{L}_m\big) && (2)
\end{aligned}
$$

Further conditioning the expected term in Eq. (1) on the true infectivity status of non-targeted disease gives;

$$
\begin{aligned}
P\big(s_w = 1|I_w = 1, w \in \mathcal{L}_m\big) =& P\big(s_w = 1|I_w = 1, F_w = 1, w \in \mathcal{L}_m\big)P\big(F_w = 1|I_w = 1, w \in \mathcal{L}_m\big)\\
&+ P\big(s_w = 1|I_w = 1, F_w = 0, w \in \mathcal{L}_m\big)P\big(F_w = 0|I_w = 1, w \in \mathcal{L}_m\big)\\
=& P\big(s_w = 1|I_w = 1, F_w = 1, w \in \mathcal{L}_m\big)P\big(F_w = 1|w \in \mathcal{L}_m\big)\\
&+ P\big(s_w = 1|I_w = 1, F_w = 0, w \in \mathcal{L}_m\big)P\big(F_w = 0|w \in \mathcal{L}_m\big) && (3)
\end{aligned}
$$

The last equation is obtained by first noting that we assume being infected by different diseases (target and non-target) are independent. Then, if subject is infected by both target disease and non-target disease, the probability of showing symptoms is given by:

$$
\begin{aligned}
P\big(s_w = 1|I_w = 1, F_w = 1, w \in \mathcal{L}_m\big) =& 1 - P\big(s_w = 0|I_w = 1, F_w = 1, w \in \mathcal{L}_m\big)\\
=& 1 - (1 - s_m)(1 - \alpha_m). && (4)
\end{aligned}
$$

On the other hand, if the subject is only infected by the targeted disease, the probability of showing symptoms is given by:

$$
P\big(s_w = 1|I_w = 1, F_w = 0, w \in \mathcal{L}_m\big) = s_m. \quad (5)
$$

Substituting Eqs. (4) and (5) into (3) gives:

$$
P\big(s_w = 1|I_w = 1, w \in \mathcal{L}_m\big) = \Big[1 - (1 - s_m)(1 - \alpha_m)\Big]f_m + s_m(1 - f_m). \quad (6)
$$

Similarly, conditioning the probability term in Eq. (2) on the true infectivity status of the targeted disease:

$$\begin{aligned}P\big(s_w = 1|I_w = 0, w \in \mathcal{L}_m\big) =&P\big(s_w = 1|I_w = 0, F_w = 1, w \in \mathcal{L}_m\big)P\big(F_w = 1|I_w = 0, w \in \mathcal{L}_m\big)\\ &+ P\big(s_w = 1|I_w = 0, F_w = 0, w \in \mathcal{L}_m\big)P\big(F_w = 0|I_w = 0, w \in \mathcal{L}_m\big)\\ =&P\big(s_w = 1|I_w = 0, F_w = 1, w \in \mathcal{L}_m\big)P\big(F_w = 1|w \in \mathcal{L}_m\big)\\ =&\alpha_m f_m. \end{aligned} \tag{7}$$

The last equation is obtained by noting that if subject w is not infected by any disease, the symptom will not occur. Next, substituting Eqs. (6) and (7) into Eq. (1) and (2) gives:

$$\begin{aligned}P\big(s_w = 1|w \in \mathcal{L}_m\big) =&\Big[\big[1-(1-s_m)(1-\alpha_m)\big]f_m + s_m(1-f_m)\Big]r_m + \alpha_m f_m(1-r_m)\\ =&a_m f_m + (1-a_m f_m)s_m r_m \end{aligned} \tag{8}$$

To facilitate the presentation, let us use L_m to denote the symptomatic rate:

$$L_m = a_m f_m + (1-a_m f_m)s_m r_m. \tag{9}$$

B Detailed Derivations of Performance Metrics

To derive the performance measures, we denote a random subject w that is randomly selected from the entire population. Due to the involvement of a series of conditional probabilities in the derivation process, new decision variables are introduced for derivation purpose. That is, for the testing scheme used on tested subjects, let $y_m^p \in \{0,1\}$ ($y_m^r \in \{0,1\}$) be a binary decision variable that takes the value of 1 if subjects are proactively (reactively) tested via individual testing and 0 group testing. These binary decision variables will be eliminated upon completion of the derivation. By first conditioning on the category that subject w belongs to, the expected probability of classifying subject w as a false negative $\mathbb{E}[FN]$ (to facilitate the presentation, $(\boldsymbol{x}^p, \boldsymbol{x}^r, \boldsymbol{y}^p, \boldsymbol{y}^r, \boldsymbol{n}^p, \boldsymbol{n}^r, \boldsymbol{z})$ will be dropped from the expressions) for a given policy is given by:

$$\mathbb{E}\big[FN\big] = \sum_{m=1}^{M} \mathbb{E}\big[FN|s \in \mathcal{L}_m\big]P\big(s \in \mathcal{L}_m\big). \tag{10}$$

To facilitate the analysis, let us denote the term $\mathbb{E}\big[FN|s \in \mathcal{L}_m\big]$ as $\mathbb{E}_m\big[FN\big]$. Next, let $\boldsymbol{\Omega}(x_m^p)$ denote the subset of subjects that are proactively tested in category m and further conditioning on weather the subject is proactively tested or not, the term $\mathbb{E}_m[FN]$ can be written as:

$$\mathbb{E}_m[FN] =\mathbb{E}_m\big[FN\big|w \in \Omega(x_m^p)\big]P\big(w \in \Omega(x_m^p)\big) + \mathbb{E}_m\big[FN\big|w \notin \Omega(x_m^p)\big]P\big(w \notin \Omega(x_m^p)\big) \tag{11}$$

where Eq.(11) is obtained by denoting the first expected term $\mathbb{E}_m\big[FN\big|w \in \Omega(x_m^p)\big] = \mathbb{E}_m^p\big[FN\big]$ that represents the false negatives in the proactive testing subset, and denote the second expected term as $\mathbb{E}_m\big[FN\big|w \notin \Omega(x_m^p)\big] = \mathbb{E}_m^{\overline{p}}\big[FN\big]$ that represents the false negatives not in the proactive testing subset. The probability terms in Eq.(11) are equal to the ratio of proactive/non-proactive

testing population size to the total population, which gives:

$$\mathbb{E}[FN] = \sum_{m=1}^{M} \left[\mathbb{E}_m^p\big[FN\big]\frac{p_m x_m^p}{P_T} + \mathbb{E}_m^{\overline{p}}\big[FN\big]\frac{p_m(1-x_m^p)}{P_T}\right]. \tag{12}$$

Similarly, the expected probability of classifying subject w as a false positive $\mathbb{E}[FP]$ and the expected number of tests $\mathbb{E}[TC]$ can be written as

$$\mathbb{E}[FP] = \sum_{m=1}^{M} \left[\mathbb{E}_m^p\big[FP\big]\frac{p_m x_m^p}{P_T} + \mathbb{E}_m^{\overline{p}}\big[FP\big]\frac{p_m(1-x_m^p)}{P_T}\right], \tag{13}$$

$$\mathbb{E}[TC] = \sum_{m=1}^{M} \left[\mathbb{E}_m^p\big[TC\big]\frac{p_m x_m^p}{P_T} + \mathbb{E}_m^{\overline{p}}\big[TC\big]\frac{p_m(1-x_m^p)}{P_T}\right] \tag{14}$$

In what follows, we provide detailed derivation for proactive testing ($\mathbb{E}_m^p[FN]$,$\mathbb{E}_m^p[FP]$ and $\mathbb{E}_m^p[TC]$) and subjects who are not tested via proactive screening (either through reactive screening or classified as untested) ($\mathbb{E}_m^{\overline{p}}[FN]$,$\mathbb{E}_m^{\overline{p}}[FP]$ and $\mathbb{E}_m^{\overline{p}}[TC]$) respectively.

B.1 Proactive Testing

$\underline{\mathbb{E}_m^p[FN]}$: By first conditioning on weather the subject is individually tested or tested via group testing, the term can be written as:

$$\begin{aligned}\mathbb{E}_m^p\big[FN\big] =& \mathbb{E}\big[FN|w \in \Omega^I(x_m^p)\big]P\big(w \in \Omega^I(x_m^p)|w \in \mathcal{L}_m\big) & (15)\\ &+ \mathbb{E}\big[FN|w \in \Omega^G(x_m^p)\big]P\big(w \in \Omega^G(x_m^p)|w \in \mathcal{L}_m\big). & (16)\end{aligned}$$

In what follows, we derive the expected term in Eqs. (15) and (16) respectively. Conditioning the expected term in Eq. (15) on the true infectivity status of subjects w gives:

$$\begin{aligned}\mathbb{E}\big[FN|w \in \Omega^I(x_m^p)\big] =& \mathbb{E}\big[FN|I_w = 1, w \in \Omega^I(x_m^p)\big]P\big(I_w = 1|w \in \Omega^I(x_m^p)\big)\\ &+ \mathbb{E}\big[FN|I_w = 0, w \in \Omega^I(x_m^p)\big]P\big(I_w = 0|w \in \Omega^I(x_m^p)\big)\\ &= \mathbb{E}\big[FN|I_w = 1, w \in \Omega^I(x_m^p)\big]P\big(I_w = 1|w \in \Omega^I(x_m^p)\big), \qquad (17)\end{aligned}$$

where Eq. (17) is obtained by noting that if subject is truly negative ($I_w = 0$), a false negative cannot occur. Given that the subject is tested individually, a false negative will occur with probability $1 - Se$, that is

$$\mathbb{E}\big[FN|I_w = 1, w \in \Omega^I(x_m^p)\big] = 1 - Se.$$

Then, by noting that

$$P\big(I_s = 1|w \in \Omega^I(x_m^p)\big) = r_m,$$

Eq. (17) is given by:

$$\mathbb{E}\big[FN|w \in \Omega^I(x_m^p)\big] = (1 - Se)r_m. \tag{18}$$

Next, to derive the expression of the expected term in Eq. (16), a similar procedure can be adopted:

$$\mathbb{E}\big[FN|w \in \Omega^G(x_m^p)\big] = \mathbb{E}\big[FN|I_w = 1, w \in \Omega^G(x_m^p)\big]r_m. \tag{19}$$

Given that the subject is tested by group testing, then a false neagtive will occur in two scenarios: (i) if the outcome of the first stage is negative (with probability of $1 - Se$), and (ii) if the outcome of the first stage is positive(with probability of Se) and the subsequent individual testing at the second stage is negative (with probability of $1 - Se$). Consequently, we get:

$$\mathbb{E}\big[FN|I_w = 1, w \in \Omega^G(x_m^p)\big] = (1 - Se) + Se(1 - Se) = (1 - Se)^2,$$

which leads to

$$\mathbb{E}\big[FN|w \in \Omega^G(x_m^p)\big] = (1 - Se)^2 r_m. \tag{20}$$

Substituting Eq. (18) and (20) into Eqs. (15) and (16) and by noting that the probability terms in Eqs. (15) and (16) are equal to y_m^p and $(1 - y_m^p)$ leads to:

$$\mathbb{E}_m^p\big[FN\big] = (1 - Se)r_m y_m^p + (1 - Se^2)r_m(1 - y_m^p). \tag{21}$$

$\underline{\mathbb{E}_m^p[FP]}$: Similarly, we first condition on weather the subject is individually tested or tested via group testing, the term can be written as:

$$\mathbb{E}_m^p\big[FP\big] = \mathbb{E}\big[FP|w \in \Omega^I(x_m^p)\big]P\big(w \in \Omega^I(x_m^p)|w \in \mathcal{L}_m\big) \tag{22}$$

$$+ \mathbb{E}\big[FP|w \in \Omega^G(x_m^p)\big]P\big(w \in \Omega^G(x_m^p)|w \in \mathcal{L}_m\big). \tag{23}$$

Conditioning the expected term in Eq. (22) on the true infectivity status of subject w gives:

$$\mathbb{E}\big[FP|w \in \Omega^I(x_m^p)\big] = \mathbb{E}\big[FP|I_w = 0, w \in \Omega^I(x_m^p)\big]P\big(I_s = 0|w \in \Omega^I(x_m^p)\big), \tag{24}$$

where Eq. (24) is obtained by noting that if subject is truly positive ($I_w = 1$), a false positive cannot occur. Given that the subject is tested individually, a false positive will occur with probability $1 - Sp$, that is

$$\mathbb{E}\big[FP|I_w = 0, w \in \Omega^I(x_m^p)\big] = 1 - Sp.$$

By noting that

$$P\big(I_s = 0|w \in \Omega^I(x_m^p)\big) = 1 - r_m,$$

Eq. (24) is given by:

$$\mathbb{E}\big[FP|w \in \Omega^I(x_m^p)\big] = (1 - Sp)(1 - r_m). \tag{25}$$

Similarly, the expected term in Eq. (23) can be written as:

$$\begin{aligned}\mathbb{E}\big[FP|w \in \Omega^G(x_m^p)\big] =& \mathbb{E}\big[FP|I_w = 0, w \in \Omega^G(x_m^p)\big]P\big(I_w = 0|w \in \Omega^G(x_m^p)\big)\\ =& \mathbb{E}\big[FP|I_w = 0, w \in \Omega^G(x_m^p)\big](1 - r_m).\end{aligned} \tag{26}$$

Given that the subject is tested via Dorfman testing, then a false positive will occur when the outcome of group testing is positive and the outcome of the subsequent individual testing is positive. Since, at the first stage, the outcome of group testing depends on the infectivity status of all subjects in the same testing, let I_w^G denote a binary variable indicating the true infectivity status of the remaining subjects of the group (i.e., subjects that are grouped with subject w while excluding subject w), with a value of 1 representing that at least one of the remaining subjects is truly positive and a value of 0 otherwise. Then, further conditioning $\mathbb{E}\big[FP|I_w = 0, w \in \Omega^G(x_m^p)\big]$ on I_w^G gives:

$$\begin{aligned}\mathbb{E}\big[FP|I_w = 0, w \in \Omega^G(x_m^p)\big] =& \mathbb{E}\big[FP|I_w = 0, I_w^G = 0, w \in \Omega^G(x_m^p)\big]P\big(I_w^G = 0|I_w = 0, w \in \Omega^G(x_m^p)\big)\\ &+ \mathbb{E}\big[FP|I_w = 0, I_w^G = 1, w \in \Omega^G(x_m^p)\big]P\big(I_w^G = 1|I_w = 0, w \in \Omega^G(x_m^p)\big)\end{aligned} \tag{27}$$

First, when $I_w^G = 0$, a false positive occurs when the test outcome of group testing is positive (with probability of $1 - Sp$) and the test out of the subsequent individual testing of subject w is positive (with probability of $1 - Sp$), which leads to:

$$\mathbb{E}\big[FP|I_w = 0, I_w^G = 0, w \in \Omega^G(x_m^p)\big] = (1 - Sp)^2. \tag{28}$$

On the other hand, when $I_w^G = 1$, a false positive occurs when the test outcome of group testing is positive (with probability of Se) and the test out of the subsequent individual testing of subject w is positive (with probability of $1 - Sp$), which leads to:

$$\mathbb{E}\big[FP|I_w = 0, I_w^G = 1, w \in \Omega^G(x_m^p)\big] = Se(1 - Sp). \tag{29}$$

Substituting Eqs. (28) and (29) into (27) and by noting that

$$P\big(I_w^G = 0|I_w = 0, w \in \Omega^G(x_m^p)\big) = (1 - r_m)^{n_m^p - 1}$$

and

$$P\big(I_w^G = 1|I_w = 0, w \in \Omega^G(x_m^p)\big) = 1 - (1 - r_m)^{n_m^p - 1}$$

gives:

$$\mathbb{E}\big[FP|I_w = 0, w \in \Omega^G(x_m^p)\big] = (1 - Sp)^2(1 - r_m)^{n_m^p - 1} + Se(1 - Sp)\big[1 - (1 - r_m)^{n_m^p - 1}\big]. \tag{30}$$

Substituting Eq. (30) into Eq. (26) gives:

$$\begin{aligned}\mathbb{E}\big[FP|w \in \Omega^G(x_m^p)\big] =& \Big[(1 - Sp)^2(1 - r_m)^{n_m^p - 1} + Se(1 - Sp)\big[1 - (1 - r_m)^{n_m^p - 1}\big]\Big](1 - r_m) \\ =& Se(1 - Sp)(1 - r_m) - (1 - Sp)(Se + Sp - 1)(1 - r_m)^{n_m^p}. \end{aligned} \tag{31}$$

Lastly, substituting Eqs. (25) and (31) into Eqs. (15) and (16) and by noting that the probability terms are equal to y_m^p and $1 - y_m^p$ gives:

$$\mathbb{E}_m^p\big[FP\big] = (1 - Sp)(1 - r_m)y_m^p + \Big[Se(1 - Sp)(1 - r_m) - (1 - Sp)(Se + Sp - 1)(1 - r_m)^{n_m^p}\Big](1 - y_m^p). \tag{32}$$

$\underline{\mathbb{E}_m^p[TC]}$: Similarly, we first condition on whether the subject is individually tested or tested via group testing, which leads to:

$$\mathbb{E}_m^p\big[TC\big] = \mathbb{E}\big[TC|w \in \Omega^I(x_m^p)\big]P\big(w \in \Omega^I(x_m^p)|w \in \mathcal{L}_m\big) \tag{33}$$

$$+ \mathbb{E}\big[TC|w \in \Omega^G(x_m^p)\big]P\big(w \in \Omega^G(x_m^p)|w \in \mathcal{L}_m\big). \tag{34}$$

Given that the subject is tested individually, the expected number of test is equal to 1, that is

$$\mathbb{E}\big[TC|w \in \Omega^I(x_m^p)\big] = 1. \tag{35}$$

Since the expected number of test is depend on the infectious status of the group, let I^{G_m} be a binary random variable representing the true infectivity status of the subjects in the group with w (including m), with a value of 0 indicating all subjects are negative and 1 otherwise. Conditioning

$\mathbb{E}\big[TC|w \in \Omega^G(x_m^p)\big]$ on I^{G_m} gives:

$$\begin{aligned}\mathbb{E}\big[TC|w \in \Omega^G(x_m^p)\big] =& \mathbb{E}\big[TC|I^{G_m}=1, w \in \Omega^G(x_m^p)\big]P\big(I^{G_m}=1|w \in \Omega^I(x_m^p), w \in \mathcal{L}_m\big) \\ &+ \mathbb{E}\big[TC|I^{G_m}=0, w \in \Omega^G(x_m^p)\big]P\big(I^{G_m}=0|w \in \Omega^I(x_m^p), w \in \mathcal{L}_m\big). \quad (36)\end{aligned}$$

To obtain the expression for the expected terms in Eq. (36), we first note that if the group testing outcome is negative, then the expected number of tested per subject is $1/n_m^p$. On the other hand, if the group testing outcome is positive, then each subject in the group needs a subsequent test, which leads to total $1+n_m^p$ tests and $1+1/n_m^p$ test per subject. Given this, the expected terms in Eq. (36) can be written as:

$$\mathbb{E}\big[TC|I^{G_m}=1, w \in \Omega^G(x_m^p)\big] = (1-Se)\frac{1}{n_m^p} + Se(1+\frac{1}{n_m^p}), \quad (37)$$

and

$$\mathbb{E}\big[TC|I^{G_m}=0, w \in \Omega^G(x_m^p)\big] = Sp\frac{1}{n_m^p} + (1-Sp)(1+\frac{1}{n_m^p}). \quad (38)$$

Substituting Eqs. (37) and (38) into Eq. (38) and noting that

$$P\big(I^{G_m}=1|w \in \Omega^I(x_m^p), w \in \mathcal{L}_m\big) = 1-(1-r_m)^{n_m^p},$$

$$P\big(I^{G_m}=0|w \in \Omega^I(x_m^p), w \in \mathcal{L}_m\big) = (1-r_m)^{n_m^p}$$

gives:

$$\mathbb{E}\big[TC|w \in \Omega^G(x_m^p)\big] = \frac{1}{n_m^p} + Se - (Se+Sp-1)(1-r_m)^{n_m^p}. \quad (39)$$

Lastly, substituting Eqs.(39) and Eq.(35) into Eqs. (33) and (34) gives:

$$\mathbb{E}_m^p\big[TC\big] = y_m^p + \left[\frac{1}{n_m^p} + Se - (Se+Sp-1)(1-r_m)^{n_m^p}\right](1-y_m^p). \quad (40)$$

B.2 Non-proactive Testing

$\underline{\mathbb{E}_m^{\overline{p}}[FN]}$: To obtain the expression of performance measures for non-proactive testing subset, we first note that if the subject is not proactively tested, the subject will be reactively tested only if the subject shows symptoms. Given this, let S_w be a binary random variable representing the symptomatic status of the subject w, with a value of 1 indicating a symptomatic case and 0 otherwise. Conditioning $\mathbb{E}_m^{\overline{p}}[FN]$ on the symptomatic status of subject w gives:

$$\begin{aligned}\mathbb{E}_m^{\overline{p}}[FN] =& \mathbb{E}\big[FN|S_w=0, w \in \mathcal{L}_m\big]P\big(S_w=0|w \in \mathcal{L}_m\big) \quad (41)\\ &+ \mathbb{E}\big[FN|S_w=1, w \in \mathcal{L}_m\big]P\big(S_w=1|w \in \mathcal{L}_m\big) \quad (42)\end{aligned}$$

Further conditioning the expected term in Eq. (41) on the true infectious status of w leads to:

$$\begin{aligned}\mathbb{E}\big[FN|S_w=0, w \in \mathcal{L}_m\big] =& \mathbb{E}\big[FN|I_w=0, S_w=0, w \in \mathcal{L}_m\big]P\big(I_w=0|S_w=0, w \in \mathcal{L}_m\big) \\ &+ \mathbb{E}\big[FN|I_w=1, S_w=0, w \in \mathcal{L}_m\big]P\big(I_w=1|S_w=0, w \in \mathcal{L}_m\big) \\ =& \mathbb{E}\big[FN|I_w=1, S_w=0, w \in \mathcal{L}_m\big]P\big(I_w=1|S_w=0, w \in \mathcal{L}_m\big) \quad (43)\end{aligned}$$

Given that the subject is not symptomatic, the subject will not be tested and will be classified based on the classification policy for non-symptomatic cases (i.e., $z_m^{\bar{s}}$). In particular, $z_m^{\bar{s}} = 0$ indicating that non-symptomatic cases are classified as negative and $z_m^{\bar{s}} = 1$ otherwise. As such, the expected term in Eq.(43) can be written as:

$$\mathbb{E}\big[FN|I_w = 1, S_w = 0, w \in \mathcal{L}_m\big] = 1 - z_m^{\bar{s}},$$

where the last equation is obtained by noting that a false negative will only occur when the non-symptomatic case is classified as negative. Utilizing the expression in Eq. (6), the probability term in Eq.(43) can be obtained by Bayes' rule:

$$\begin{aligned} P\big(I_w = 1|S_w = 0, w \in \mathcal{L}_m\big) =& \frac{P\big(S_w = 0|I_w = 1, w \in \mathcal{L}_m\big)P\big(I_w = 1, w \in \mathcal{L}_m\big)}{P\big(S_w = 0\big)} \\ =& \frac{(1 - s_m)(1 + \alpha_m f_m)r_m}{1 - \alpha_m f_m - s_m r_m + \alpha_m s_m f_m r_m}. \end{aligned} \tag{44}$$

To facilitate the presentation, we define H_m as:

$$H_m = \frac{(1 - s_m)(1 + \alpha_m f_m)r_m}{1 - \alpha_m f_m - s_m r_m + \alpha_m s_m f_m r_m}. \tag{45}$$

As such, Eq. (43) can be written as:

$$\mathbb{E}\big[FN|S_w = 0, w \in \mathcal{L}_m\big] = (1 - z_m^{\bar{s}})H_m \tag{46}$$

Next, let $\Omega(x_m^r)$ denote the subset of subjects in category m that is reactively tested. Conditioning the expected term in Eq. (42) on whether the subject is individually tested, tested via group testing, or not tested gives:

$$\begin{aligned} \mathbb{E}\big[FN|S_w = 1, w \in \mathcal{L}_m\big] =& \mathbb{E}\big[FN|w \in \Omega^I(x_m^r), S_w = 1, w \in \mathcal{L}_m\big]P\big(w \in \Omega^I(x_m^r)|S_w = 1, w \in \mathcal{L}_m\big) && (47) \\ &+ \mathbb{E}\big[FN|w \in \Omega^G(x_m^r), S_w = 1, w \in \mathcal{L}_m\big]P\big(w \in \Omega^G(x_m^r)|S_w = 1, w \in \mathcal{L}_m\big) && (48) \\ &+ \mathbb{E}\big[FN|w \notin \Omega(x_m^r), S_w = 1, w \in \mathcal{L}_m\big]P\big(w \notin \Omega(x_m^r)|S_w = 1, w \in \mathcal{L}_m\big) && (49) \end{aligned}$$

In what follow, we derive the expression for the expected terms in Eqs. (47), (48) and (49) respectively. By conditioning on the true infectivity status, the expected term in Eq. (47) can be written as:

$$\begin{aligned} \mathbb{E}\big[FN|w \in \Omega^I(x_m^r), S_w = 1, w \in \mathcal{L}_m\big] =& \mathbb{E}\big[FN|I_w = 1, w \in \Omega^I(x_m^r), S_w = 1, w \in \mathcal{L}_m\big] \\ & \times P\big(I_w = 1|w \in \Omega^I(x_m^r), S_w = 1, w \in \mathcal{L}_m\big) \\ =& (1 - Se)P\big(I_w = 1|S_w = 1, w \in \mathcal{L}_m\big), \end{aligned} \tag{50}$$

where the last equation is obtained by noting that a false negative will only occur if subject is infected and a false negative will occur with probability $1 - Se$. According to the Bayes' rule, the

probability term in last equation is given by:

$$P\big(I_w = 1|S_w = 1, w \in \mathcal{L}_m\big) = \frac{P\big(S_w = 1|I_w = 1, w \in \mathcal{L}_m\big)P\big(I_w = 1, w \in \mathcal{L}_m\big)}{P\big(S_w = 1, w \in \mathcal{L}_m\big)}$$
$$= \frac{\alpha_m f_m r_m + s_m r_m - \alpha_m s_m f_m r_m}{\alpha_m f_m + s_m r_m - \alpha_m s_m f_m r_m}.$$

To facilitate the presentation, we define J_m as:

$$J_m = \frac{\alpha_m f_m r_m + s_m r_m - \alpha_m s_m f_m r_m}{\alpha_m f_m + s_m r_m - \alpha_m s_m f_m r_m}. \tag{51}$$

Given this, Eq. (50) can be written as:

$$\mathbb{E}\big[FN|w \in \Omega^I(x_m^r), S_w = 1, w \in \mathcal{L}_m\big] = (1 - Se)J_m. \tag{52}$$

Similarly, by conditioning on the true infectivity status, the expected term in Eq. (48) can be written as:

$$\mathbb{E}\big[FN|w \in \Omega^G(x_m^r), S_w = 1, w \in \mathcal{L}_m\big] = \mathbb{E}\big[FN|I_w = 1, w \in \Omega^G(x_m^r), S_w = 1, w \in \mathcal{L}_m\big]$$
$$\times P\big(I_w = 1|w \in \Omega^G(x_m^r), S_w = 1, w \in \mathcal{L}_m\big)$$
$$= (1 - Se^2)P\big(I_w = 1|S_w = 1, w \in \mathcal{L}_m\big),$$

where the last equation is obtained by first noting that a false negative will only occur if subject is tested. Then, given the subject is tested via group testing, a false negative will occur in two scenarios: (i) if the outcome of the group testing is negative (with probability $1 - Se$) or (ii) if the outcome of the group testing is positive (with probability Se) and the outcome of the subsequent individual testing is negative (with probability $1 - Se$), which leads to the probability of false negative be $1 - Se^2$. Next, substituting the probability term from (51) into the last equation gives:

$$\mathbb{E}\big[FN|w \in \Omega^G(x_m^r), S_w = 1, w \in \mathcal{L}_m\big] = (1 - Se^2)J_m. \tag{53}$$

Similarly, by conditioning on the true infectivity status, the expected term in Eq. (49) can be written as:

$$\mathbb{E}\big[FN|w \notin \Omega(x_m^r), S_w = 1, w \in \mathcal{L}_m\big] = \mathbb{E}\big[FN|I_w = 1, w \notin \Omega(x_m^r), S_w = 1, w \in \mathcal{L}_m\big]$$
$$\times P\big(I_w = 1|w \notin \Omega(x_m^r), S_w = 1, w \in \mathcal{L}_m\big)$$
$$= (1 - z_m^s)P\big(I_w = 1|S_w = 1, w \in \mathcal{L}_m\big),$$

where the equation is obtained by noting that if the symptomatic subject w is not tested, a false negative will only occur if the subject is classified as negative (i.e., when $z^s = 0$). Since the probability term in the last equation is equal to J_m, the equation is given by:

$$\mathbb{E}\big[FN|w \notin \Omega(x_m^r), S_w = 1, w \in \mathcal{L}_m\big] = (1 - z_m^s)J_m. \tag{54}$$

Substituting Eqs.(52), (53) and (54) into Eqs. (47), (48) and (49) gives:

$$\mathbb{E}\big[FN|S_w = 1, w \in \mathcal{L}_m\big] = \big[(1 - Se)J_m y_m^r + (1 - Se^2)J_m(1 - y_m^r)\big]x_m^r + (1 - z_m^s)J_m(1 - x_m^r) \tag{55}$$

Lastly, substituting Eqs. (46) and (55) into Eqs. (41) and (42) gives:

$$
\begin{aligned}
\mathbb{E}^{\overline{p}}_m[FN] =&(1-z^{\overline{s}}_m)H_m P\big(S_w=0|w\in\mathcal{L}_m\big)\\
&+\Big[[(1-Se)J_m y^r_m+(1-Se^2)J_m(1-y^r_m)]x^r_m+(1-z^s_m)J_m(1-x^r_m)\Big]P\big(S_w=1|w\in\mathcal{L}_m\big)\\
=&(1-z^{\overline{s}}_m)H_m\big[1-P\big(S_w=1|w\in\mathcal{L}_m\big)\big]\\
&+\Big[[(1-Se)J_m y^r_m+(1-Se^2)J_m(1-y^r_m)]x^r_m+(1-z^s_m)J_m(1-x^r_m)\Big]P\big(S_w=1|w\in\mathcal{L}_m\big)\\
=&(1-z^{\overline{s}}_m)H_m(1-l_m)+\Big[[(1-Se)J_m y^r_m+(1-Se^2)J_m(1-y^r_m)]x^r_m+(1-z^s_m)J_m(1-x^r_m)\Big]l_m
\end{aligned}
$$

$\underline{\mathbb{E}^{\overline{p}}_m[FP]}$: Similar procedure can be adopted for derivation of false positives. First, by conditioning $\mathbb{E}^{\overline{p}}_m[FP]$ on the symptomatic status of subject w, we have:

$$
\begin{aligned}
\mathbb{E}^{\overline{p}}_m[FP] =&\mathbb{E}\big[FP|S_w=0,w\in\mathcal{L}_m\big]P\big(S_w=0|w\in\mathcal{L}_m\big) &(56)\\
&+\mathbb{E}\big[FP|S_w=1,w\in\mathcal{L}_m\big]P\big(S_w=1|w\in\mathcal{L}_m\big) &(57)
\end{aligned}
$$

Further conditioning the expected term in Eq. (56) on the true infectious status of w leads to:

$$
\begin{aligned}
\mathbb{E}\big[FP|S_w=0,w\in\mathcal{L}_m\big] =&\mathbb{E}\big[FP|I_w=0,S_w=0,w\in\mathcal{L}_m\big]P\big(I_w=0|S_w=0,w\in\mathcal{L}_m\big)\\
&+\mathbb{E}\big[FP|I_w=1,S_w=0,w\in\mathcal{L}_m\big]P\big(I_w=1|S_w=0,w\in\mathcal{L}_m\big)\\
=&\mathbb{E}\big[FP|I_w=0,S_w=0,w\in\mathcal{L}_m\big]P\big(I_w=0|S_w=0,w\in\mathcal{L}_m\big) &(58)
\end{aligned}
$$

The last equation is obtained by noting that if the subject is infected, a false positive will not occur. Given that the subject is not symptomatic, the subject will not be tested and will be classified based on the given classification policy for non-symptomatic cases (i.e., $z^{\overline{s}}_m$). In particular, $z^{\overline{s}}_m=1$ indicating that non-symtomatic cases are classified as positive and $z^{\overline{s}}_m=0$ otherwise. As such, the expected term in Eq.(58) can be written as:

$$
\mathbb{E}\big[FP|I_w=0,S_w=0,w\in\mathcal{L}_m\big]=z^{\overline{s}}_m,
$$

where the last equation is obtained by noting that a false positive will only occur when the non-symptomatic case is classified as negative. Then, by noting that the probability term in Eq. (58) is given by Eq. (45), Eq. (58) can be written as:

$$
\mathbb{E}\big[FP|S_w=0,w\in\mathcal{L}_m\big]=z^{\overline{s}}_m(1-H_m). \tag{59}
$$

Next, conditioning the expected term in Eq. (57) on whether the subject is individually tested, tested via group testing, or not tested gives:

$$
\begin{aligned}
\mathbb{E}\big[FP|S_w=1,w\in\mathcal{L}_m\big] =&\mathbb{E}\big[FP|w\in\Omega^I(x^r_m),S_w=1,w\in\mathcal{L}_m\big]P\big(w\in\Omega^I(x^r_m)|S_w=1,w\in\mathcal{L}_m\big) &(60)\\
&+\mathbb{E}\big[FP|w\in\Omega^G(x^r_m),S_w=1,w\in\mathcal{L}_m\big]P\big(w\in\Omega^G(x^r_m)|S_w=1,w\in\mathcal{L}_m\big) &(61)\\
&+\mathbb{E}\big[FP|w\notin\Omega(x^r_m),S_w=1,w\in\mathcal{L}_m\big]P\big(w\notin\Omega(x^r_m)|S_w=1,w\in\mathcal{L}_m\big) &(62)
\end{aligned}
$$

In what follows, we derive the expression for the expected terms in Eqs. (60), (61) and (62) respectively. By conditioning on the true infectivity status, the expected term in Eq. (60) can be written as:

$$
\begin{aligned}
\mathbb{E}\big[FP|w\in\Omega^I(x^r_m),S_w=1,w\in\mathcal{L}_m\big] =&\mathbb{E}\big[FP|I_w=0,w\in\Omega^I(x^r_m),S_w=1,w\in\mathcal{L}_m\big]\\
&\times P\big(I_w=0|w\in\Omega^I(x^r_m),S_w=1,w\in\mathcal{L}_m\big)\\
=&(1-Sp)P\big(I_w=0|S_w=1,w\in\mathcal{L}_m\big), &(63)
\end{aligned}
$$

where the equation is obtained by noting that a false positive will only occur if the subject is not infected. If tested, the probability of having false positives is $1 - Sp$. According to Eq. (45), the probability term in Eq. (63) is given by:

$$P\big(I_w = 0|S_w = 1, w \in \mathcal{L}_m\big) = 1 - P\big(I_w = 1|S_w = 1, w \in \mathcal{L}_m\big) = 1 - J_m. \tag{64}$$

Consequently, Eq. (63) can be written as:

$$\mathbb{E}\big[FP|w \in \Omega^I(x_m^r), S_w = 0, w \in \mathcal{L}_m\big] = (1 - Sp)(1 - J_m). \tag{65}$$

Similarly, by conditioning on the true infectivity status, the expected term in Eq. (61) can be written as:

$$\begin{aligned}\mathbb{E}\big[FP|w \in \Omega^G(x_m^r), S_w = 1, w \in \mathcal{L}_m\big] = \mathbb{E}\big[FP|I_w = 0, w \in \Omega^G(x_m^r), S_w = 1, w \in \mathcal{L}_m\big]\\ \times P\big(I_w = 0|w \in \Omega^G(x_m^r), S_w = 1, w \in \mathcal{L}_m\big)\end{aligned} \tag{66}$$

Given that the subject is infected and tested, a false positive will occur if the group testing outcome is positive and the subsequent individual testing is positive. Therefore, the test result depends on the true infectivity status of the remaining subjects in the group with subject w. Then, further conditioning $\mathbb{E}\big[FP|I_w = 0, w \in \Omega^G(x_m^r), S_w = 1, w \in \mathcal{L}_m\big]$ on the true infectivity status of the ramining subjects of the group (I_w^G) gives:

$$\mathbb{E}\big[FP|I_w = 0, s_w = 1, w \in \Omega^G(x_m^p)\big] = \mathbb{E}\big[FP|I_w = 0, s_w = 1, I_w^G = 0, w \in \Omega^G(x_m^p)\big] \tag{67}$$

$$\times P\big(I_w^G = 0|I_w = 0, s_w = 1, w \in \Omega^G(x_m^p)\big)$$

$$+ \mathbb{E}\big[FP|I_w = 0, I_w^G = 1, w \in \Omega^G(x_m^p)\big] \tag{68}$$

$$\times P\big(I_w^G = 1|I_w = 0, s_w = 1, w \in \Omega^G(x_m^p)\big).$$

When $I_s^G = 0$, a false positive will occur if the outcome of the group testing is falsely classifying the group as positive (with the probability of $1 - Sp$) and the outcome of the subsequent individual testing is positive (with the probability of $1 - Sp$), which leads to:

$$\mathbb{E}\big[FP|I_w = 0, s_w = 1, I_w^G = 0, w \in \Omega^G(x_m^p)\big] = (1 - Sp)^2. \tag{69}$$

On the other hand, when $I_s^G = 1$, a false positive will occur if the outcome of the group testing is correctly classifying the group as positive (with the probability of Se)and the outcome of the subsequent individual testing is positive (with the probability of $1 - Sp$), which leads to:

$$\mathbb{E}\big[FP|I_w = 0, s_w = 1, I_w^G = 0, w \in \Omega^G(x_m^p)\big] = Se(1 - Sp). \tag{70}$$

Noting that the infectivity status of the remaining subjects with subject w (I_G^w) is independent on the infectivity and symptomatic status of subject w, the probability terms are given by:

$$P\big(I_w^G = 0|I_w = 0, s_w = 1, w \in \Omega^G(x_m^p)\big) = P\big(I_w^G = 0|w \in \Omega^G(x_m^p)\big) = (1 - r_w)^{n_m^p - 1}.$$

$$P\big(I_w^G = 1|I_w = 0, s_w = 1, w \in \Omega^G(x_m^p)\big) = P\big(I_w^G = 1|w \in \Omega^G(x_m^p)\big) = 1 - (1 - r_w)^{n_m^p - 1}.$$

Substituting the probability terms and Eqs. (69) and (70) into Eqs. (67) and (68) gives:

$$\mathbb{E}\big[FP|I_w = 0, s_w = 1, w \in \Omega^G(x_m^p)\big] = (1 - Sp)^2(1 - r_w)^{n_m^p - 1} + Se(1 - Sp)\big[1 - (1 - r_w)^{n_m^p - 1}\big]. \tag{71}$$

Substituting the last equation into Eq. (66) gives:

$$\mathbb{E}\big[FP|w \in \Omega^G(x_m^r), S_w = 1, w \in \mathcal{L}_m\big] = \Big[(1-Sp)^2(1-r_w)^{n_m^p-1}+Se(1-Sp)\big[1-(1-r_w)^{n_m^p-1}\big]\Big](1-J_m). \tag{72}$$

Similarly, by conditioning on the true infectivity status, the expected term in Eq.(62) can be written as:

$$\begin{aligned}\mathbb{E}\big[FP|w\notin\Omega(x^r_m),S_w=1,w\in\mathcal{L}_m\big]=&\,\mathbb{E}\big[FP|I_w=0,w\notin\Omega(x^r_m),S_w=1,w\in\mathcal{L}_m\big]\\&\times P\big(I_w=0|w\notin\Omega(x^r_m),S_w=1,w\in\mathcal{L}_m\big)\\=&z^s_m\cdot P\big(I_w=0|S_w=1,w\in\mathcal{L}_m\big),\end{aligned}$$

where the equation is obtained by noting that if the symptomatic subject w is not tested, a false positive will only occur if the subject is classified as positive (i.e., when $z^s_m=1$). Since the probability term in the last equation is equal to J_m, the equation is given by:

$$\mathbb{E}\big[FP|w\notin\Omega(x^r_m),S_w=1,w\in\mathcal{L}_m\big]=z^s_m(1-J_m).\tag{73}$$

Substituting Eqs.(65), (72) and (73) into Eqs. (60), (61) and (62) gives:

$$\begin{aligned}\mathbb{E}\big[FP|S_w=1,w\in\mathcal{L}_m\big]=&(1-Sp)(1-J_m)x^r_my^r_m+z^s_m(1-J_m)(1-x^r_m)\\&+\Big[(1-Sp)^2(1-r_w)^{n^r_m-1}+Se(1-Sp)\big[1-(1-r_w)^{n^r_m-1}\big]\Big](1-J_m)x^r_m(1-y^r_m).\end{aligned}\tag{74}$$

Lastly, substituting Eqs. (59) and (74) into Eqs. (56) and (57) gives:

$$\begin{aligned}\mathbb{E}^{\overline{p}}_m[FP]=&z^{\overline{s}}_m(1-H_m)(1-l_m)+\Big[(1-Sp)(1-J_m)x^r_my^r_m+z^s_m(1-J_m)(1-x^r_m)\\&+\big[(1-Sp)^2(1-r_w)^{n^r_m-1}+Se(1-Sp)\big[1-(1-r_w)^{n^r_m-1}\big]\big](1-J_m)x^r_m(1-y^r_m)\Big]l_m.\end{aligned}\tag{75}$$

<u>$\mathbb{E}^{\overline{p}}_m[TC]$</u>: Similarly, we first condition on the symptomatic status of subject w gives:

$$\begin{aligned}\mathbb{E}^{\overline{p}}_m\big[TC\big]=&\mathbb{E}\big[TC|s_w=1\big]P\big(s_w=1|w\in\mathcal{L}_m\big)+\mathbb{E}\big[TC|s_w=0\big]P\big(s_w=0|w\in\mathcal{L}_m\big)\\=&\mathbb{E}\big[TC|s_w=1\big]P\big(s_w=1|w\in\mathcal{L}_m\big).\end{aligned}\tag{76}$$

The last equation is obtained by noting that tests are only performed for symptomatic subjects. Further conditioning the expected term in Eq. (76) on whether the subject is individually tested, tested via group testing or not tested leads to:

$$\begin{aligned}\mathbb{E}\big[TC|s_w=1\big]=&\mathbb{E}\big[TC|w\in\Omega^I(x^r_m),s_w=1\big]P\big(w\in\Omega^I(x^r_m)\big)\\&+\mathbb{E}\big[TC|w\in\Omega^G(x^r_m),s_w=1\big]P\big(w\in\Omega^G(x^r_m)\big)\\&+\mathbb{E}\big[TC|w\notin\Omega(x^r_m),s_w=1\big]P\big(w\notin\Omega(x^r_m)\big)\\=&\mathbb{E}\big[TC|w\in\Omega^I(x^r_m),s_w=1\big]P\big(w\in\Omega^I(x^r_m)\big)&(77)\\&+\mathbb{E}\big[TC|w\in\Omega^G(x^r_m),s_w=1\big]P\big(w\in\Omega^G(x^r_m)\big).&(78)\end{aligned}$$

Given that the subject w is tested individually, the expected number of test is equal to 1, that is

$$\mathbb{E}\big[TC|w\in\Omega^I(x^r_m),s_w=1\big]=1.\tag{79}$$

Next, conditioning on the infectivity status of the testing group of subject w (i.e., I^{G_w}) leads to:

$$\begin{aligned}\mathbb{E}\big[TC|w\in\Omega^G(x_m^r)\big] =&\mathbb{E}\big[TC|I^{G_m}=1,s_w=1,w\in\Omega^G(x_m^r)\big]P\big(I^{G_m}=1|s_w=1,w\in\Omega^G(x_m^r)\big)\\&+\mathbb{E}\big[TC|I^{G_m}=0,s_w=1,w\in\Omega^G(x_m^r)\big]P\big(I^{G_m}=0|s_w=1,w\in\Omega^G(x_m^r)\big).\end{aligned}\tag{80}$$

Given that the group testing outcome is negative, the expected number of tested per subject is $1/n_m^r$. On the other hand, if the group testing outcome is positive, then each subject in the group needs a subsequent test, which leads to total $1+n_m^r$ tests and $1+1/n_m^r$ tests per subject. Given this, the expected terms in Eq. (80) can be written as:

$$\mathbb{E}\big[TC|I^{G_m}=1,s_w=1,w\in\Omega^G(x_m^r)\big]=(1-Se)\frac{1}{n_m^r}+Se(1+\frac{1}{n_m^r})\tag{81}$$

and

$$\mathbb{E}\big[TC|I^{G_m}=0,s_w=1,w\in\Omega^G(x_m^r)\big]=Sp\frac{1}{n_m^r}+(1-Sp)(1+\frac{1}{n_m^r}).\tag{82}$$

Next, we derive the expressions of the probability terms in Eq. (80). Based on Bayes' rule, the probability term $P\big(I^{G_m}=1|s_w=1,w\in\Omega^G(x_m^r)\big)$ is given by:

$$\begin{aligned}P\big(I^{G_m}=1|s_w=1,w\in\Omega^G(x_m^r)\big)=&\frac{P\big(s_w=1|I^{G_m}=1,w\in\Omega^G(x_m^r)\big)P\big(I^{G_m}=1,w\in\Omega^G(x_m^r)\big)}{P\big(s_w=1|w\in\Omega^G(x_m^r)\big)}\\=&\frac{P\big(s_w=1|I^{G_m}=1,w\in\Omega^G(x_m^r)\big)(1-(1-r_m)^{n_m^r})}{l_m},\end{aligned}\tag{83}$$

Conditioning the probability term in the last equation on the true infectivity status of the subject w gives:

$$\begin{aligned}P\big(s_w=1|I^{G_m}=1,w\in\Omega^G(x_m^r)\big)=&P\big(s_w=1|I_w=1,I^{G_m}=1,w\in\Omega^I(x_m^r)\big)P\big(I_w=1|I^{G_m}=1,w\in\Omega^G(x_m^r)\big)\\&+P\big(s_w=1|I_w=0,I^{G_m}=1,w\in\Omega^G(x_m^r)\big)P\big(I_w=0|I^{G_m}=1,w\in\Omega^G(x_m^r)\big)\\=&(\alpha_m f_m-\alpha_m s_m f_m+s_m)P\big(I_w=1|I^{G_m}=1,w\in\Omega^G(x_m^r)\big)\\&+f_m\alpha_m P\big(I_w=0|I^{G_m}=1,w\in\Omega^I(x_m^r)\big)\end{aligned}\tag{84}$$

The probability terms in last equation, according to Bayes' rule, can be written as:

$$\begin{aligned}P\big(I_w=1|I^{G_m}=1,w\in\Omega^I(x_m^r)\big)=&\frac{P\big(I^{G_m}=1|I_w=1,w\in\Omega^I(x_m^r)\big)P\big(I_w=1|w\in\Omega^I(x_m^r)\big)}{P\big(I^{G_m}=1|w\in\Omega^I(x_m^r)\big)}\\=&\frac{1\cdot r_m}{\big(1-(1-r_m)^{n_m^r}\big)},\end{aligned}\tag{85}$$

and

$$\begin{aligned}P\big(I_w=0|I^{G_m}=1,w\in\Omega^I(x_m^r)\big)=&\frac{P\big(I^{G_m}=1|I_w=0,w\in\Omega^I(x_m^r)\big)P\big(I_w=0|w\in\Omega^I(x_m^r)\big)}{P\big(I^{G_m}=1|w\in\Omega^I(x_m^r)\big)}\\=&\frac{\big(1-(1-r_m)^{n_m^r-1}\big)\cdot(1-r_m)}{\big(1-(1-r_m)^{n_m^r}\big)}.\end{aligned}\tag{86}$$

Substituting Eqs. (85) and (86) into Eq. (84) gives:

$$P\big(s_w = 1 | I^{G_m} = 1, w \in \Omega^G(x_m^r)\big) = \frac{(\alpha_m f_m - \alpha_m s_m f_m + s_m) \cdot r_m}{\big(1 - (1 - r_m)^{n_m^r}\big)} + \frac{f_m \alpha_m \big(1 - (1 - r_m)^{n_m^r - 1}\big) \cdot (1 - r_m)}{\big(1 - (1 - r_m)^{n_m^r}\big)} \tag{87}$$

Substituting Eq. (87) into Eq. (83) gives:

$$P\big(I^{G_m} = 1 | s_w = 1, w \in \Omega^G(x_m^r)\big) = \frac{(\alpha_m f_m - \alpha_m s_m f_m + s_m) \cdot r_m + f_m \alpha_m \big(1 - (1 - r_m)^{n_m^r - 1}\big) \cdot (1 - r_m)}{l_m}. \tag{88}$$

Similarly, the second probability term on the right side of Eq. (80) is given by:

$$\begin{aligned} P\big(I^{G_m} = 0 | s_w = 1, w \in \Omega^G(x_m^r)\big) =& \frac{P\big(s_w = 1 | I^{G_m} = 0, w \in \Omega^G(x_m^r)\big) P\big(I^{G_m} = 0, w \in \Omega^G(x_m^r)\big)}{P\big(s_w = 1 | w \in \Omega^G(x_m^r)\big)} \\ =& \frac{P\big(s_w = 1 | I^{G_m} = 0, w \in \Omega^G(x_m^r)\big)(1 - r_m)^{n_m^r}}{l_m}. \end{aligned} \tag{89}$$

The probability term in the last equation can be written as:

$$\begin{aligned} P\big(s_w = 1 | I^{G_m} = 0, w \in \Omega^G(x_m^r)\big) =& P\big(s_w = 1 | I_w = 1, I^{G_m} = 0, w \in \Omega^I(x_m^r)\big) P\big(I_w = 1 | I^{G_m} = 0, w \in \Omega^G(x_m^r)\big) \\ & + P\big(s_w = 1 | I_w = 0, I^{G_m} = 0, w \in \Omega^G(x_m^r)\big) P\big(I_w = 0 | I^{G_m} = 0, w \in \Omega^G(x_m^r)\big) \\ =& P\big(s_w = 1 | I_w = 0, I^{G_m} = 0, w \in \Omega^G(x_m^r)\big) P\big(I_w = 0 | I^{G_m} = 0, w \in \Omega^G(x_m^r)\big) \\ =& f_m \alpha_m. \end{aligned}$$

Substituting last equation into Eq. (89) gives:

$$P\big(I^{G_m} = 0 | s_w = 1, w \in \Omega^G(x_m^r)\big) = \frac{f_m \alpha_m (1 - r_m)^{n_m^r}}{l_m}. \tag{90}$$

Next, substituting probability terms Eqs. (88) and (90) with Eqs. (81) and (82) into Eqs. (80) and (77) gives:

$$\begin{aligned} \mathbb{E}[TC | w \in \Omega^G(x_m^r)] =& \Big[(1 - Se)\frac{1}{n_m^r} + Se(1 + \frac{1}{n_m^r})\Big] \frac{(\alpha_m f_m - \alpha_m s_m f_m + s_m) \cdot r_m + f_m \alpha_m \big(1 - (1 - r_m)^{n_m^r - 1}\big) \cdot (1 - r_m)}{l_m} \\ & + \Big[Sp\frac{1}{n_m^r} + (1 - Sp)(1 + \frac{1}{n_m^r})\Big] \frac{f_m \alpha_m (1 - r_m)^{n_m^r}}{l_m}. \end{aligned} \tag{91}$$

Then, substituting Eqs. (79) and (91) into (78) gives:

$$\begin{aligned} \mathbb{E}[TC | s_w = 1] =& \mathbb{E}[TC | w \in \Omega^I(x_m^r), s_w = 1] y_m^r + \mathbb{E}[TC | w \in \Omega^G(x_m^r), s_w = 1](1 - y_m^r) \\ =& y_m^r + (1 - y_m^r)\Big[[(1 - Se)\frac{1}{n_m^r} + Se(1 + \frac{1}{n_m^r})] \frac{(\alpha_m f_m (1 - f_m) + s_m) \cdot r_m + f_m \alpha_m \big(1 - (1 - r_m)^{n_m^r - 1}\big) \cdot (1 - r_m)}{l_m} \\ & + [Sp\frac{1}{n_m^r} + (1 - Sp)(1 + \frac{1}{n_m^r})] \frac{f_m \alpha_m (1 - r_m)^{n_m^r}}{l_m}\Big]. \end{aligned} \tag{92}$$

Lastly, substituting the last equation into Eq. (76) gives:

$$\begin{aligned} \mathbb{E}_m^{\overline{p}}[TC] =& l_m y_m^r x_m^r + \Big[[(1 - Se)\frac{1}{n_m^r} + Se(1 + \frac{1}{n_m^r})](\alpha_m f_m - \alpha_m s_m f_m + s_m) \cdot r_m + f_m \alpha_m \big(1 - (1 - r_m)^{n_m^r - 1}\big) \cdot (1 - r_m) \\ & + [Sp\frac{1}{n_m^r} + (1 - Sp)(1 + \frac{1}{n_m^r})] f_m \alpha_m (1 - r_m)^{n_m^r}\Big](1 - y_m^r) x_m^r. \end{aligned} \tag{93}$$

B.3 Derivation of the Optimization Model

In this section, we summarize all the performance expressions and substitute them into the objective function and budget constraint, resulting in the final form of the optimization model.

B.3.1 Objective Function

The objective function is given by (see Section 2 for more details):

$$
\begin{aligned}
&\sum_{m=1}^{M}\left[\Big(\lambda_m \mathbb{E}_m[FN] + (1-\lambda_m)\mathbb{E}_m[FP]\Big)\frac{p_m}{P_T}\right] \\
=&\sum_{m=1}^{M}\left[\lambda_m\left(\mathbb{E}_m^{p}\big[FN\big]\frac{p_m x_m^p}{P_T} + \mathbb{E}_m^{\overline{p}}\big[FN\big]\frac{p_m(1-x_m^p)}{P_T}\right) + (1-\lambda_m)\left(\mathbb{E}_m^{p}\big[FP\big]\frac{p_m x_m^p}{P_T} + \mathbb{E}_m^{\overline{p}}\big[FP\big]\frac{p_m(1-x_m^p)}{P_T}\right)\right] \\
=&\sum_{m=1}^{M}\left[\Big(\underbrace{\lambda_m \mathbb{E}_m^{p}\big[FN\big] + (1-\lambda_m)\mathbb{E}_m^{p}\big[FP\big]}_{(i)}\Big)\frac{p_m x_m^p}{P_T} + \Big(\underbrace{\lambda_m \mathbb{E}_m^{\overline{p}}\big[FN\big] + (1-\lambda_m)\mathbb{E}_m^{\overline{p}}\big[FP\big]}_{(ii)}\Big)\frac{p_m(1-x_m^p)}{P_T}\right].
\end{aligned}
\tag{94}
$$

The last equation is obtained by substituting Eq. (12) and (13) into **RP-MS**. The terms (i) and (ii) respectively represent the misclassifications arising from subjects who undergo proactive screening and subjects who are not tested via proactive screening (i.e., either tested by reactive screening or classified as untested). The expression of (i) and (ii) are given by

<u>(i) $\lambda_m \mathbb{E}_m^{p}\big[FN\big] + (1-\lambda_m)\mathbb{E}_m^{p}\big[FP\big]$</u>

$$
\begin{aligned}
&\lambda_m \mathbb{E}_m^{p}\big[FN\big] + (1-\lambda_m)\mathbb{E}_m^{p}\big[FP\big] \\
=&\lambda_m\Big[(1-Se)r_m y_m^p + (1-Se^2)r_m(1-y_m^p)\Big] \\
&+(1-\lambda_m)\Big[(1-Sp)(1-r_m)y_m^p + \big[Se(1-Sp)(1-r_m) - (1-Sp)(Se+Sp-1)(1-r_m)^{n_m^p}\big](1-y_m^p)\Big].
\end{aligned}
$$

<u>(ii) $\lambda_m \mathbb{E}_m^{\overline{p}}\big[FN\big] + (1-\lambda_m)\mathbb{E}_m^{\overline{p}}\big[FP\big]$</u>

$$
\begin{aligned}
&\lambda_m \mathbb{E}_m^{\overline{p}}\big[FN\big] + (1-\lambda_m)\mathbb{E}_m^{\overline{p}}\big[FP\big] \\
=&\Big(\lambda_m(1-z_m^{\overline{s}})H_m + (1-\lambda_m)z_m^{\overline{s}}(1-H_m)\Big)(1-l_m) \\
&+\lambda_m l_m\Big(\big[(1-Se)J_m y_m^r + (1-Se^2)J_m(1-y_m^r)\big]x_m^r + (1-z_m^s)J_m(1-x_m^r)\Big) \\
&+(1-\lambda_m)l_m\Big((1-Sp)(1-J_m)x_m^r y_m^r + z_m^s(1-J_m)(1-x_m^r) \\
&+\big[(1-Sp)^2(1-r_w)^{n_m^r-1} + Se(1-Sp)\big[1-(1-r_w)^{n_m^r-1}\big]\big](1-J_m)x_m^r(1-y_m^r)\Big).
\end{aligned}
$$

Then, the objective function in (94) can be written as

$$
\begin{aligned}
&\sum_{m=1}^{M}\Bigg[\Big(\lambda_m\mathbb{E}^{p}_{m}\big[FN\big]+(1-\lambda_m)\mathbb{E}^{p}_{m}\big[FP\big]\Big)\frac{p_m x^p_m}{P_T}+\Big(\lambda_m\mathbb{E}^{\overline{p}}_{m}\big[FN\big]+(1-\lambda_m)\mathbb{E}^{\overline{p}}_{m}\big[FP\big]\Big)\frac{p_m(1-x^p_m)}{P_T}\Bigg]\\
=&\sum_{m=1}^{M}\Bigg[\lambda_m\Big[(1-Se)r_m y^p_m+(1-Se^2)r_m(1-y^p_m)\Big]\\
&+(1-\lambda_m)\Big[(1-Sp)(1-r_m)y^p_m+\big[Se(1-Sp)(1-r_m)-(1-Sp)(Se+Sp-1)(1-r_m)^{n^p_m}\big](1-y^p_m)\Big]\Bigg]x^p_m\frac{p_m}{P_T}\\
&\Bigg[\Big(\lambda_m(1-z^{\overline{s}}_m)H_m+(1-\lambda_m)z^{\overline{s}}_m(1-H_m)\Big)(1-l_m)\\
&+\lambda_m l_m\Big(\big[(1-Se)J_m y^r_m+(1-Se^2)J_m(1-y^r_m)\big]x^r_m+(1-z^s_m)J_m(1-x^r_m)\Big)\\
&+(1-\lambda_m)l_m\Big((1-Sp)(1-J_m)x^r_m y^r_m+z^s_m(1-J_m)(1-x^r_m)\\
&+\big[(1-Sp)^2(1-r_w)^{n^r_m-1}+Se(1-Sp)\big[1-(1-r_w)^{n^r_m-1}\big]\big](1-J_m)x^r_m(1-y^r_m)\Big)\Bigg]\frac{p_m}{P_T}(1-x^p_m)\Bigg]\\
=&\sum_{m=1}^{M}\Big[A_m(y^p_m,n^p_m)x^p_m+D_m(y^r_m,n^r_m)x^r_m(1-x^p_m)+V_m(z^s_m)(1-x^r_m)(1-x^p_m)+C_m(z^{\overline{s}}_m)(1-x^p_m)\Big],
\end{aligned}
$$

where

$$
\begin{aligned}
A_m(y^p_m,n^p_m)=&\lambda_m\Big[(1-Se)r_m y^p_m+(1-Se^2)r_m(1-y^p_m)\Big]\frac{p_m}{P_T}\\
&+(1-\lambda_m)\Big[(1-Sp)(1-r_m)y^p_m+\big[Se(1-Sp)(1-r_m)-(1-Sp)(Se+Sp-1)(1-r_m)^{n^p_m}\big](1-y^p_m)\Big]\frac{p_m}{P_T},\\
D_m(y^r_m,n^r_m)=&\Big[\lambda_m l_m\Big(\big[(1-Se)J_m y^r_m+(1-Se^2)J_m(1-y^r_m)\big]\Big)+(1-\lambda_m)l_m\Big((1-Sp)(1-J_m)y^r_m\Big)\\
&+\big[(1-Sp)^2(1-r_w)^{n^r_m-1}+Se(1-Sp)\big[1-(1-r_w)^{n^r_m-1}\big]\big](1-J_m)(1-y^r_m)\Big)\Big]\frac{p_m}{P_T},\\
C_m(z^{\overline{s}}_m)=&\Big(\lambda_m(1-z^{\overline{s}}_m)H_m+(1-\lambda_m)z^{\overline{s}}_m(1-H_m)\Big)(1-l_m)\frac{p_m}{P_T},
\end{aligned}
$$

and

$$
V_m(z^s_m|\lambda_m)=\Big(\lambda_m J_m(1-z^s_m)+(1-\lambda_m)(1-J_m)z^s_m\Big)l_m\frac{p_m}{P_T}.
$$

B.3.2 Budget Constraint

According to Eq. (14), the budget constraint is given by:

$$
\mathbb{E}[TC]=\sum_{m=1}^{M}\Bigg[\mathbb{E}^{p}_{m}\big[TC\big]\frac{p_m x^p_m}{P_T}+\mathbb{E}^{\overline{p}}_{m}\big[TC\big]\frac{p_m(1-x^p_m)}{P_T}\Bigg].
$$

From Eq. (40) and (93), the expected terms are given by:

$$
\mathbb{E}^{p}_{m}\big[TC\big]=y^p_m+\left[\frac{1}{n^p_m}+Se-(Se+Sp-1)(1-r_m)^{n^p_m}\right](1-y^p_m),
$$

and

$$\mathbb{E}_m^{\overline{p}}[TC] = l_m y_m^r x_m^r + \Bigg[\big[(1-Se)\frac{1}{n_m^r} + Se(1+\frac{1}{n_m^r})\big](\alpha_m f_m - \alpha_m s_m f_m + s_m)\cdot r_m$$
$$+ f_m\alpha_m\big(1-(1-r_m)^{n_m^r-1}\big)\cdot(1-r_m) + \big[Sp\frac{1}{n_m^r} + (1-Sp)(1+\frac{1}{n_m^r})\big] f_m\alpha_m(1-r_m)^{n_m^r}\Bigg](1-y_m^r)x_m^r.$$

Substituting the last two equations into $\mathbb{E}[TC]$ gives:

$$\mathbb{E}[TC] = \sum_{m=1}^{M}\Bigg[\Bigg(y_m^p + \Big[\frac{1}{n_m^p} + Se - (Se+Sp-1)(1-r_m)^{n_m^p}\Big](1-y_m^p)\Bigg)x_m^p$$
$$+ \Bigg(l_m y_m^r + \Big[\big[(1-Se)\frac{1}{n_m^r} + Se(1+\frac{1}{n_m^r})\big](\alpha_m f_m - \alpha_m s_m f_m + s_m)\cdot r_m$$
$$+ f_m\alpha_m\big(1-(1-r_m)^{n_m^r-1}\big)\cdot(1-r_m) + \big[Sp\frac{1}{n_m^r} + (1-Sp)(1+\frac{1}{n_m^r})\big] f_m\alpha_m(1-r_m)^{n_m^r}\Big](1-y_m^r)\Bigg)(1-x_m^p)x_m^r\Bigg]\frac{p_m}{P_T}$$
$$= \sum_{m=1}^{M} A_m^c(y_m^p, n_m^p)x_m^p + D_m^c(y_m^r, n_m)(1-x_m^p)x_m^r.$$

where $A_m^c(y_m^p, n_m^p)$ and $D_m^c(y_m^r, n_m^r)$ are given by:

$$A_m^c(y_m^p, n_m^p) = \Bigg[y_m^p + \Bigg(\frac{1}{n_m^p} + Se - (Se+Sp-1)(1-r_m)^{n_m^p}\Bigg)(1-y_m^p)\Bigg]\frac{p_m}{P_T},$$

and

$$D_m^c(y_m^r, n_m^r) = \Bigg(l_m y_m^r + \Big[\big[(1-Se)\frac{1}{n_m^r} + Se(1+\frac{1}{n_m^r})\big](\alpha_m f_m - \alpha_m s_m f_m + s_m)\cdot r_m$$
$$+ f_m\alpha_m\big(1-(1-r_m)^{n_m^r-1}\big)\cdot(1-r_m) + \big[Sp\frac{1}{n_m^r} + (1-Sp)(1+\frac{1}{n_m^r})\big] f_m\alpha_m(1-r_m)^{n_m^r}\Big](1-y_m^r)\Bigg)\frac{p_m}{P_T}.$$

B.3.3 Optimization Model RP-MS

We begin by eliminating the decision variables y_m^p and y_m^p, which were introduced for the purpose of derivation, from the objective function. By treating individual testing as a special case of group testing (when $n^p, n^r = 1$). Consequently, we can express the objective function of **RP-MS** as:

$$\sum_{m=1}^{M}\Bigg[A_m(n^p)x_m^p + D_m(n^r)x_m^r(1-x_m^p) + V_m(z_m^s)(1-x_m^r)(1-x_m^p) + C_m(z_m^{\overline{s}})(1-x_m^p)\Bigg],$$

where A_m, D_m, V_m and C_m are summarized in Table 1 in S1 File. Similarly, the budget constraint can be written as:

$$\sum_{m=1}^{M}\Bigg[A_m^c(n^p)x_m^p + D_m^c(n^r)x_m^r(1-x_m^p)\Bigg] \le B,$$

where A_m^c and D_m^c can be found in Table 1 in Section B.3.3 in S1 File.

Table 1 in S1 File. Expression of performance metrics

$$
\begin{aligned}
A_m(1) &= \big[\lambda_m(1-Se)r_m + (1-\lambda_m)\big[(1-Sp)(1-r_m)\big]\big]p_m/P_T.\\
A_m(n), n\geq 2 &= \big[\lambda_m(1-Se^2)r_m + (1-\lambda_m)\big[Se(1-Sp)(1-r_m) - (1-Sp)(Se+Sp-1)(1-r_m)^n\big]\big]p_m/P_T.\\
A^c_m(1) &= p_m/P_T.\\
A^c_m(n), n\geq 2 &= \big[1/n^p_m + Se - (Se+Sp-1)(1-r_m)^n\big]p_m/P_T.\\
D_m(1) &= \big[\lambda_m l_m(1-Se)J_m + (1-\lambda_m)l_m(1-Sp)(1-J_m)\big]p_m/P_T.\\
D_m(n), n\geq 2 &= \big[\lambda_m l_m(1-Se^2)J_m + (1-\lambda_m)l_m\big[(1-Sp)^2(1-r_w)^{n-1} + Se(1-Sp)\big[1-(1-r_w)^{n-1}\big](1-\\
&\quad J_m)\big]\big]p_m/P_T.\\
D^c_m(1) &= p_m l_m/P_T.\\
D^c_m(n), n\geq 2 &= \big[\big[(1-Se)1/n + Se(1+1/n)\big](\alpha_m f_m - \alpha_m s_m f_m + s_m)\cdot r_m + f_m\alpha_m\big(1-(1-r_m)^{n-1}\big)\cdot(1-\\
&\quad r_m) + \big[Sp/n + (1-Sp)(1+1/n)\big]f_m\alpha_m(1-r_m)^n\big]p_m/P_T.\\
C_m(z^{\bar{s}}_m) &= \big[\lambda_m(1-z^{\bar{s}}_m)H_m + (1-\lambda_m)z^{\bar{s}}_m(1-H_m)\big](1-l_m)p_m/P_T.\\
V_m(z^s_m) &= \big[\lambda_m J_m(1-z^s_m) + (1-\lambda_m)(1-J_m)z^s_m\big]l_m p_m/P_T.
\end{aligned}
$$

B.3.4 Optimization Model RP-MS($\boldsymbol{n}, \boldsymbol{z}^*$)

In **RP-MS**($\boldsymbol{n}, \boldsymbol{z}^*$), for a given $\boldsymbol{z}^*$, the terms involving only $\boldsymbol{z}$ can be treated as constants. Therefore, we can rewrite the objective function of **RP-MS** as follows:

$$
\begin{aligned}
&\sum_{m=1}^{M}\Big[A_m(n^p)x^p_m + D_m(n^r)x^r_m(1-x^p_m) + V_m(z^s_m)(1-x^r_m)(1-x^p_m) + C_m(z^{\bar{s}}_m)(1-x^p_m)\Big]\\
&=\sum_{m=1}^{M}\Big[\Big(A_m(n^p) - V_m(z^s_m) - C_m(z^{\bar{s}}_m)\Big)x^p_m + \Big(D_m(n^r) - V_m(z^s_m)\Big)x^r_m(1-x^p_m) + V_m(z^s_m) + C_m(z^{\bar{s}}_m)\Big].
\end{aligned}
$$

For a given $\boldsymbol{z}^*$, the terms $V_m(z^s)$ and $C_m(z^{\bar{s}})$ involving only $\boldsymbol{z}$ can be treated as constants. Consequently, the constant terms can be omitted from the objective function. As such, the objective function can be written as:

$$
\sum_{m=1}^{M}\Big[\tilde{A}_m(n^p, \boldsymbol{z}^*_m)x^p_m + \tilde{D}_m(n^r, \boldsymbol{z}^*_m)x^r_m(1-x^p_m)\Big],
$$

where $\tilde{A}_m(n^p, \boldsymbol{z}^*_m) = A_m(n^p) - V_m(z^{s*}_m) - C_m(z^{\bar{s}*}_m)$ and $\tilde{D}_m(n^r, z^*_m) = D_m(n^r_m) - V_m(z^{s*}_m)$. The expressions of $\tilde{A}_m(n^p), \tilde{D}_m(n^p), V_m(z^{s*}_m)$ and $C_m(z^{\bar{s}*}_m)$ can be found in Table 1 in S1 File. On the other hand, the budget constraint of **RP-MS**($\boldsymbol{n}, \boldsymbol{z}^*$) remains same as **RP-MS**.

C Mathematical Proofs

Proof of Lemma 1. We first identify the optimal classification policy for untested non-symptomatic cases in category m (i.e, $z_m^{\bar{s}}$). The expression of $(\boldsymbol{I}-\boldsymbol{x}^p)^\top \boldsymbol{C}(\boldsymbol{z}^{\bar{s}})$ is given by:

$$\begin{aligned}(\boldsymbol{I}-\boldsymbol{x}^p)^\top \boldsymbol{C}(\boldsymbol{z}^{\bar{s}}) =& \sum_{m=1}^{M}\Bigg(\lambda_m(1-z_m^{\bar{s}})H_m + (1-\lambda_m)z_m^{\bar{s}}(1-H_m)\Bigg)(1-l_m)\frac{p_m(1-x_m^p)}{P_T} \\ =& \sum_{i=1}^{M}\Bigg[\lambda_m H_m + \Bigg(-\lambda_m H_m + (1-\lambda_m)(1-H_m)\Bigg)z_m^{\bar{s}}\Bigg](1-l_m)\frac{p_m(1-x_m^p)}{P_T}. \quad (95)\end{aligned}$$

Since Problem **RP-MS** is a minimization problem and the decision variables $z_m^{\bar{s}}$ only appear in the objective function, it is optimal to set $z_m^{\bar{s}}$ to one when the coefficient of $z_m^{\bar{s}}$ is smaller or equal to zero. Therefore, we have the following condition:

$$\Bigg(-\lambda_m H_m + (1-\lambda_m)(1-H_m)\Bigg)(1-l_m)\frac{p_m(1-x_m^p)}{P_T} \leq 0,$$

which leads to

$$\lambda_m \geq 1 - H_m.$$

The lase inequality is obtained by noting that $1-l_m, 1-x_m^p \geq 0$, and $p_m/P_T > 0$. As such, if, for a given λ_m, the condition $\lambda_m \geq 1-H_m$ holds, it is optimal to classify the untested non-symptomatic cases as positive in category m (i.e, $z^{\bar{s}^*} = 1$).

Similarly, for untested symptomatic cases, the corresponding performance metric is given by:

$$\begin{aligned}(\boldsymbol{I}-\boldsymbol{x}^p)^\top \boldsymbol{V}(\boldsymbol{z}^s)(\boldsymbol{I}-\boldsymbol{x}^r) =& \sum_{m=1}^{M}\lambda_m l_m\Bigg(\big[(1-Se)J_m y_m^r + (1-Se^2)J_m(1-y_m^r)\big]x_m^r + (1-z^s)J_m(1-x_m^r)\Bigg)\frac{p_m(1-x_m^p)}{P_T} \\ &+ (1-\lambda_m)l_m\Bigg((1-Sp)(1-J_m)x_m^r y_m^r + z^s(1-J_m)(1-x_m^r) \\ &+ \big[(1-Sp)^2(1-r_w)^{n_m-1} + Se(1-Sp)\big[1-(1-r_w)^{n_m-1}\big]\big](1-J_m)x_m^r(1-y_m^r)\Bigg)\frac{p_m(1-x_m^p)}{P_T}.\end{aligned}$$

The terms related including z^s are given by:

$$\begin{aligned}&\Bigg[\lambda_m l_m J_m(1-x_m^r)(1-z^s) + (1-\lambda_m)l_m(1-J_m)(1-x_m^r)z^s\Bigg]\frac{p_m(1-x_m^p)}{P_T} \\ =& l_m(1-x_m^r)\Bigg[1-\lambda_m - J_m\Bigg]\frac{p_m(1-x_m^p)}{P_T}z_m^s.\end{aligned}$$

If the coefficient of z_m^s is smaller or equal to zero, it is optimal to classify the untested non-symptomatic subjects as positive (i.e., $z_m^{s^*} = 1$), which leads to the following condition:

$$l_m(1-x_m^r)\frac{p_m(1-x_m^p)}{P_T}\Bigg[1-\lambda_m-J_m\Bigg] \leq 0.$$

As such, we have

$$\lambda_m \geq 1 - J_m.$$

The last equation is obtained by noting that $l_m, 1-x_m^r, 1-x_m^p \geq 0$ and $p_m/P_T > 0$. Therefore, if,

for a given λ_m, the condition $\lambda_m \geq 1 - J_m$ holds, it is optimal to classify the untested symptomatic cases as positive in category m (i.e, $z^{s^*} = 1$).

Proof of Lemma 2 & Corollary 1. We prove the results by considering a relaxation of Problem $\mathbf{RP\text{-}MS}(\boldsymbol{n}, \boldsymbol{z}^*)$ where the budget constraint is omitted. Then, the resulting problem becomes an unconstrained problem except the bounding domain for decision variables (i.e., x^r_m, $x^p_m \in [0,1]$). Since the objective function is a sum of objective value from each category $m \in M$ and each category is independent from others, then finding the minimum value of the objective function is equivalent to minimize each category. To facilitate the presentation, the objective value of category m can be written as ($(n, \boldsymbol{z}*)$ is dropped from the expression):

$$\min_{x^p_m, x^r_m} f_m(x^p_m, x^r_m) = \tilde{A}_m x^p_m + \tilde{D}_m x^r_m (1 - x^p_m).$$

We first demonstrate that the decision variable x^r_m is guaranteed to be zero in the optimal solution only when $\tilde{D}_m \geq 0$. We first take the derivation of $f_m(x^p_m, x^r_m)$ with respect to x^r_m, which gives:

$$\frac{\partial f_m}{\partial x^r_m} = \tilde{D}_m (1 - x^p_m).$$

If $\tilde{D}_m \geq 0$, then $\partial f / \partial x^r_m \geq 0$ (given that $x^p_m \in [0,1]$). Therefore, it is optimal to set x^r_m to zero since f_m is increasing with respect to x^r_m. On the other hand, if $\tilde{D}_m < 0$, then $\partial f / \partial x^r_m < 0$ (given that $x^p_m \in [0,1]$). In this case, it is optimal to set x^r_m to one since f_m is increasing with respect to x^r_m. As such, in what follows, we consider two scenarios based on the value of $\tilde{D}_m$:

<u>(i) $\tilde{D}_m \geq 0$</u>: We can take the derivation of the objective function with respect to x^p_m, leading to:

$$\frac{\partial f_m}{\partial x^p_m} = \tilde{A}_m - \tilde{D}_m x^r_m.$$

We consider two sub-scenarios: (ia) $\tilde{A}_m < \tilde{D}_m$ and (ib) $\tilde{A}_m \geq \tilde{D}_m$:

<u>(ia) $\tilde{A}_m < \tilde{D}_m$ </u>: Since $\tilde{D}_m \geq 0$, we have shown that the optimal solution $x^{r*}_m = 0$, which leads to $\partial f / \partial x^p_m = \tilde{A}_m$. If $\tilde{A}_m \geq 0$, then $\partial f / \partial x^p_m \geq 0$, indicating that f_m is increasing in $x^p_m \in [0,1]$. Hence, it is optimal to set $x^{p*}_m = 0$; On the other hand, if $\tilde{A}_m < 0$, then $\partial f / \partial x^p_m < 0$, indicating that f_m is decreasing in $x^p_m \in [0,1]$. Hence, it is optimal to set $x^{p*}_m = 1$.

<u>(ib) $\tilde{A}_m \geq \tilde{D}_m$ </u>: The partial derivative can be written as:

$$\tilde{A}_m - \tilde{D}_m x^r_m \geq \tilde{D}_m - \tilde{D}_m x^r_m \geq \tilde{D}_m (1 - x^r_m) \geq 0,$$

indicating that f_m is increasing in $x^p_m \in [0,1]$. Hence, it is optimal to set $x^{p*}_m = 0$.

<u>(ii) $\tilde{D}_m < 0$</u>: Similarly, we consider two sub-scenarios: (iia) $\tilde{A}_m < \tilde{D}_m$ and (iib) $\tilde{A}_m \geq \tilde{D}_m$:

<u>(iia) $\tilde{A}_m < \tilde{D}_m$ </u>: The partial derivative of f_m with respect to x^p_m can be written as:

$$\tilde{A}_m - \tilde{D}_m x^r_m < \tilde{D}_m - \tilde{D}_m x^r_m = \tilde{D}_m (1 - x^r_m) < 0.$$

The last equation indicates f_m is decreasing in $x^p_m \in [0,1]$. Hence, it is optimal to set $x^{p*}_m = 1$.

(iib) $\tilde{A}_m \geq \tilde{D}_m$: Since $D_m < 0$, we have proven that $x^{r*} = 1$ under this scenario, which leads the partial derivative of f_m with respect to x^p_m to $\tilde{A}_m - \tilde{D}_m \geq 0$. As such, f_m is increasing in x^p_m, indicating that it is optimal to set $x^{p*}_m = 0$.

In summary, when there is no budget constrain, if both $\tilde{A}_m$ and $\tilde{D}_m$ are greater than or equal to 0 ($\min\{\tilde{A}_m, \tilde{D}_m\} \geq 0$), it is optimal to set both x^{p*}_m and x^{r*}_m to 0, resulting in a zero budget cost. On the other hand, if at least one of the parameters $\tilde{A}_m$ or $\tilde{D}_m$ is negative ($\min\{\tilde{A}_m, \tilde{D}_m\} < 0$), the optimal decision variable depends on the values of $\tilde{A}_m$ and $\tilde{D}_m$. If $\tilde{A}_m < \tilde{D}_m$ ($\tilde{A}_m \geq \tilde{D}_m$), it is optimal to set $x^{p*}_m = 1$ ($x^{p*}_m = 0$) and $x^{r*}_m = 0$ ($x^{p*}_m = 1$), resulting in a total budget cost of $\tilde{A}^c_m$ ($\tilde{D}^c_m$). □

Proof of Lemma 4. (i) $\frac{\tilde{A}_m(n,\boldsymbol{z}^*|\lambda_m)}{A^c_m(n|\lambda_m)} \leq \frac{\tilde{D}_m(n,\boldsymbol{z}^*|\lambda_m)}{D^c_m(n|\lambda_m)}$ and $\tilde{A}_m(n,\boldsymbol{z}^*|\lambda_m) \leq \tilde{D}_m(n,\boldsymbol{z}^*|\lambda_m)$:

We begin by noting that if $\tilde{A}_m(n,\boldsymbol{z}^*|\lambda_m) \leq \tilde{D}_m(n,\boldsymbol{z}^*|\lambda_m) < 0$, the optimal performance that can be achieved is $\tilde{A}_m(n,\boldsymbol{z}^*|\lambda_m)$ when $x^{p*}_m = 1$ and $x^{r*}_m = 0$, as proven in Lemma 2. This optimal performance requires a budget allocation of $\tilde{A}^c_m$ for category m. As such, when B_m (the budget allocation for category m) is greater than $\tilde{A}^c_m$, we have $x^{p*}_m = 1$ and $x^{r*}_m = 0$. In what follows, we then prove the case when $B_m < \tilde{A}^c_m$. To prove the result, suppose, by contradiction, the optimal solution $x^{r*}_m > 0$ when $\frac{\tilde{A}_m(n,\boldsymbol{z}^*|\lambda_m)}{A^c_m(n|\lambda_m)} \leq \frac{\tilde{D}_m(n,\boldsymbol{z}^*|\lambda_m)}{D^c_m(n|\lambda_m)}$ and $\tilde{A}_m(n,\boldsymbol{z}^*|\lambda_m) \leq \tilde{D}_m(n,\boldsymbol{z}^*|\lambda_m)$. We show that the objective function can be improved by decreasing x^{r*}_m and increasing x^{p*}_m. The budget cost by x^{r*}_m is given by (to facilitate the presentation, the terms $(n,\boldsymbol{z}^*|\lambda_m)$ and $(n,|\lambda_m)$ are dropped from the expression): $\tilde{D}^c_m(1-x^{p*}_m)x^{r*}_m$. Using the same budget without violating the budget constraint, we construct a new solution:

$$\tilde{x}^{p*}_m = x^{p*}_m + \frac{\tilde{D}^c_m(1-x^{p*}_m)x^{r*}_m}{\tilde{A}^c_m}; \tilde{x}^{r*}_m = 0.$$

Since $\tilde{A}^c_m x^{p*}_m + \tilde{D}^c_m(1-x^{p*}_m) < \tilde{A}^c_m$, $\tilde{x}^{p*}_m < 1$. The new objective value is given by:

$$\tilde{f} = \tilde{A}_m\big(x^{p*}_m + \frac{\tilde{D}^c_m(1-x^{p*}_m)x^{r*}_m}{\tilde{A}^c_m}\big) = \tilde{A}_m x^{p*}_m + \frac{\tilde{A}_m\tilde{D}^c_m}{\tilde{A}^c_m}(1-x^{p*}_m)\tilde{x}^{r*}_m.$$

Comparing it with the original objective value gives:

$$\begin{aligned}\tilde{f} - f =& \big(\tilde{A}_m x^{p*}_m + \frac{\tilde{A}_m\tilde{D}^c_m}{\tilde{A}^c_m}(1-x^{p*}_m)\tilde{x}^{r*}_m\big) - \big(\tilde{A}_m x^{p*}_m + \tilde{D}_m(1-x^{p*}_m)x^{r*}_m\big)\\ =& \big(\frac{\tilde{A}_m\tilde{D}^c_m}{\tilde{A}^c_m} - \tilde{D}_m\big)(1-x^{p*}_m)x^{r*}_m = \frac{\tilde{A}_m\tilde{D}^c_m - \tilde{D}_m\tilde{A}^c_m}{\tilde{A}^c_m}(1-x^{p*}_m)x^{r*}_m\end{aligned}$$

Given that $\tilde{A}_m/\tilde{A}^c_m \leq \tilde{D}_m/\tilde{D}^c_m$, we can conclude that $\tilde{A}_m\tilde{D}^c_m \leq \tilde{D}_m\tilde{A}^c_m$, which implies $\tilde{f} - f < 0$. This contracts the fact that x^{r*}_m is optimal, completing the proof.

(ii) $\frac{\tilde{A}_m(n,\boldsymbol{z}^*|\lambda_m)}{A^c_m(n|\lambda_m)} > \frac{\tilde{D}_m(n,\boldsymbol{z}^*|\lambda_m)}{D^c_m(n|\lambda_m)}$ and $\tilde{A}_m(n,\boldsymbol{z}^*|\lambda_m) > \tilde{D}_m(n,\boldsymbol{z}^*|\lambda_m)$:

Similarly, we prove the result by contradiction when $B_m < \tilde{D}^c_m$ (when $\tilde{A}_m(n,\boldsymbol{z}^*|\lambda_m) > \tilde{D}_m(n,\boldsymbol{z}^*|\lambda_m$ and $B_m > \tilde{D}^c_m$, $x^{p*}_m = 0$, as proven in Lemma 2. Assume the optimal solution $x^{p*}_m > 0$ when $\frac{\tilde{A}_m(n,\boldsymbol{z}^*|\lambda_m)}{A^c_m(n|\lambda_m)} > \frac{\tilde{D}_m(n,\boldsymbol{z}^*|\lambda_m)}{D^c_m(n|\lambda_m)}$ and $\tilde{A}_m(n,\boldsymbol{z}^*|\lambda_m) > \tilde{D}_m(n,\boldsymbol{z}^*|\lambda_m)$. We show that the objective func-

tion can be improved by decreasing x_m^{p*} and increasing x_m^{r*}. The budget cost by x_m^{p*} is given by: $\tilde{A}_m^c x_m^{p*}$. Using the same budget without violating the budget constraint, we construct a new solution:

$$\tilde{x}_m^{p*} = 0; \tilde{x}_m^{r*} = x_m^{r*} + \frac{\tilde{A}_m^c x_m^{p*}}{\tilde{D}_m^c}.$$

The new objective value is given by:

$$\tilde{f} = \tilde{D}_m \big(x_m^{r*} + \frac{\tilde{A}_m^c x_m^{p*}}{\tilde{D}_m^c} \big) = \tilde{D}_m x_m^{r*} + \frac{\tilde{D}_m \tilde{A}_m^c}{\tilde{D}_m^c} \tilde{x}_m^{p*}.$$

Comparing it with the original objective value gives:

$$\begin{aligned}\tilde{f} - f =& \big(\tilde{D}_m x_m^{r*} + \frac{\tilde{D}_m \tilde{A}_m^c}{\tilde{D}_m^c} \tilde{x}_m^{p*} \big) - \big(\tilde{A}_m x_m^{p*} + \tilde{D}_m (1 - x_m^{p*}) x_m^{r*} \big) \\ =& \left(\tilde{D}_m x_m^{r*} + \frac{\tilde{D}_m \tilde{A}_m^c - \tilde{A}_m \tilde{D}_m^c}{\tilde{D}_m^c} \right) x_m^{p*}.\end{aligned}$$

Given that $\tilde{D}_m < 0$, $\tilde{A}_m / \tilde{A}_m^c > \tilde{D}_m / \tilde{D}_m^c$, we can conclude that $\tilde{A}_m \tilde{D}_m^c > \tilde{D}_m \tilde{A}_m^c$, which implies $\tilde{f} - f < 0$. This contracts the fact that x_m^{p*} is optimal, completing the proof. □

Proof of Lemma 5. We prove the results by contradiction. If there exists, two categories m and v have two distinct values at optimality (i.e., $x_m^{p*}, x_m^{r*} > 0$ and $x_v^{p*}, x_v^{r*} > 0$), we can always construct a new solution with at most two distinct values in one category that improves the objective value (scenarios having more than two categories will simply follow the proof). According to Lemma 4, if $m, v \notin \mathcal{F}_1(\boldsymbol{n}^*, \boldsymbol{z}, \boldsymbol{\lambda}) \cup \mathcal{F}_2(\boldsymbol{n}^*, \boldsymbol{z}, \boldsymbol{\lambda})$ (to facilitate the presentation, the term $(\boldsymbol{n}^*, \boldsymbol{z}, \boldsymbol{\lambda})$ will be dropped), the optimal solution follows that $x_m^{p*} = 0 / x_m^{r*} = 0$ and $x_v^{p*} = 0 / x_v^{r*} = 0$. This implies that only one variable can be chosen from each category. However, since we assume that both m and v take two nonzero values, m, v must be in the set $\mathcal{F}_1 \cup \mathcal{F}_2$. We then consider three cases: (i) $m \in \mathcal{F}_1$ and $v \in \mathcal{F}_1$ (ii) $m \in \mathcal{F}_1$ and $v \in \mathcal{F}_2$ (the case when $m \in \mathcal{F}_2$ and $v \in \mathcal{F}_1$ is equivalent to case(ii), so no need to consider it as an additional scenario) and (iii) $m \in \mathcal{F}_2$ and $v \in \mathcal{F}_2$.

(i) $m \in \mathcal{F}_1$ and $v \in \mathcal{F}_1$: Since $\frac{\tilde{A}_m}{A_m^c} \leq \frac{\tilde{D}_m}{D_m^c}$, $\tilde{A}_m > \tilde{D}_m$ for category m and $\frac{\tilde{A}_v}{A_v^c} \leq \frac{\tilde{D}_v}{D_v^c}$, $\tilde{A}_v > \tilde{D}_v$ for category v. Under this scenario, according to Corollary 2, we know that x_m^{r*} and x_v^{r*} must be binary. Since we assuming $x_m^{r*}, x_v^{r*} > 0$, the optimal solution must be $x_m^{p*} > 0$ with $x_m^{r*} = 1$ and $x_v^{p*} > 0$ with $x_v^{r*} = 1$. Without loss of generality, we assume

$$\frac{\tilde{D}_m - \tilde{A}_m}{D_m^c - A_m^c} \leq \frac{\tilde{D}_v - \tilde{A}_v}{D_v^c - A_v^c}.$$

If the assumption does not hold, we can simply construct the following new solution in an opposite way. Next, we construct a new solution:

$$\tilde{x}_m^{p*} = x_m^{p*} - \delta, \tilde{x}_m^{r*} = 1; \tilde{x}_v^{p*} = 1, \tilde{x}_v^{r*} = 0.$$

Note that the new solution now includes only one category having two non-zero values. To maintain the feasibility of budget constraint, we first determine the value of δ. The budget difference of

category v between the original and the new solution is given by:

$$\tilde{B}_v - B_v = A_v^c - \left[A_v^c x_v^{p*} + D_v^c(1 - x_v^{p*})\right]$$
$$= (A_v^c - D_v^c)(1 - x_v^{p*}).$$

Under scenario (i), we know $A_v^c \leq D_v^c$. As such, the new solution saves budget $(A_v^c - D_v^c)(1 - x_v^{p*})$ compared to the original optimal solution. For category m, the budget difference is given by:

$$\tilde{B}_m - B_m = \left[A_m^c(x_m^{p*} - \delta) + D_m^c(1 - x_m^{p*} + \delta)\right] - \left[A_m^c x_m^{p*} + D_m^c(1 - x_m^{p*})\right]$$
$$= (D_m^c - A_m^c)\delta > 0.$$

As such, category m costs more budget in the new optimal solution. Therefore, we use the additional budget from category v for category m and set δ as:

$$\delta = \frac{\tilde{B}_v - B_v}{(A_m^c - D_m^c)} = \frac{(A_v^c - D_v^c)(1 - x_v^{p*})}{(A_m^c - D_m^c)}.$$

To maintain the feasibility of non-negativity constraint of $\tilde{x}_m^{p*} \in [0, 1]$, we consider two sub-scenarios: (ia) $\delta \leq x_m^{p*}$ and (ib) $\delta > x_m^{p*}$.

(ia) $\delta \leq x_m^{p*}$: Since $\delta \leq x_m^{p*}$, the new solution maintains the feasibility of the budget constraint and non-negativity constraints. The difference of the new objective values between two solutions can be written as:

$$\tilde{f} - f = \left[\tilde{A}_m(x_m^{p*} - \delta) + \tilde{D}_m(1 - x_m^{p*} + \delta) + \tilde{A}_v\right] - \left[\tilde{A}_m x_m^{p*} + \tilde{D}_m(1 - x_m^{p*}) + \tilde{A}_v x_v^{p*} + \tilde{D}_v(1 - x_v^{p*})\right]$$
$$= (\tilde{D}_m - \tilde{A}_m)\delta + (\tilde{A}_v - \tilde{D}_v)(1 - x_v^{p*}).$$

Substituting δ into the last equation gives:

$$\tilde{f} - f = (\tilde{D}_m - \tilde{A}_m)\frac{(A_v^c - D_v^c)(1 - x_v^{p*})}{(A_m^c - D_m^c)} + (\tilde{A}_v - \tilde{D}_v)(1 - x_v^{p*})$$
$$= \left[(\tilde{D}_m - \tilde{A}_m)\frac{A_v^c - D_v^c}{A_m^c - D_m^c} + \tilde{A}_v - \tilde{D}_v\right](1 - x_v^{p*}).$$

Since $D_v^c \geq A_v^c$, we have

$$\frac{\tilde{D}_m - \tilde{A}_m}{D_m^c - A_m^c} \leq \frac{\tilde{D}_v - \tilde{A}_v}{D_v^c - A_v^c} \Rightarrow (\tilde{D}_m - \tilde{A}_m)\frac{A_v^c - D_v^c}{A_m^c - D_m^c} + \tilde{A}_v - \tilde{D}_v \leq 0.$$

Hence, the inequality $\tilde{f} - f \leq 0$ suggests that the new solution attains a superior objective value. This contradicts the fact that the initial solution is optimal.

(ib) $\delta > x_m^{p*}$: We modify the new solution as:

$$\tilde{x}_m^{p*} = 0, \tilde{x}_m^{r*} = 1; \tilde{x}_v^{p*} = \Delta, \tilde{x}_v^{r*} = 1.$$

The new solution only has one category that has two non-zero values. The budget different is given

by:

$$\tilde{B} - B = (D^c_m - A^c_m)x^{p*}_m + (D^c_v - A^c_v)x^{p*}_v + (A^c_v - D^c_v)\Delta.$$

To maintain the feasibility of the budget constraint, we set the value of the last equation to zero in order to determine the value of Δ:

$$\Delta = \frac{(D^c_m - A^c_m)x^{p*}_m + (D^c_v - A^c_v)x^{p*}_v}{D^c_v - A^c_v}.$$

Next, we show the selected Δ remains the feasibility of $\tilde{x}^{p*}_v \leq 1$. Since $\delta > x^{p*}_m$, we have

$$\frac{(A^c_v - D^c_v)(1 - x^{p*}_v)}{(A^c_m - D^c_m)} > x^{p*}_m,$$

which leads to:

$$(D^c_m - A^c_m)x^{p*}_m + (D^c_v - A^c_v)x^{p*}_v < D^c_v - A^c_v.$$

Therefore, $\tilde{x}^{p*}_v = \Delta < 1$. Next, the difference of the new objective values between two solutions can be written as:

$$\begin{aligned}
\tilde{f} - f =& (\tilde{D}_m - \tilde{A}_m)x^{p*}_m + (\tilde{D}_v - \tilde{A}_v)x^{p*}_v + (\tilde{A}_v - \tilde{D}_v)\Delta \\
=& (\tilde{D}_m - \tilde{A}_m)x^{p*}_m + (\tilde{D}_v - \tilde{A}_v)x^{p*}_v + (\tilde{A}_v - \tilde{D}_v)\frac{(D^c_m - A^c_m)x^{p*}_m + (D^c_v - A^c_v)x^{p*}_v}{D^c_v - A^c_v} \\
=& \left[(\tilde{D}_m - \tilde{A}_m) - \frac{D^c_m - A^c_m}{D^c_v - A^c_v}(D^c_m - A^c_m)\right]x^{p*}_m.
\end{aligned}$$

Since $\frac{\tilde{D}_m - \tilde{A}_m}{D^c_m - A^c_m} \leq \frac{\tilde{D}_v - \tilde{A}_v}{D^c_v - A^c_v}$, we have

$$(\tilde{D}_m - \tilde{A}_m) - \frac{D^c_m - A^c_m}{D^c_v - A^c_v}(D^c_m - A^c_m) \leq 0.$$

Therefore, the inequality $\tilde{f} - f \leq 0$ suggests that the new solution attains a better objective value, contradicting the fact that the initial solution is optimal.

<u>(ii) $m \in \mathcal{F}_1$ and $v \in \mathcal{F}_2$</u>: Since $\frac{\tilde{A}_m}{A^c_m} \leq \frac{\tilde{D}_m}{D^c_m}$, $\tilde{A}_m > \tilde{D}_m$ for category m and $\frac{\tilde{A}_v}{A^c_v} \geq \frac{\tilde{D}_v}{D^c_v}$, $\tilde{A}_v < \tilde{D}_v$ for category v. Under this scenario, according to Corollary 2, we know that x^{r*}_m must be binary and therefore $x^{r*}_m = 1$. For category v, $x^{r*}_v = 1$ if $x^{p*}_v > 0$. Without loss of generality, we assume

$$\frac{\tilde{D}_m - \tilde{A}_m}{D^c_m - A^c_m} \leq \frac{\tilde{A}_v - \tilde{D}_v}{A^c_v - D^c_v}.$$

Next, we construct a new solution:

$$\tilde{x}^{p*}_m = x^{p*}_m - \delta, \tilde{x}^{r*}_m = 1; \tilde{x}^{p*}_v = 0, \tilde{x}^{r*}_v = 1.$$

The new solution now includes only one category having two non-zero values. To maintain the feasibility of budget constraint, we first determine the value of δ. The budget difference of category

v between the original and the new solution is given by:

$$\begin{aligned}\tilde{B}_v - B_v =& D_v^c - \left[A_v^c x_v^{p*} + D_v^c(1 - x_v^{p*})\right] \\ =& (D_v^c - A_v^c) x_v^{p*}.\end{aligned}$$

Under scenario (i), we know $A_v^c \geq D_v^c$. As such, the new solution saves budget $(D_v^c - A_v^c)x_v^{p*}$. For category m, the budget difference is given by:

$$\begin{aligned}\tilde{B}_m - B_m =& \left[A_m^c(x_m^{p*} - \delta) + D_m^c(1 - x_m^{p*} + \delta)\right] - \left[A_m^c x_m^{p*} + D_m^c(1 - x_m^{p*})\right] \\ =& (D_m^c - A_m^c)\delta > 0.\end{aligned}$$

As such, category m costs more budget in the new optimal solution. Therefore, we use the additional budget from category v for category m and set δ as:

$$\delta = \frac{\tilde{B}_v - B_v}{(A_m^c - D_m^c)} = \frac{(D_v^c - A_v^c)x_v^{p*}}{(A_m^c - D_m^c)}.$$

To maintain the feasibility of non-negativity constraint of $\tilde{x}_m^{p*} \in [0, 1]$, we consider two sub-scenarios: (iia) $\delta \leq x_m^{p*}$ and (iib) $\delta > x_m^{p*}$.

(iia) $\delta \leq x_m^{p*}$: Since $\delta \leq x_m^{p*}$, the new solution maintains the feasibility of the budget constraint and non-negativity constraints. The difference of the new objective values between two solutions can be written as:

$$\begin{aligned}\tilde{f} - f =& \left[\tilde{A}_m(x_m^{p*} - \delta) + \tilde{D}_m(1 - x_m^{p*} + \delta) + \tilde{D}_v\right] - \left[\tilde{A}_m x_m^{p*} + \tilde{D}_m(1 - x_m^{p*}) + \tilde{A}_v x_v^{p*} + \tilde{D}_v(1 - x_v^{p*})\right] \\ =& (\tilde{D}_m - \tilde{A}_m)\delta + (\tilde{D}_v - \tilde{A}_v)x_v^{p*}.\end{aligned}$$

Substituting δ into the last equation gives:

$$\begin{aligned}\tilde{f} - f =& (\tilde{D}_m - \tilde{A}_m)\frac{(D_v^c - A_v^c)x_v^{p*}}{(A_m^c - D_m^c)} + (\tilde{D}_v - \tilde{A}_v)x_v^{p*} \\ =& \left[(\tilde{D}_m - \tilde{A}_m)\frac{D_v^c - A_v^c}{A_m^c - D_m^c} + \tilde{D}_v - \tilde{A}_v\right]x_v^{p*}.\end{aligned}$$

Since $A_v^c \geq D_v^c$, we have

$$\frac{\tilde{D}_m - \tilde{A}_m}{D_m^c - A_m^c} \leq \frac{\tilde{A}_v - \tilde{D}_v}{A_v^c - D_v^c} \Rightarrow (\tilde{D}_m - \tilde{A}_m)\frac{D_v^c - A_v^c}{A_m^c - D_m^c} + \tilde{D}_v - \tilde{A}_v \leq 0.$$

Hence, the inequality $\tilde{f} - f \leq 0$ suggests that the new solution attains a superior objective value. This contradicts the fact that the initial solution is optimal.

(iib) $\delta > x_m^{p*}$: We modify the new solution as:

$$\tilde{x}_m^{p*} = 0, \tilde{x}_m^{r*} = 1; \tilde{x}_v^{p*} = x_v^{p*} - \Delta, \tilde{x}_v^{r*} = 1.$$

The new solution only has one category that has two non-zero values. The budget different is given

by:

$$\tilde{B} - B = (D^c_m - A^c_m)x^{p*}_m + (D^c_v - A^c_v)\Delta.$$

To maintain the feasibility of the budget constraint, we set the value of the last equation to zero in order to determine the value of Δ:

$$\Delta = \frac{(D^c_m - A^c_m)x^{p*}_m}{A^c_v - D^c_v}.$$

Next, we show the selected Δ remains the feasibility of $\tilde{x}^{p*}_v \le 1$. Since $\delta > x^{p*}_m$, we have

$$\frac{(D^c_v - A^c_v)x^{p*}_v}{(A^c_m - D^c_m)} > x^{p*}_m,$$

which leads to:

$$(A^c_v - D^c_v)x^{p*}_v > (D^c_m - A^c_m)x^{p*}_m.$$

Therefore, $\Delta < x^{p*}_m$, which leads to $\tilde{x}^{p*}_v < 1$. Next, the difference of the new objective values between two solutions can be written as:

$$\begin{aligned}\tilde{f} - f =&(\tilde{D}_m - \tilde{A}_m)x^{p*}_m + (\tilde{D}_v - \tilde{A}_v)\Delta\\ =&(\tilde{D}_m - \tilde{A}_m)x^{p*}_m + (\tilde{D}_v - \tilde{A}_v)\frac{(D^c_m - A^c_m)x^{p*}_m}{A^c_v - D^c_v}\\ =&\left[(\tilde{D}_m - \tilde{A}_m) - (\tilde{A}_v - \tilde{D}_v)\frac{(D^c_m - A^c_m)}{A^c_v - D^c_v}\right]x^{p*}_m.\end{aligned}$$

Since $\frac{\tilde{D}_m - \tilde{A}_m}{D^c_m - A^c_m} \le \frac{\tilde{A}_v - \tilde{D}_v}{A^c_v - D^c_v}$, we have

$$(\tilde{D}_m - \tilde{A}_m) - (\tilde{A}_v - \tilde{D}_v)\frac{D^c_m - A^c_m}{A^c_v - D^c_v} \le 0.$$

Therefore, the inequality $\tilde{f} - f \le 0$ suggests that the new solution attains a better objective value, contradicting the fact that the initial solution is optimal.

(iii) $m \in \mathcal{F}_2$ and $v \in \mathcal{F}_2$: Since $\frac{\tilde{A}_m}{A^c_m} \ge \frac{\tilde{D}_m}{D^c_m}$, $\tilde{A}_m < \tilde{D}_m$ for category m and $\frac{\tilde{A}_v}{A^c_v} \ge \frac{\tilde{D}_v}{D^c_v}$, $\tilde{A}_v < \tilde{D}_v$ for category v. Under this scenario, according to Corollary 2, we know that x^{r*}_m, x^{r*}_v must be one if $x^{p*}_m, x^{p*}_v > 0$. Without loss of generality, we assume

$$\frac{\tilde{A}_m - \tilde{D}_m}{A^c_m - D^c_m} \le \frac{\tilde{A}_v - \tilde{D}_v}{A^c_v - D^c_v}.$$

Next, we construct a new solution:

$$\tilde{x}^{p*}_m = x^{p*}_m + \delta, \tilde{x}^{r*}_m = 1; \tilde{x}^{p*}_v = 0, \tilde{x}^{r*}_v = 1.$$

The new solution now includes only one category having two non-zero values. To maintain the feasibility of budget constraint, we first determine the value of δ. The budget difference of category

v between the original and the new solution is given by:

$$\begin{aligned}\tilde{B}_v - B_v =& D_v^c - \Big[A_v^c x_v^{p*} + D_v^c(1 - x_v^{p*})\Big] \\ =& (D_v^c - A_v^c) x_v^{p*}.\end{aligned}$$

Under scenario (i), we know $A_v^c \geq D_v^c$. As such, the new solution saves budget $(D_v^c - A_v^c)x_v^{p*}$. For category m, the budget difference is given by:

$$\begin{aligned}\tilde{B}_m - B_m =& \Big[A_m^c(x_m^{p*} + \delta) + D_m^c(1 - x_m^{p*} - \delta)\Big] - \Big[A_m^c x_m^{p*} + D_m^c(1 - x_m^{p*})\Big] \\ =& (A_m^c - D_m^c)\delta > 0.\end{aligned}$$

As such, category m costs more budget in the new optimal solution. Therefore, we use the additional budget from category v for category m and set δ to

$$\delta = \frac{\tilde{B}_v - B_v}{(D_m^c - A_m^c)} = \frac{(D_v^c - A_v^c)x_v^{p*}}{(D_m^c - A_m^c)}.$$

To maintain the feasibility of non-negativity constraint of $\tilde{x}_m^{p*} \in [0, 1]$, we consider two sub-scenarios (iiia) $\delta \leq 1 - x_m^{p*}$ and (iiib) $\delta > 1 - x_m^{p*}$.

(iiia) $\delta \leq x_m^{p*}$: Since $\delta \leq 1 - x_m^{p*}$, the new solution maintains the feasibility of the budget constraint and non-negativity constraints. The difference of the new objective values between two solutions can be written as:

$$\begin{aligned}\tilde{f} - f =& \Big[\tilde{A}_m(x_m^{p*} + \delta) + \tilde{D}_m(1 - x_m^{p*} - \delta) + \tilde{D}_v\Big] - \Big[\tilde{A}_m x_m^{p*} + \tilde{D}_m(1 - x_m^{p*}) + A_v x_v^{p*} + \tilde{D}_v(1 - x_v^{p*})\Big] \\ =& (\tilde{A}_m - \tilde{D}_m)\delta + (\tilde{D}_v - \tilde{A}_v)x_v^{p*}.\end{aligned}$$

Substituting δ into the last equation gives:

$$\begin{aligned}\tilde{f} - f =& (\tilde{A}_m - \tilde{D}_m)\frac{(D_v^c - A_v^c)x_v^{p*}}{(D_m^c - A_m^c)} + (\tilde{D}_v - \tilde{A}_v)x_v^{p*} \\ =& \Big[(\tilde{A}_m - \tilde{D}_m)\frac{D_v^c - A_v^c}{D_m^c - A_m^c} + \tilde{D}_v - \tilde{A}_v\Big]x_v^{p*}.\end{aligned}$$

Since $A_v^c \geq D_v^c$, we have

$$\frac{\tilde{A}_m - \tilde{D}_m}{A_m^c - D_m^c} \leq \frac{\tilde{A}_v - \tilde{D}_v}{A_v^c - D_v^c} \Rightarrow (\tilde{A}_m - \tilde{D}_m)\frac{D_v^c - A_v^c}{D_m^c - A_m^c} + \tilde{D}_v - \tilde{A}_v \leq 0.$$

Hence, the inequality $\tilde{f} - f \leq 0$ suggests that the new solution attains a superior objective value. This contradicts the fact that the initial solution is optimal.

(iiib) $\delta > 1 - x_m^{p*}$: We modify the new solution as:

$$\tilde{x}_m^{p*} = 0, \tilde{x}_m^{r*} = 1; \tilde{x}_v^{p*} = x_v^{p*} - \Delta, \tilde{x}_v^{r*} = 1.$$

The new solution only has one category that has two non-zero values. The budget different is given

by:

$$\tilde{B} - B = (A^c_m - D^c_m)(1 - x^{p*}_m) + (D^c_v - A^c_v)\Delta.$$

To maintain the feasibility of the budget constraint, we set the value of the last equation to zero in order to determine the value of Δ:

$$\Delta = \frac{(A^c_m - D^c_m)(1 - x^{p*}_m)}{A^c_v - D^c_v}.$$

Next, we show the selected Δ remains the feasibility of $\tilde{x}^{p*}_v \leq 1$. Since $\delta > 1 - x^{p*}_m$, we have

$$\frac{(D^c_v - A^c_v)x^{p*}_v}{(D^c_m - A^c_m)} > 1 - x^{p*}_m,$$

which leads to:

$$(A^c_v - D^c_v)x^{p*}_v > (A^c_m - D^c_m)(1 - x^{p*}_m).$$

Therefore, $\Delta < x^{p*}_m$, which leads to $\tilde{x}^{p*}_v < 1$. Next, the difference of the new objective values between two solutions can be written as:

$$\begin{aligned}\tilde{f} - f =&(\tilde{A}_m - \tilde{D}_m)(1 - x^{p*}_m) + (\tilde{D}_v - \tilde{A}_v)\Delta \\ =&\left[(\tilde{A}_m - \tilde{D}_m) - (\tilde{A}_v - \tilde{D}_v)\frac{(A^c_m - D^c_m)}{A^c_v - D^c_v}\right]x^{p*}_m.\end{aligned}$$

Since $\frac{\tilde{A}_m - \tilde{D}_m}{A^c_m - D^c_m} \leq \frac{\tilde{A}_v - \tilde{D}_v}{A^c_v - D^c_v}$, we have

$$(\tilde{A}_m - \tilde{D}_m) - (\tilde{A}_v - \tilde{D}_v)\frac{A^c_m - D^c_m}{A^c_v - D^c_v} \leq 0.$$

Therefore, the inequality $\tilde{f} - f \leq 0$ suggests that the new solution attains a better objective value, contradicting the fact that the initial solution is optimal. □

Proof of Theorem 1. We prove the theorem by identifying how the optimal objective changes when given a small additional budget $\delta > 0$. The general proof idea is to evaluate the best contribution (the most negative one) within each category, when an additional budget is given. Subsequently, we demonstrate that it is optimal to choose the category offering the most negative contribution to the objective, out of all available categories. Let $f^*(B) = \sum_{m \in \mathcal{M}} f^*_m(B)$ represents the optimal objective value given budget B where f^*_m represents the optimal objective value of category m. Let v_m represent the best ratio of the objective to the cost in category m. The objective value of category m is given by:

$$f^*_m = \tilde{A}_m x^{p*}_m(B) + \tilde{D}_m(1 - x^{p*}_m(B))x^{r*}_m(B),$$

where $x^{p*}_m(B)$ and $x^{r*}_m(B)$ are the optimal solution of category m given budget B. The budget used by category m, denoted as $C^*_m(B)$, can be written as:

$$C^*_m(B) = A^c_m x^{p*}_m(B) + D^c_m(1 - x^{p*}_m(B))x^{r*}_m(B).$$

We consider four scenarios based on the coefficients of category m and investigate the best objective

contribution can be achieved within each category.

(i) $\frac{\tilde{A}_m}{A^c_m} \leq \frac{\tilde{D}_m}{D^c_m}$ and $\tilde{A}_m \leq \tilde{D}_m$: According to Lemma 4, within this scenario, it holds that $x^{r*}_m(B) = 0$ for any designated budget due to the dominance of proactive screening over reactive screening. As a result, we have $v_m(B) = \tilde{A}_m/A^c_m$, when $x^{r*}_m(B) < 1$. However, in cases where $x^{r*}_m(B) \geq 1$, v_m becomes 0, as there remains no potential for further improvement within category m.

(ii) $\frac{\tilde{A}_m}{A^c_m} \leq \frac{\tilde{D}_m}{D^c_m}$ and $\tilde{A}_m > \tilde{D}_m$: When $x^{p*}_m(B) < 1$, it is still optimal to first increase x^{p*}_m when setting $x^{r*}_m = 0$, as $\tilde{A}_m/A^c_m \leq \tilde{D}_m/D^c_m$, therefore $v_m = \tilde{A}_m/A^c_m$. When $x^{p*}_m(B) = 1$, according to Corollary 2, $x^{r*}_m(B)$ is binary under any given budget. Hence, the objective can only be further improved through the reduction of x^{p*}_m while maintaining $x^{r*}_m = 1$. In this context, the ratio of the objective to the cost becomes $v_m = (\tilde{A}_m - \tilde{D}_m)/(A^c_m - D^c_m)$.

(iii) $\frac{\tilde{A}_m}{A^c_m} \geq \frac{\tilde{D}_m}{D^c_m}$ and $\tilde{A}_m \geq \tilde{D}_m$: According to Lemma 4, under this scenario, $x^{p*}_m(B) = 0$ for any given budget as the reactive screening dominates proactive screening. Therefore, the best contribution per testing cost $v_m(B) = \tilde{D}_m/D^c_m$, if $x^{p*}_m(B) < 1$ or $C^*(B) < D^c_m$, otherwise $v_m = 0$ since there is no room of improvement left for category m.

(iv) $\frac{\tilde{A}_m}{A^c_m} \leq \frac{\tilde{D}_m}{D^c_m}$ and $\tilde{A}_m < \tilde{D}_m$: Under this scenario, when $C^*_m(B) \leq D^c_m$, it is optimal to increase x^{r*}_m when setting $x^{p*}_m = 0$. Therefore, we have $v_m = \tilde{D}_m/D^c_m$. When $C^*_m(B) > D^c_m$, according to Corollary 2,the objective can be further improved by increasing x^{p*}_m while keeping $x^{r*}_m = 1$, and the ratio of objective to the cost is equal to $v_m = (\tilde{D}_m - \tilde{A}_m)/(D^c_m - A^c_m)$.

When $B = 0$, both x^{p*}_m and x^{r*}_m are equal to zeros for all categories $m \in \mathcal{M}$. Building upon the aforementioned analysis, we can define the vector $\boldsymbol{v}$, which represents the best ratio of the objective and cost, can be defined as:

$$\boldsymbol{v} = \left[\min\left\{ \frac{\tilde{A}_1}{A^c_1}, \frac{\tilde{D}_1}{D^c_1}, 0 \right\}, \ldots, \min\left\{ \frac{\tilde{A}_M}{A^c_M}, \frac{\tilde{D}_M}{D^c_M}, 0 \right\} \right].$$

Notice that we introduce zero values to ensure the inclusion of negative entries. This consideration arises from the fact that if a category's best ratio is positive, any further improvement can not be achieved, given that it is a minimization problem. Let $\tilde{m}$ denote the selected category, where $\tilde{m} = \arg\min_m \{v_m\}$.

The procedure follows the greedy solution scheme of the fractional knapsack-style problem. That is, it is optimal to select the item with the most negative ratio of the objective value to the budget among all categories. The procedure repeats until either the budget is exhausted or $v_{\tilde{m}} \geq 0$, indicating a stopping condition. If $v_{\tilde{m}} = \tilde{A}_m/A^c_m$ and category m belongs to scenario (i), we set $x^{p^*}_m = \min\{1, B/A^c_{\tilde{m}}\}$ and update budget to $B = B - A^c_{\tilde{m}} x^{p^*}_m$. That is, x^{p*}_m either exhaust all budget or the budget is sufficient to set $x^{p*}_m = 1$. If $x^{p*}_m = 1$, according to the analysis in scenario (i), there is no further improvement can be achieved in category m, hence updating $v_m = 0$. On the other hand, if category m falls to scenario (ii), after set $x^{p*}_m = 1$, we set $v_m = (A_m - D_m)/(A^c_m - D^c_m)$. Similarly, if $v_{\tilde{m}} = \tilde{D}_m/D^c_m$ and category m belongs to scenario (iii), we set $x^{r^*}_m = \min\{1, B/D^c_{\tilde{m}}\}$ and update budget to $B = B - D^c_{\tilde{m}} x^{p^*}_m$. That is, x^{r*}_m either exhaust all budget or the budget is sufficient to set $x^{r*}_m = 1$. If $x^{r*}_m = 1$, according to the analysis in scenario (iii), there is no further improvement can be achieved in category m, hence updating $v_m = 0$. On the other hand, if category m falls to scenario (iv), after set $x^{r*}_m = 1$, we set $v_m = (D_m - A_m)/(D^c_m - A^c_m)$. If $v_{\tilde{m}} = (\tilde{A}_{\tilde{m}} - \tilde{D}_{\tilde{m}})/(A^c_{\tilde{m}} - D^c_{\tilde{m}})$

and $\tilde{A}_{\tilde{m}} > \tilde{D}_{\tilde{m}}$, $x_m^{p^*} = 1 - \min\{1, B/(D^c_{\tilde{m}} - A^c_{\tilde{m}})\}$ (exhaust all budget), $B = B - (D^c_{\tilde{m}} - A^c_{\tilde{m}})x_m^{p^*}$, and update $v_{\tilde{m}} = 0$ (since there is no room for further improvement). If $v_{\tilde{m}} = (\tilde{A}_{\tilde{m}} - \tilde{D}_{\tilde{m}})/(A^c_{\tilde{m}} - D^c_{\tilde{m}})$ and $\tilde{A}_{\tilde{m}} < \tilde{D}_{\tilde{m}}$, then set $x_m^{p^*} = \min\{1, B/(A^c_{\tilde{m}} - D^c_{\tilde{m}})\}$, $B = B - (A^c_{\tilde{m}} - D^c_{\tilde{m}})x_m^{p^*}$(exhaust all budget), and update $v_{\tilde{m}} = 0$ (since there is no room for further improvement).

□

Proof of Corollary 2. We prove the results by contradiction. Specifically, in cases where category m satisfies condition (i) while x_m^{r*} is non-binary (i.e.,$x_m^{r*} \in \{0, 1\}$), we can demonstrate that introducing a new solution with $x_m^{r*} \in \{0, 1\}$ will lead to an improvement in the objective value. Similarly, if category m meets condition (ii) with an optimal solution featuring $x_m^{p*} > 0$ and $x_m^{r*} \neq 1$, we can devise an improved solution by setting $x_m^{r*} = 1$.

(i) $\frac{\tilde{A}_m(n,\boldsymbol{z}^*|\lambda_m)}{A^c_m(n|\lambda_m)} \leq \frac{\tilde{D}_m(n,\boldsymbol{z}^*|\lambda_m)}{D^c_m(n|\lambda_m)}$ and $\tilde{A}_m(n, \boldsymbol{z}^*|\lambda_m) > \tilde{D}_m(n, \boldsymbol{z}^*|\lambda_m)$: Assume that the optimal solution for category m is $x_m^{p*} \in [0, 1]$ and $x_m^{r*} \in (0, 1)$. Consider a new solution:

$$\tilde{x}_m^{p*} = x_m^{p*} + \delta, \tilde{x}_m^{r*} = 0.$$

To maintain the budget feasibility, we first determine the value of δ. The budget difference between the original and the new solution is given by:

$$\begin{aligned}\tilde{B}_m - B_m =& A^c_m(x_m^{p*} + \delta) - \left[A^c_m x_m^{p*} + D^c_m(1 - x_m^{p*})x_m^{r*}\right] \\ =& A^c_m\delta - D^c_m(1 - x_m^{p*})x_m^{r*}.\end{aligned}$$

As such, we set $\tilde{B}_m - B_m = 0$ to make sure that the new solution does not violate the budget constraint, which leads to

$$\delta = \frac{D^c_m(1 - x_m^{p*})x_m^{r*}}{A^c_m}.$$

To maintain the feasibility of $\tilde{x}_m^{p*}$, we consider two sub-scenarios: (ia) $\delta \leq 1 - x_m^{p*}$ and (ib) $\delta > 1 - x_m^{p*}$.

(ia) $\delta \leq 1 - x_m^{p*}$: The objective of the new function is given by:

$$\begin{aligned}\tilde{f} - f &= \tilde{A}_m \frac{D^c_m(1 - x_m^{p*})x_m^{r*}}{A^c_m} - \tilde{D}_m(1 - x_m^{p*})x_m^{r*} \\ &= \left[\frac{A_m}{A^c_m} D^c_m - \tilde{D}_m\right](1 - x_m^{p*})x_m^{r*}.\end{aligned}$$

Since $\tilde{A}_m/A^c_m \leq \tilde{D}_m/D^c_m$, we know that the coefficient of last equation is negative. Therefore, we can conclude that $\tilde{f} - f \leq 0$, which contradicts the assumption that x_m^{p*} and x_m^{r*} are the optimal solutions. These results demonstrate that the newly constructed solution with $x_m^{r*} = 1$ achieves a better objective.

(ib) $\delta > 1 - x_m^{p*}$: We consider a new optimal solution:

$$\tilde{x}_m^{p*} = \Delta, \tilde{x}_m^{r*} = 1.$$

To maintain the budget feasibility, we first determine the value of δ. The budget difference between

the original and the new solution is given by:

$$\begin{aligned}\tilde{B}_m - B_m =& \Big[A_m^c \Delta + D_m^c (1-\Delta)\Big] - \Big[A_m^c x_m^{p*} + D_m^c (1-x_m^{p*}) x_m^{r*}\Big] \\ =& (A_m^c - D_m^c)\Delta - A_m^c x_m^p + D_m^c \big[1-(1-x_m^{p*})x_m^{r*}\big].\end{aligned}$$

As such, we set $\tilde{B}_m - B_m = 0$ to make sure that the new solution does not violate the budget constraint, which leads to

$$\Delta = \frac{A_m^c x_m^p - D_m^c \big[1-(1-x_m^{p*})x_m^{r*}\big]}{A_m^c - D_m^c}.$$

Since $\delta > 1 - x_m^{p*}$, we have

$$\delta = \frac{D_m^c (1-x_m^{p*}) x_m^{r*}}{A_m^c} > 1 - x_m^{p*},$$

which leads to

$$D_m^c (1-x_m^{p*}) x_m^{r*} > A_m^c (1-x_m^{p*}).$$

As such, we have

$$\Delta = \frac{A_m^c x_m^p - D_m^c \big[1-(1-x_m^{p*})x_m^{r*}\big]}{A_m^c - D_m^c} < \frac{D_m^c - A_m^c}{D_m^c - A_m^c} = 1.$$

Therefore, the feasibility of $\tilde{x}_m^{p*} \in [0,1]$ is satisfied. The objective of the new function is given by:

$$\begin{aligned}\tilde{f} - f &= (\tilde{D}_m - \tilde{A}_m) \frac{D_m^c (1-x_m^{p*})(1-x_m^{r*})}{A_m^c - D_m^c} + \tilde{D}_m (1-x_m^{p*})(1-x_m^{r*}) \\ &= \left[\tilde{D}_m - \frac{\tilde{D}_m - \tilde{A}_m}{D_m^c - A_m^c} D_m^c\right] (1-x_m^{p*})(1-x_m^{r*}),\end{aligned}$$

where the coefficient can be written as

$$\begin{aligned}\tilde{D}_m - \frac{\tilde{D}_m - \tilde{A}_m}{D_m^c - A_m^c} D_m^c =& \frac{(D_m^c - A_m^c)\tilde{D}_m - (\tilde{D}_m - \tilde{A}_m) D_m^c}{D_m^c - A_m^c} \\ =& \frac{\tilde{A}_m D_m^c - A_m^c \tilde{D}_m}{D_m^c - A_m^c}.\end{aligned}$$

Since $\tilde{A}_m / A_m^c \leq \tilde{D}_m / D_m^c$ and $D_m^c > A_m^c$, we can conclude that $\tilde{f} - f \leq 0$, which contradicts the assumption that x_m^{p*} and x_m^{r*} are the optimal solutions.

(ii) $\frac{\tilde{A}_m(n,\boldsymbol{z}^*|\lambda_m)}{A_m^c(n|\lambda_m)} \geq \frac{\tilde{D}_m(n,\boldsymbol{z}^*|\lambda_m)}{D_m^c(n|\lambda_m)}$ and $\tilde{A}_m(n,\boldsymbol{z}^*|\lambda_m) < \tilde{D}_m(n,\boldsymbol{z}^*|\lambda_m)$: Assume that the optimal solution for category m is $x_m^{p*} > 0$ and $x_m^{r*} \in [0,1)$. Consider a new solution:

$$\tilde{x}_m^{p*} = x_m^{p*} - \delta, \tilde{x}_m^{r*} = 1.$$

To maintain the budget feasibility, we first determine the value of δ. The budget difference between the original and the new solution is given by:

$$\begin{aligned}\tilde{B}_m - B_m =& \Big[A_m^c (x_m^{p*} - \delta) + D_m^c (1 - x_m^{p*} + \delta)\Big] - \Big[A_m^c x_m^{p*} + D_m^c (1-x_m^{p*}) x_m^{r*}\Big] \\ =& (D_m^c - A_m^c)\delta + D_m^c (1-x_m^{p*})(1-x_m^{r*}).\end{aligned}$$

As such, we set $\tilde{B}_m - B_m = 0$ to make sure that the new solution does not violate the budget constraint, which leads to

$$\delta = \frac{D_m^c(1 - x_m^{p*})(1 - x_m^{r*})}{A_m^c - D_m^c}.$$

To maintain the feasibility of $\tilde{x}_m^{p*}$, we consider two sub-scenarios: (iia) $\delta \leq x_m^{p*}$ and (iib) $\delta > x_m^{p*}$.

(iia) $\delta \leq x_m^{p*}$: The objective difference is given by:

$$\begin{aligned}
\tilde{f} - f &= (\tilde{D}_m - \tilde{A}_m)\frac{D_m^c(1 - x_m^{p*})(1 - x_m^{r*})}{A_m^c - D_m^c} + \tilde{D}_m(1 - x_m^{p*})(1 - x_m^{r*}) \\
&= \left[\tilde{D}_m - \frac{\tilde{D}_m - \tilde{A}_m}{D_m^c - A_m^c}D_m^c\right](1 - x_m^{p*})(1 - x_m^{r*}),
\end{aligned}$$

where the coefficient can be written as

$$\tilde{D}_m - \frac{\tilde{D}_m - \tilde{A}_m}{D_m^c - A_m^c}D_m^c = \frac{\tilde{A}_m D_m^c - A_m^c \tilde{D}_m}{D_m^c - A_m^c}.$$

Since $\tilde{A}_m/A_m^c \geq \tilde{D}_m/D_m^c$ and $D_m^c < A_m^c$, we can conclude that $\tilde{f} - f \leq 0$, which contradicts the assumption that x_m^{p*} and x_m^{r*} are the optimal solutions. □

(iib) $\delta > x_m^{p*}$: We consider a new solution:

$$\tilde{x}_m^{p*} = 0, \tilde{x}_m^{r*} = \Delta.$$

The budget difference is given by:

$$\tilde{B}_m - B_m = D_m^c\Delta - \left[A_m^c x_m^{p*} + D_m^c(1 - x_m^{p*})x_m^{r*}\right].$$

To maintain the feasibility of the constraint, we set Δ as

$$\Delta = \frac{A_m^c x_m^{p*} + D_m^c(1 - x_m^{p*})x_m^{r*}}{D_m^c}.$$

Since $\delta > x_m^{p*}$, we have

$$\delta = \frac{D_m^c(1 - x_m^{p*})(1 - x_m^{r*})}{A_m^c - D_m^c} > x_m^{p*},$$

which leads to

$$A_m^c x_m^{p*} + D_m^c(1 - x_m^{p*})x_m^{r*} \leq D_c.$$

Therefore, the feasibility of $\tilde{x}_m^{r*} \in [0, 1]$ is satisfied. The objective difference is given by:

$$\tilde{f} - f = \tilde{D}_m\Delta - \left[\tilde{A}_m x_m^{p*} + \tilde{D}_m(1 - x_m^{p*})x_m^{r*}\right] = \frac{\tilde{D}_m A_m^c - \tilde{A}_m D_m^c}{D_m^c}x_m^{p*}.$$

Since $\tilde{A}_m/A_m^c \geq \tilde{D}_m/D_m^c$, we have $\tilde{D}_m A_m^c - \tilde{A}_m D_m^c \leq 0$. Therefore, we can conclude that $\tilde{f} - f \leq 0$, which contradicts the assumption that x_m^{p*} and x_m^{r*} are optimal.

D Structural Properties of CRP-MS($\boldsymbol{z}^*$)

In this section, we present the modified structural properties of **CRP-MS**($\boldsymbol{z}^*$), building upon the lemmas established by **RP-MS**($\boldsymbol{n}, \boldsymbol{z}^*$) in Section 3.

Lemma 2 (**CRP-MS**($\boldsymbol{z}^*$)). *For a given $\boldsymbol{\lambda} = [\lambda_m]$, the optimal solution of **CRP-MS**($\boldsymbol{z}^*$) will never utilize a budget greater than:*

$$\overline{B}_{\boldsymbol{\lambda}}(\boldsymbol{n}, \boldsymbol{z}^*) = \sum_{m \in \mathcal{M}(\boldsymbol{n}, \boldsymbol{z}^*, \boldsymbol{\lambda})} \left[A^c_m(n^{p*}_m) \cdot t_m(n, \boldsymbol{z}^*|\lambda_m) + D^c_m(n^{r*}_m) \cdot (1 - t_m(n, \boldsymbol{z}^*|\lambda_m)) \right],$$

where

$$\mathcal{M}(\boldsymbol{n}, \boldsymbol{z}^*, \boldsymbol{\lambda}) = \left\{ m \in \mathcal{M} : \min\{\tilde{A}_m(n^{p*}_m, \boldsymbol{z}^*|\lambda_m), \tilde{D}_m(n^{r*}_m, \boldsymbol{z}^*|\lambda_m)\} < 0 \right\} \subseteq \mathcal{M},$$

$t_m(\boldsymbol{n}, \boldsymbol{z}^*|\lambda_m) = 1$ *if* $\tilde{A}_m(n^{p*}_m, \boldsymbol{z}^*|\lambda_m) < \tilde{D}_m(n^{r*}_m, \boldsymbol{z}^*|\lambda_m)$, 0 *otherwise for all* $m \in \mathcal{M}(\boldsymbol{n}, \boldsymbol{z}^*, \boldsymbol{\lambda})$, $n^{p*}_m \in \arg\min_n\{n \in N^p : \tilde{A}_m(n, \boldsymbol{z}^*|\lambda_m)\}$ *and* $n^{r*}_m \in \arg\min_n\{n \in N^r : \tilde{D}_m(n, \boldsymbol{z}^*|\lambda_m)\}$.

Lemma 3 (**CRP-MS**($\boldsymbol{z}^*$)). *For a given $\boldsymbol{\lambda} = [\lambda_m]$, define $t_{\underline{m}}(\boldsymbol{n}, \boldsymbol{z}|\lambda_{\underline{m}}) = 1$ if $A_{\underline{m}}(n^{p*}_{\underline{m}}, \boldsymbol{z}|\lambda_m)/A^c_{\underline{m}}(n^{p*}_{\underline{m}}|\lambda_m) < D_{\underline{m}}(n^{r*}_{\underline{m}}, \boldsymbol{z}|\lambda_m)/D^c_{\underline{m}}(n^{p*}_{\underline{m}}|\lambda_m)$, 0 otherwise, where $n^{p*}_m \in \arg\min_n\{n \in N^p : \tilde{A}_m(n, \boldsymbol{z}^*|\lambda_m)/A^c_m(n|\lambda_m)\}$ and $n^{r*}_m \in \arg\min_n\{n \in N^r : \tilde{D}_m(n, \boldsymbol{z}^*|\lambda_m)/D^c_m(n|\lambda_m)\}$. The lower bound on budget level for Problem **CRP-MS**($\boldsymbol{z}^*$) is defined as:*

$$\underline{B}_{\lambda}(\boldsymbol{n}, \boldsymbol{z}^*) = A^c_{\underline{m}}(n^{p*}_m|\lambda_{\underline{m}}) \cdot t_{\underline{m}}(\boldsymbol{n}, \boldsymbol{z}^*|\lambda_{\underline{m}}) + D^c_{\underline{m}}(n^{r*}_m|\lambda_{\underline{m}}) \cdot (1 - t_{\underline{m}}(\boldsymbol{n}, \boldsymbol{z}^*|\lambda_{\underline{m}})),$$

where $\underline{m}$ is given by:

$$\underline{m} \in \arg\min_{m \in \mathcal{M}} \left\{ \frac{\tilde{A}_m(n^{p*}_m, \boldsymbol{z}^*|\lambda_m)}{A^c_m(n^{p*}_m|\lambda_m)} \cdot t_{\underline{m}}(\boldsymbol{n}, \boldsymbol{z}^*|\lambda_m) + \frac{\tilde{D}_m(n^{r*}_m, \boldsymbol{z}^*|\lambda_m)}{D^c_m(n^{r*}_m|\lambda_m)} \cdot (1 - t_{\underline{m}}(\boldsymbol{n}, \boldsymbol{z}^*|\lambda_m)) \right\}.$$

*When $B \leq \underline{B}_{\boldsymbol{\lambda}}(\boldsymbol{n}, \boldsymbol{z}^*)$, the optimal solution of **CRP-MS**($\boldsymbol{z}^*$) is given by:*

$$(\boldsymbol{u}^*, \boldsymbol{v}^*) = \begin{cases} u^{n^{p*}}_m = \frac{B}{A^c_m(n^{p*}_m|\lambda_m)} \cdot t_m(\boldsymbol{n}, \boldsymbol{z}^*|\lambda_m), v^{n^{r*}}_m = \frac{B}{D^c_m(n^{r*}_m|\lambda_m)} \cdot (1 - t_m(\boldsymbol{n}, \boldsymbol{z}^*|\lambda_m)), & \text{if } m = \underline{m} \\ u^{n^{p*}}_m = v^{n^{r*}}_m = 0, & \text{otherwise.} \end{cases}$$

Lemma 4 (**CRP-MS**($\boldsymbol{z}^*$)). *For a given $\boldsymbol{\lambda} = [\lambda_m]$, the following two statements must hold for the optimal solution of **CRP-MS**($\boldsymbol{z}^*$) for category m under any given budget:*

(i) If $\frac{\tilde{A}_m(n^1, \boldsymbol{z}^*|\lambda_m)}{A^c_m(n^1|\lambda_m)} \leq \frac{\tilde{A}_m(n^2, \boldsymbol{z}^*|\lambda_m)}{A^c_m(n^2|\lambda_m)}$ *and* $\tilde{A}_m(n^1, \boldsymbol{z}^*|\lambda_m) \leq \tilde{A}_m(n^2, \boldsymbol{z}^*|\lambda_m)$, *then* $u^{n^{2*}}_m = 0$.

(ii) If $\frac{\tilde{D}_m(n^1, \boldsymbol{z}^*|\lambda_m)}{D^c_m(n^1|\lambda_m)} \leq \frac{\tilde{D}_m(n^2, \boldsymbol{z}^*|\lambda_m)}{D^c_m(n^2|\lambda_m)}$ *and* $\tilde{D}_m(n^1, \boldsymbol{z}^*|\lambda_m) \leq \tilde{D}_m(n^2, \boldsymbol{z}^*|\lambda_m)$, *then* $v^{n^{2*}}_m = 0$.

(iii) If $\frac{\tilde{A}_m(n, \boldsymbol{z}^*|\lambda_m)}{A^c_m(n|\lambda_m)} \leq \frac{\tilde{D}_m(n, \boldsymbol{z}^*|\lambda_m)}{D^c_m(n|\lambda_m)}$ *and* $\tilde{A}_m(n, \boldsymbol{z}^*|\lambda_m) \leq \tilde{D}_m(n, \boldsymbol{z}^*|\lambda_m)$, *then* $v^{n^*}_m = 0$.

(iv) If $\frac{\tilde{A}_m(n, \boldsymbol{z}^*|\lambda_m)}{A^c_m(n|\lambda_m)} > \frac{\tilde{D}_m(n, \boldsymbol{z}^*|\lambda_m)}{D^c_m(n|\lambda_m)}$ *and* $\tilde{A}_m(n, \boldsymbol{z}^*|\lambda_m) > \tilde{D}_m(n, \boldsymbol{z}^*|\lambda_m)$, *then* $u^{n^*}_m = 0$.

E Heuristic Solution Scheme for CRP-MS ($\boldsymbol{z}^*$)

Data: $r_m, s_m, \alpha_m, \lambda_m, z_m^{s*}, z_m^{\bar{s}*}$ $\forall m \in \mathcal{M}$, Se, Sp, B, N, $\mathcal{N} = \{1, \ldots, N\}$;
Output: $\boldsymbol{u}^*, \boldsymbol{v}^*$, Obj^* ;
Initialization: Compute coefficients $A_m(n), D_m(n), A_m^c(n), D_m^c(n)$;
for $m \in \mathcal{M}, n \in \mathcal{N}$ **do**
    $RList \leftarrow A_m(n)/A_m^c(n)$ and $D_m(n)/D_m^c(n)$;
    $VList \leftarrow A_m(n)$ and $D_m(n)$; $WList \leftarrow A_m(n)$ and $D_m(n)$;
Sort $RList$ in descending order and add sorted index tuple (m, n) in SortedIndex;
$ViolateN \leftarrow 1$;
while $ViolateN > 0$ **do**
    for (m, n) *in SortedIndex; Start with* $(\tilde{m}, \tilde{n})$ **do**
        if $B > 0$ **then**
            if $B > WList[m][n]$ **then**
                if $VList[m][n] = A_m(n)$ **then**
                    Set $u[m][n] = 1$; $B = B - WList[m][n]$; $Obj = Obj + VList[m][n]$;
                else
                    Set $v[m][n] = 1$; $B = B - WList[m][n]$; $Obj = Obj + VList[m][n]$;
                    $(\tilde{m}, \tilde{n}) = (m, n)$;
            else
                if $VList[m][n] = D_m(n)$ **then**
                    Set $u[m][n] = B/WList[m][n]$;$B = 0$; $Obj = Obj + VList[m][n]$;
                else
                    Set $v[m][n] = B/WList[m][n]$; $B = 0$; $Obj = Obj + VList[m][n]$;
                    $(\tilde{m}, \tilde{n}) = (m, n)$
    $ViolateN \leftarrow 0$
    for $m \in \mathcal{M}$ **do**
        if $sum(u[m]) > 1$ **then**
            Select $n^* \in \{n \in \mathcal{N} : u[m][n] \neq 0\}$ with the smallest value of $A_m(n) \cdot u[m][n^*]$;
            $B = B + A_m^c(n^*) \cdot u[m][n^*]$; $Obj = Obj - A_m(n^*) \cdot u[m][n^*]$; $u[m][n] = 0\ \forall n \neq n^*$;
            $ViolateN + = 1$;
        if $sum(v[m]) > 1$ **then**
            Select $n^* \in \{n \in \mathcal{N} : v[m][n] \neq 0\}$ with the smallest value of $v[m][n^*]$;
            $B = B + D_m^c(n^*) \cdot v[m][n^*]$; $Obj = Obj - D_m(n^*) \cdot v[m][n^*]$; $v[m][n] = 0\ \forall n \neq n^*$;
            $ViolateN + = 1$
    for $m \in \mathcal{M}$ **do**
        if $sum(u[m]) > 0$ & $sum(v[m]) > 0$ **then**
            Select $n^u \in \{n \in \mathcal{N} : u[m][m] \neq 0\}$; $n^v = \{n \in \mathcal{N} : v[m][m] \neq 0\}$;
            if $A_m(n^u) < D_m(n^v)$ **then**
                Set $B = B + D_m^c(n^v) \cdot v[m][n^v] = 0$; $Obj = Obj - D_m(n^v) \cdot v[m][n^u]$;
                $v[m][n^v] = 0$; $ViolateN + = 1$;
            else
                Set $B = B + A_m^c(n^u) \cdot u[m][n^v] = 0$; $Obj = Obj - A_m(n^u) \cdot u[m][n^u]$;
                $u[m][n^u] = 0$; $ViolateN + = 1$;
Return $\boldsymbol{u}^*, \boldsymbol{v}^*$, Obj^*.

F Calibration of Symptomatic Rates

Assume the overall symptomatic rate of a disease (COVID-19 or flu) is $\tilde{S}$ and let U_w be a binary indicator with value 1 indicating subject w having underlying health conditions and 0 otherwise. Conditioning on the underlying conditions of a random subject w, the symptomatic rate is given by:

$$P\big(s_w = 1\big) = P\big(s_w = 1 \big| U_w = 1\big) P(U_w = 1) + P\big(s_w = 1 \big| U_w = 0\big) P(U_w = 0).$$

In addition, given that the subject with underlying conditions are more likely to show symptoms by β, we have:

$$P\big(s_w = 1\big) = P\big(s_w = 1 \big| U_w = 1\big) P(U_w = 1) + \frac{1}{\beta} P\big(s_w = 1 \big| U_w = 1\big) P(U_w = 0).$$

As such, the symptomatic rate with underlying conditions is given by:

$$P\big(s_w = 1 \big| U_w = 1\big) = \frac{P\big(s_w = 1\big)}{P(U_w = 1) + \frac{1}{\beta} P(U_w = 0)}. \tag{96}$$

Then, the symptomatic rate with no underlying conditions is given by:

$$P\big(s_w = 1 \big| U_w = 0\big) = \frac{1}{\beta} P\big(s_w = 1 \big| U_w = 1\big) = \frac{P\big(s_w = 1\big)}{\beta P(U_w = 1) + P(U_w = 0)}, \tag{97}$$

where $P(U_w = 1)$ and $P(U_w = 0)$ can be obtained by the prevalence rate of underlying conditions. In our case study, for COVID-19, we use the nationwide prevalence rate of selected underlying conditions as 41.44%, the overall symptomatic rate as 64.9%. Furthermore, the study found that individuals with at least one underlying condition are two times more likely to experience severe symptoms [1], leading to $\beta = 2$. Then, Eq. (96) and (97) are given by:

$$P\big(s_w = 1 \big| U_w = 1\big) = 81.30\%,$$

and

$$P\big(s_w = 1 \big| U_w = 1\big) = 40.65\%.$$

□

S1 File Reference

1. Yu C, Zhou M, Liu Y, Guo T, Ou C, Yang L, et al. Characteristics of asymptomatic COVID-19 infection and progression: a multicenter, retrospective study. Virulence, 11(1), 1006-1014.
